# Analysis and Identification of Aptamer-Compound Interactions with a Maximum Relevance Minimum Redundancy and Nearest Neighbor Algorithm

**DOI:** 10.1155/2016/8351204

**Published:** 2016-02-03

**Authors:** ShaoPeng Wang, Yu-Hang Zhang, Jing Lu, Weiren Cui, Jerry Hu, Yu-Dong Cai

**Affiliations:** ^1^School of Life Sciences, Shanghai University, Shanghai 200444, China; ^2^Institute of Health Sciences, Shanghai Institutes for Biological Sciences, Chinese Academy of Sciences, Shanghai 200031, China; ^3^Key Laboratory of Molecular Pharmacology and Drug Evaluation (Ministry of Education), Collaborative Innovation Center of Advanced Drug Delivery System and Biotech Drugs in Universities of Shandong, School of Pharmacy, Yantai University, Shandong, Yantai 264005, China; ^4^CAS-MPG Partner Institute for Computational Biology, Shanghai Institutes for Biological Sciences, Chinese Academy of Sciences, Shanghai 200031, China; ^5^Department of Mathematics and Computer Science, School of Arts and Sciences, University of Houston-Victoria, Victoria, TX 77901, USA

## Abstract

The development of biochemistry and molecular biology has revealed an increasingly important role of compounds in several biological processes. Like the aptamer-protein interaction, aptamer-compound interaction attracts increasing attention. However, it is time-consuming to select proper aptamers against compounds using traditional methods, such as exponential enrichment. Thus, there is an urgent need to design effective computational methods for searching effective aptamers against compounds. This study attempted to extract important features for aptamer-compound interactions using feature selection methods, such as Maximum Relevance Minimum Redundancy, as well as incremental feature selection. Each aptamer-compound pair was represented by properties derived from the aptamer and compound, including frequencies of single nucleotides and dinucleotides for the aptamer, as well as the constitutional, electrostatic, quantum-chemical, and space conformational descriptors of the compounds. As a result, some important features were obtained. To confirm the importance of the obtained features, we further discussed the associations between them and aptamer-compound interactions. Simultaneously, an optimal prediction model based on the nearest neighbor algorithm was built to identify aptamer-compound interactions, which has the potential to be a useful tool for the identification of novel aptamer-compound interactions. The program is available upon the request.

## 1. Introduction

Aptamers are defined as single-stranded nucleic acids or peptides that act like antibodies [1, 2]. These specific selective molecules can easily recognize and identify certain targets in the proper environment. In vitro, aptamers are widely artificially selected from a large random sequence pool; at the same time, natural aptamers always exist in the riboswitches [3]. Compared to antibodies, these artificial molecules have several advantages. Apart from their high affinity and wide range of applications, it is much easier to screen and accurately amplify aptamers than antibodies. With the development of molecular biology techniques, it is even possible for us to modify the aptamers after screening, which may be much harder for antibodies. Moreover, purification is always difficult and cumbersome in molecular technology. However, polymerase chain reaction makes it amazingly easy to attain quantities of target aptamers without a complex purification process [4]. All in all, aptamers are a potentially valuable class of ligands that are sure to be widely used in the fields of biology and medicine [5].

Previous studies have focused on aptamer-protein interactions [6]. With the development of biochemistry and molecular biology, compounds have been shown to play an increasingly significant role in several biological processes; therefore, it is necessary to focus on aptamer-compound interactions. The most widely used method to select aptamers is systematic evolution of ligands by exponential enrichment (SELEX) [1, 2]. Similar to aptamer-protein interactions, SELEX is also used to select proper aptamers against compounds [7, 8]. However, aptamers are highly target-specific and environment dependent. As a result, selecting proper aptamers from random combinatorial libraries is monotonously repetitive and inefficient. A proper, high affinity aptamer takes months or even years to be screened. Currently, we can design effective computational methods to screen proper aptamers. In this study, we analyzed the mechanism underlying aptamer-compound interactions by synthesizing characteristics of both the compounds and the aptamers. To encode each investigated interaction into a numerical vector that can be processed by computers, the constitutional, electrostatic, quantum-chemical, and space conformational descriptors of the compounds were taken into consideration, as was the nucleotide composition of the aptamers. Then, like the aptamer-protein feature selection reported in a previous study [9], the Maximum Relevance Minimum Redundancy (mRMR) method and the Incremental Feature Selection (IFS) method were applied to screen the optimal features for the determination of aptamer-compound interactions. Simultaneously, an optimal prediction model based on the nearest neighbor algorithm (NNA) was built. Our results may help broaden the applications of aptamers in biological and medical fields.

## 2. Materials and Methods

### 2.1. Materials

Aptamer Base (http://aptamerbase.semanticscience.org/) is a collaboratively created and maintained knowledge base about aptamers, including their interactions and detailed experimental conditions with citations to primary scientific literature [10]. It contains a total of 1,994 entries of interactions (accessed in May 2014), in which 1,335 entries involve one or more compounds. After searching the 1,335 entries, we obtained 1,507 interactions between aptamers and compounds. Moreover, because of the extension of freebase itself, it is easy to obtain compound information from another freebase “compound.” Most of the “compound IDs” and some SMILE strings were also available from direct query on this freebase.

To obtain a well-defined dataset, 1,507 aptamer-compound interactions were further refined using the following rules: (1) interactions containing compounds whose Pubchem IDs were not available were excluded; (2) interactions containing compounds whose molecular weights are greater than 800 were removed because it is time-consuming to make structural optimization by AMPAC for compounds with high molecular weights; and (3) interactions containing compounds that cannot match the SMILE strings were also removed. Finally, we obtained 159 aptamer-compound interactions, involving 20 compounds and 156 aptamers. These 159 aptamer-compound interactions were considered to be positive interactions in this study.

To characterize features of aptamer-compound interactions, the negative data were also necessary, constructed according to the following rules: (1) randomly combine one compound from 20 compounds and one aptamer from 156 aptamers to constitute an interaction; (2) the constructed interactions were not positive interactions. Because the possibility of one compound and one aptamer being an actual aptamer-compound interaction is very low, we randomly produced 318 negative interactions, which was twice as many as the positive interactions. The positive and negative interactions are all provided in Supplemental Material I, available online at http://dx.doi.org/10.1155/2016/8351204.

### 2.2. Representation of Aptamer-Compound Interactions

To build an effective prediction model, encoding each sample with its essential properties is one of the most important steps. In this study, we encoded each aptamer by the nucleotide composition and compound using descriptors, including constitutional, topological, geometric, electrostatic, and quantum-chemical features.

#### 2.2.1. Aptamer Representation

The frequencies of single nucleotides (“a,” “c,” “g,” and “u(t)”) and dinucleotides (“aa,” “ac,” “ag,” “au(t),” “ca,” “cc,” “cg,” “cu(t),” “ga,” “gc,” “gg,” “gu(t),” “u(t)a,” “u(t)c,” “u(t)g,” and “u(t)u(t)”) were used to encode each aptamer. Thus, each investigated aptamer can be represented by a 20D (20-dimensional) numerical vector.

#### 2.2.2. Compound Representation

The initial structures of all compounds were optimized by Sybyl 6.8 [11], and structural optimization was performed using the AM1 semiempirical method implemented in AMPAC 8.16 [12]. To describe the characteristics of the compounds, a total of 499 descriptors, including constitutional, topological, geometric, electrostatic, and quantum-chemical features, were calculated with Codessa 2.7.2 [13]. After removing those descriptors with zero variance or missing values for some compounds, 301 descriptors remained. The distribution of these 301 descriptors is listed in Table 1. As a result, each investigated compound was represented by a 301D (301-dimensional) numerical vector.

#### 2.2.3. Interaction Representation

Because each interaction consisted of one aptamer and one compound, it can be represented by a 321D (321-dimensional) numerical vector, where 20 components represented the properties of aptamers and the others represented the properties of compounds (see Table 1).

### 2.3. mRMR

As mentioned in Section 2.2, 321 features represented each aptamer-compound interaction. Clearly not all features equally contribute to the identification of actual aptamer-compound interactions. Some of features make key contributions, whereas some others are less important. To analyze the features, a popular feature selection method, mRMR, which was first proposed by Peng et al. [14] in 2005, was employed. This method measures the investigated features for a certain problem by providing two lists, MaxRel features list and mRMR features list. The MaxRel features list sorts the investigated features by their contributions into classifications, that is, with relevance to class labels. The mRMR features list sorts features by considering not only their contributions to classification but also the redundancies to features listed before them. The detailed descriptions are as follows. Firstly, the above factors can be encoded into numbers using the mutual information (MI), which can be calculated by

(1) $$\begin{array}{c} I\left(\;x,y\;\right)=\iint p\left(\;x,y\;\right)\log\frac{p\left(x,y\right)}{p\left(x\right)p\left(y\right)}dx\;dy, \end{array}$$

where *x* and *y* represent two variables, *p*(*x*, *y*) represents the joint probabilistic density of *x* and *y*, and *p*(*x*) represents the marginal probabilistic density of variable *x*.

For a problem involving *N* features, the MI of each feature as well as the target vector, consisting of samples class labels, is calculated. The MaxRel features list ranks the features with the descending order of MI values. For the mRMR features list, because it additionally considers the redundancies between features, it is produced using a loop procedure. Suppose *Ω* is a set containing *N* features and *Ω*_*s*_ is a set consisting of already selected features (initially, *Ω*_*s*_ = Φ) and *Ω*_*t*_ consists of the rest features; that is, *Ω*_*t*_ = *Ω* − *Ω*_*s*_. The contribution of feature *f* in *Ω*_*t*_ is measured using the MI of it and target vector *c*, that is, *D* = *I*(*f*, *c*), while the redundancies between it and features in *Ω*_*s*_ are measured by *R* = (1/|*Ω*_*s*_|)∑_*f*_*i*_∈*Ω*_*s*__ *I*(*f*, *f*_*i*_) (if *Ω*_*s*_ = Φ, *R* is set to zero). To select a feature with maximum contributions for classification and minimum redundancies between it and features in *Ω*_*s*_, the feature yielding the maximum *D*-*R* will be selected in the next loop and removed from *Ω*_*t*_ to *Ω*_*s*_. When all features are in *Ω*_*s*_, the loop stops. The mRMR features list ranks features using the selection sequence of features.

By analyzing the MaxRel features list and mRMR features list, we can extract important features among the investigated features and build an optimal prediction model based on one machine learning algorithm. Currently, the mRMR method has been applied to study a number of biological problems and some optimal classification models have been built [15–24]. Here, we denoted the MaxRel features list and the mRMR features list as follows:

(2) MaxRel features list:  FMaxRel=f1M,f2M,…,fnM,mRMR features list:  FmRMR=f1m,f2m,…,fnm.

For a detailed description of this method, please refer to Peng et al.'s [14] or visit the website http://home.penglab.com/software/Hanchuan_Peng_Software/software.html.

### 2.4. Basic Prediction Engine

Based on the mRMR features list obtained by the mRMR method and a basic prediction engine, one can construct an optimal prediction model using key features to represent samples. Here, we tried four prediction engines: (1) NNA [25]; (2) Random Forest (RF) [26]; (3) Sequential Minimal Optimization (SMO) [27]; (4) Dagging [28]. Their brief descriptions were as follows.

#### 2.4.1. NNA

NNA is a classic classifier. Although it is simple, it performs well in many cases [29–32]. For a query sample, the distances between it and samples in the training set are computed and the class of the sample with the minimum distance is assigned to it.

#### 2.4.2. RF

RF is an ensemble classifier proposed by Breiman [26]. It integrates a number of decision trees, which are constructed by randomly selecting samples from the original training set and randomly selecting features to split each node. Because it contains two procedures of random selections, it always yields good performance and has been applied to deal with many biological problems [33–37].

#### 2.4.3. SMO

SMO is a type of support vector machines (SVM) that is optimized by the John Platt's Sequential Minimal Optimization algorithm [27]. The optimization problem of SVM is divided into several of the smallest possible subproblems, and they are solved analytically.

#### 2.4.4. Dagging

Dagging is a metaclassifier containing multiple prediction models that are derived from a number of disjoint subsets of the original training set and a single machine learning algorithm [28]. Its predicted result integrated the results of the prediction models by majority voting.

In Weka [38], four classifiers (IB1, Random Forest, SMO, and Dagging) implement the above four methods. For convenience, they were employed to make classifications and they were all executed with their default parameters.

### 2.5. Accuracy Measurement

Identification of aptamer-compound interactions is a two-class classification problem. To measure the performance of a classifier on this type of problem, four values were counted, true positive (TP), true negative (TN), false positive (FP), and false negative (FN) [29, 39]. Furthermore, these values can be used to calculate the following measurements:

(3) SN=TPTP+FN,SP=TNTN+FP,ACC=TP+TNTP+TN+FP+FN,MCC=TP·TN−FP·FNTN+FN·TN+FP·TP+FN·TP+FP.

To correctly measure the performance of a classifier, one measurement listed in (3) should be selected as the key measurement. Obviously, SN and SP are not perfect measurements because they only partly use TP, TN, FP, and FN. Regarding ACC and MCC [40], we prefer to use MCC as the key measurement because MCC is a balanced measurement even if the number of samples in each class greatly differs. Therefore, in this study, MCC is always used to measure the performance of the current prediction method, whereas SN, SP, and ACC are provided as reference.

### 2.6. IFS

By combining the mRMR features list and a basic prediction engine (e.g., NNA and RF), one can build an optimal prediction model, in which each sample is represented by extracted key features and the adopted basic prediction engine provides the best performance. This procedure is called IFS, which can be implemented as follows:

(i) Based on the mRMR feature list *F*_mRMR_ = [*f*_1_^*m*^, *f*_2_^*m*^,…, *f*_*n*_^*m*^], *N* feature sets were constructed such that IFS_*i*_ = {*f*_1_^*m*^, *f*_2_^*m*^,…, *f*_*i*_^*m*^} (1 ≤ *i* ≤ *n*).
(ii) For the *i*th feature sets IFS_*i*_, each sample was represented by features in IFS_*i*_ and the basic prediction engine was executed on all samples for classification and was evaluated by tenfold cross-validation [41].
(iii) Evaluate the performance of the basic prediction engine by calculating MCC and select features in the feature set that induces the highest MCC as the optimal features.

## 3. Results and Discussion

### 3.1. Results of mRMR

**Figure 1:**
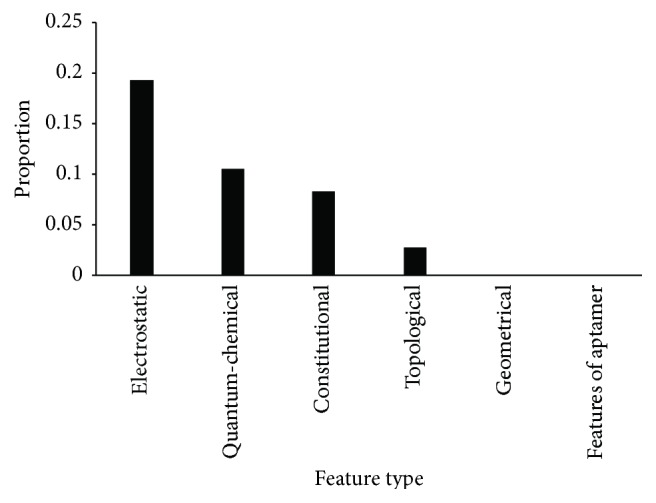
The proportion of features listed in the top 10% of the MaxRel features list in each feature type.

The investigated 477 interactions were represented by 321 features. The mRMR method was employed to analyze these features. As a result, we obtained two lists, the MaxRel features list and the mRMR features list, which are provided in Supplemental Material II. For the MaxRel features list, we investigated the top 10% of features, which were important for the determination of aptamer-compound interactions.  Table 2 gives the distribution of these features, from which we can see that no features of the aptamers were among the top 10% of features of the MaxRel features list. Furthermore, because the number of considered features in each feature type is different, only considering the number of features listed in the top 10% of the MaxRel features list for each feature type has its limitation. Thus, we computed the proportion of the number of features in the top 10% of the MaxRel features list and total number of features in each feature type, as illustrated in Figure 1. It can be observed from Table 2 and Figure 1 that features of electrostatic and quantum-chemical descriptors were more related to the determination of aptamer-compound interactions than other interactions.

### 3.2. Results of IFS

**Figure 2:**
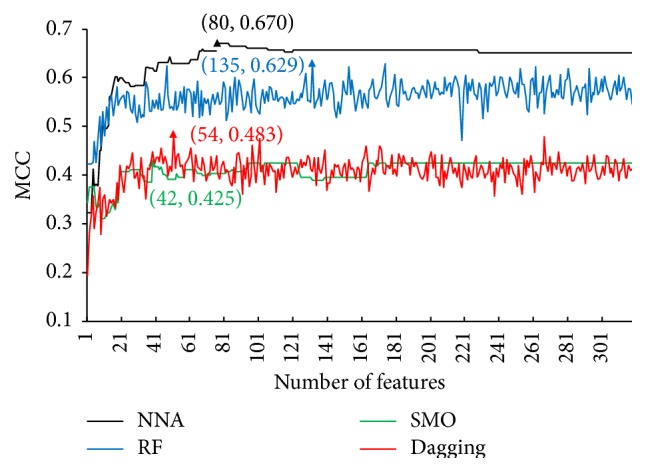
Four IFS curves plotted by taking MCC as the *y*-axis and the number of considered features as the *x*-axis for four basic prediction engines. The MCC values indicate the performance of various prediction models using different classifiers and different combination of features to represent interactions. It can be observed that using NNA as the classifier and the first 80 features in the mRMR features list to represent interactions can yield the best performance with the highest MCC value of 0.670.

By analyzing the MaxRel features list, we obtained only some important features that may play key roles in the determination of aptamer-compound interactions. On the other hand, an optimal prediction model based on a certain basic prediction engine can be built according to the mRMR features list and the IFS method. Following the procedures described in Section 2.6, a set of MCCs can be obtained using different numbers of features for each of the four basic prediction engines, which are listed in Supplemental Material III. For the readers' interest, the SNs, SPs and ACCs are also provided in Supplemental Material III. Based on the MCCs obtained by IFS method and four basic prediction engines, we plotted four curves, namely, IFS curves, for four basic prediction engines by setting MCC as the *y*-axis and the number of considered features (i.e., the subscript *i* of IFS_*i*_) as the *x*-axis.  Figure 2 shows these four curves, from which we can clearly observe that the maximum MCC for NNA, RF, SMO, and Dagging was 0.670, 0.629, 0.425, and 0.483, respectively, when the first 80, 135, 42, and 54 features in the mRMR features list were used. Thus, the NNA yielded the best performance (MCC 0.670) using the first 80 features in the mRMR features list. For readers' interest, the SN, SP, and ACC obtained using the NNA and first 80 features in the mRMR feature lists were 0.780, 0.890, and 0.853, respectively. It can be observed that the performance of the NNA is much better than the performances of SMO and Dagging. The possible reason is that the current data of aptamer-compound interactions is so complicated that its distribution is not clear, inducing difficulties for making prediction by the kernel function methods (e.g., SMO) or boosting methods (e.g., Dagging), while the NNA is good at dealing with this type of data. The IFS results of NNA suggest that the first 80 features in the mRMR feature lists were the optimal features to identify aptamer-compound interactions. The prediction model based on the NNA and 80 optimal features was the optimal prediction model. The following section gives a detailed discussion of the 88 features used in the optimal prediction model.

### 3.3. Prediction Results of Some Specific Examples

According to the results mentioned in Section 3.2, the optimal prediction model used the NNA as the classifier and the 80 optimal features to represent aptamer-compound interactions. To provide more clues for other investigators to study aptamer-compound interactions, we listed the predicted results of 477 interactions in Supplemental Material IV. Because the SN obtained by the optimal prediction model was 0.78, meaning that 124 of 159 aptamer-compound interactions were correctly predicted, five such examples are listed in first five rows in Table 3. For the negative interactions, those that were predicted to be “positive” were more important than others because they may be potential true aptamer-compound interactions. The last five rows of Table 3 list such five negative interactions.

### 3.4. Analysis of the Optimal Features

**Figure 3:**
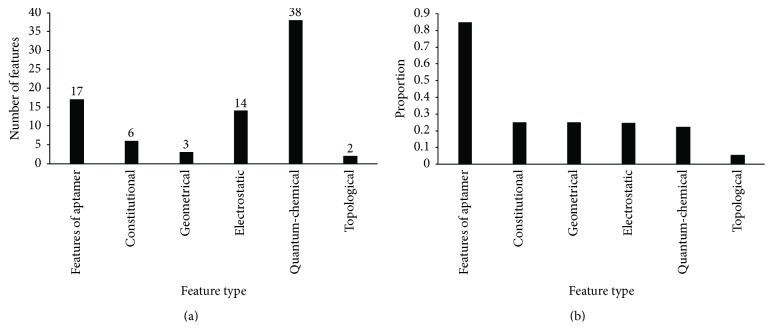
(a) The distribution of the 80 optimal features. (b) The proportion of features among the 80 optimal features in each feature type.

The 80 optimal features can be categorized into six types, including features of aptamer, constitutional, electrostatic, geometrical, quantum-chemical, and topological features. The distributions of these six feature types are illustrated in Figure 3(a). Like the analysis of the top 10% features in the MaxRel features list, we also calculated the proportion of the number of features among the 80 optimal features and the total number of features in each feature type, as illustrated in Figure 3(b).

The quantum-chemical features make up approximately 50% of 80 optimal features. Among these features, the tot dipole moment of the target molecule seems to be statistically essential for aptamer-target interactions, represents specific polarity characteristics, and, to some extent, reflects the space conformation of the target molecule [42, 43]. This finding is consistent with those of previous studies that show that the space conformation of the targets plays an important role in interactions with aptamers [44–46]. Moreover, quantum-chemical features also contain the characteristics of the total surface area and surface functional groups that may participate in the reaction. These characteristics make up the structural foundation of aptamer-compound binding [47]. Furthermore, the selected quantum-chemical features also describe conformational changes and atomic reactivity during the interaction. These traits explain aptamers' target specificity and why an aptamer can easily detect changes in a target's molecule structure [48, 49]. The results above suggest that our aptamer prediction has to include consideration of the molecular polarity and the surface electrostatic charge distribution of the target molecules. Consequently, prediction using the optimal prediction model might be widely implemented in the design of aptamers.

The electrostatic features were also a part of the optimal features. These traits reflect the distribution of the specific molecule surface charge. Molecule-molecule interaction (such as aptamer-target) is largely dependent on the interaction of respective charge [50, 51]. Such surface charge distribution is sure to have a correlation with aptamer-target interaction. Indeed, the polarity of targets as well as aptamers can induce aptamers to recognize their specific targets [52]. The distribution has also been demonstrated to be involved in aptamer-protein interactions. A typical example is the TBA (thrombin binding aptamer) [53]. Similarly, polarity may also play a crucial role in aptamer-compound interactions.

Constitutional features also play a unique role in the interaction. Certain features may combine to act as a standard to distinguish the material categories. Apart from characteristics describing the target compounds, aptamer frequency (the composition of nucleotide and dual nucleotide) can also interfere with the reaction by remodeling the spatial conformation of the aptamers. A stable and target-specific spatial conformation is the foundation of the aptamers' function [54–56]. Considering that the conformation of nucleic acid is mainly based on interactions between nucleotides, the composition of nucleotides and dual nucleotides may influence aptamers' specific three-dimensional structures and their stability. Moreover, some specific compounds may have the ability to recognize nucleotide chains, which may contain a characteristic nucleotide frequency. Those compounds interact with aptamers based on sequence specificity [57, 58]. Our results further confirm that the polar properties and distribution of molecular surface charge and aptamer frequency are significant for the interaction between the aptamers and their respective targets.

All in all, our prediction of proper aptamers against compounds depends on the traits of polarity, surface charge distribution of the compounds, constitutional features, and aptamer frequency. Our prediction using the mRMR program depends on the propensities of the compounds and the nucleotide (dual nucleotide) frequency of aptamers. In conclusion, in addition to protein analysis, mRMR can also be applied to design matching aptamers to specifically identify objective compounds.

## 4. Conclusions

Our study analyzed and identified the important features that influence the matching of aptamers to compounds. Maximum Relevance Minimum Redundancy and incremental feature selection were performed on a dataset, in which compounds and aptamers were represented by descriptors and nucleotide compositions, respectively. As a result, some key features were extracted and an optimal prediction model was built based on the nearest neighbor algorithm. The novel findings of our study may give new insights into the investigation of aptamer-compound interactions.

## Supplementary Material

The Supplementary Material contains four files. In detail, the Supplementary Material I lists 159 positive interactions and 318 negative interactions; the Supplementary Material II lists MaxRel features list and mRMR features list; Supplementary Material III lists the SNs, SPs, ACCs and MCCs obtained by IFS and four basic prediction engines; Supplementary Material IV lists predicted results of all interactions obtained by the optimal prediction model.

## Figures and Tables

**Table 1:** Distribution of the features investigated in this study.

Feature type	Number of features
Features of aptamer	
Frequency of single nucleotide	4
Frequency of dinucleotide	16
Features of compound	
Constitutional	24
Electrostatic	57
Geometrical	12
Quantum-chemical	171
Topological	37
Total	321

**Table 2:** Distribution of the top 10% features in the MaxRel features list.

Feature type	Number of features	Feature names
Features of aptamer	0	—

Constitutional	2	Number of double bonds; number of O atoms

Electrostatic	11	DPSA-1 difference in CPSAs (PPSA1-PNSA1) [Zefirov's PC]; HA dependent HDCA-2 [Zefirov's PC]; Max partial charge for H atom [Zefirov's PC]; PNSA-3 atomic charge weighted PNSA [Zefirov's PC]; HACA-2 [Zefirov's PC]; HACA-1 [Zefirov's PC]; min(#HA_#HD) [Zefirov's PC]; count of H-acceptor sites [Zefirov's PC]; HA dependent HDSA-1/TMSA [Zefirov's PC]; DPSA-3 difference in CPSAs (PPSA3-PNSA3) [Zefirov's PC]; HA dependent HDCA-1 [Zefirov's PC]

Geometrical	0	—

Quantum-chemical	18	Tot dipole of the molecule; tot point-charge comp. of the molecular dipole; ESP-HA dependent HDSA-2 [quantum-chemical PC]; ESP-HA dependent HDCA-2 [quantum-chemical PC]; ESP-HACA-2 [quantum-chemical PC]; HA dependent HDSA-2 [quantum-chemical PC]; final heat of formation; ESP-Max net atomic charge for H atom; ESP-DPSA-1 difference in CPSAs (PPSA1-PNSA1) [quantum-chemical PC]; HA dependent HDCA-2 [quantum-chemical PC]; HOMO - LUMO energy gap; ESP-HA dependent HDSA-1 [quantum-chemical PC]; min(#HA_#HD) [quantum-chemical PC]; ESP-count of H-acceptor sites [quantum-chemical PC]; ESP-min(#HA_#HD) [quantum-chemical PC]; count of H-acceptor sites [quantum-chemical PC]; DPSA-1 difference in CPSAs (PPSA1-PNSA1) [quantum-chemical PC]; HA dependent HDCA-1 [quantum-chemical PC]

Topological	1	Average structural information content (order 1)

**Table 3:** Predicted results of some specific examples obtained by the optimal prediction model.

Compound	Aptamer	Predicted class	True class
Arsenate	20000526-arsenic-5	Positive	Positive
Isoleucine	15772067-isoleucine-1	Positive	Positive
Dopamine	9245404-dopamine-4	Positive	Positive
Chitin	10743940-chitin-5	Positive	Positive
N-Acetylneuraminic acid	23042406-Neu5Ac-1	Positive	Positive
Isoleucine	14980623-sialyllactose-1	Positive	Negative
Dopamine	18983163-ochratoxin A-3	Positive	Negative
Chitin	10786843-L tyrosine-3	Positive	Negative
Tyrosine	20000526-arsenic-Ma-1	Positive	Negative
N-Acetylneuraminic acid	21076782-L-tryptophan-1	Positive	Negative
