# Supplementary material for: Analysis and Identification of Aptamer-Compound Interactions with a Maximum Relevance Minimum Redundancy and Nearest Neighbor Algorithm
Source: Biomed Res Int. 2016 Feb 3;2016:8351204. doi: 10.1155/2016/8351204 (PMC4756144; doi:10.1155/2016/8351204)
Supplement: Supplementary file 1 — The Supplementary Material contains four files. In detail, the Supplementary Material I lists 159 positive interactions and 318 negative interactions; the Supplementary Material II lists MaxRel features list and mRMR features list; Supplementary Material III lists the SNs, SPs, ACCs and MCCs obtained by IFS and four basic prediction engines; Supplementary Material IV lists predicted results of all interactions obtained by the optimal prediction model. [file 8351204.f1.zip › Supp-I.docx]

**Supplemental Material I.** 159 positive interactions and 318 negative interactions

| **Positive or negative** | **Compound** | **Aptamper** |
| --- | --- | --- |
| Positive | Cyclic adeNosiNe moNoPhosPhate | 10913311-cAMP-3 |
| Positive | Cyclic adeNosiNe moNoPhosPhate | 10913311-cAMP-2 |
| Positive | Cyclic adeNosiNe moNoPhosPhate | 10913311-cAMP-4 |
| Positive | Cyclic adeNosiNe moNoPhosPhate | 10913311-cAMP-1 |
| Positive | OchratoxiN A | 18983163-ochratoxiN A-Ma-13 |
| Positive | SulforhodamiNe B | 9831529-sulforhodamiNe B-1 |
| Positive | SulforhodamiNe B | 9831529-sulforhodamiNe B-Ma-16 |
| Positive | SulforhodamiNe B | 9831529-sulforhodamiNe B-Ma-10 |
| Positive | SulforhodamiNe B | 9831529-sulforhodamiNe B-Ma-15 |
| Positive | SulforhodamiNe B | 9831529-sulforhodamiNe B-Ma-3 |
| Positive | SulforhodamiNe B | 9831529-sulforhodamiNe B-Ma-11 |
| Positive | SulforhodamiNe B | 9831529-sulforhodamiNe B-Ma-14 |
| Positive | SulforhodamiNe B | 9831529-sulforhodamiNe B-Ma-2 |
| Positive | SulforhodamiNe B | 9831529-sulforhodamiNe B-Ma-9 |
| Positive | SulforhodamiNe B | 9831529-sulforhodamiNe B-Ma-6 |
| Positive | SulforhodamiNe B | 9831529-sulforhodamiNe B-Ma-4 |
| Positive | SulforhodamiNe B | 9831529-sulforhodamiNe B-Ma-13 |
| Positive | SulforhodamiNe B | 9831529-sulforhodamiNe B-Ma-7 |
| Positive | SulforhodamiNe B | 9831529-sulforhodamiNe B-Ma-8 |
| Positive | SulforhodamiNe B | 9831529-sulforhodamiNe B-Ma-17 |
| Positive | SulforhodamiNe B | 9831529-sulforhodamiNe B-Ma-5 |
| Positive | SulforhodamiNe B | 9831529-sulforhodamiNe B-Ma-12 |
| Positive | SulforhodamiNe B | 9831529-sulforhodamiNe B-Ma-20 |
| Positive | SulforhodamiNe B | 9831529-sulforhodamiNe B-Ma-19 |
| Positive | SulforhodamiNe B | 9831529-sulforhodamiNe B-Ma-18 |
| Positive | SulforhodamiNe B | 9831529-sulforhodamiNe B-Ma-21 |
| Positive | OchratoxiN A | 18983163-ochratoxiN A-1 |
| Positive | OchratoxiN A | 18983163-ochratoxiN A-2 |
| Positive | OchratoxiN A | 18983163-ochratoxiN A-3 |
| Positive | OchratoxiN A | 18983163-ochratoxiN A-4 |
| Positive | OchratoxiN A | 18983163-ochratoxiN A-5 |
| Positive | OchratoxiN A | 18983163-ochratoxiN A-6 |
| Positive | OchratoxiN A | 18983163-ochratoxiN A-7 |
| Positive | OchratoxiN A | 18983163-ochratoxiN A-8 |
| Positive | OchratoxiN A | 18983163-ochratoxiN A-9 |
| Positive | OchratoxiN A | 18983163-ochratoxiN A-10 |
| Positive | OchratoxiN A | 18983163-ochratoxiN A-11 |
| Positive | OchratoxiN A | 18983163-ochratoxiN A-12 |
| Positive | OchratoxiN A | 18983163-ochratoxiN A-14 |
| Positive | OchratoxiN A | 18983163-ochratoxiN A-Ma-15 |
| Positive | OchratoxiN A | 18983163-ochratoxiN A--Ma-16 |
| Positive | OchratoxiN A | 18983163-ochratoxiN A-Ma-17 |
| Positive | OchratoxiN A | 18983163-ochratoxiN A-Ma-18 |
| Positive | OchratoxiN A | 18983163-ochratoxiN A-19 |
| Positive | OchratoxiN A | 18983163-ochratoxiN A-20 |
| Positive | OchratoxiN A | 18983163-ochratoxiN A-22 |
| Positive | OchratoxiN A | 18983163-ochratoxiN A-24 |
| Positive | OchratoxiN A | 18983163-ochratoxiN A-25 |
| Positive | OchratoxiN A | 18983163-ochratoxiN A-21 |
| Positive | OchratoxiN A | 18983163-ochratoxiN A-23 |
| Positive | DoPamiNe | 9245404-doPamiNe-1 |
| Positive | DoPamiNe | 9245404-doPamiNe-2 |
| Positive | DoPamiNe | 9245404-doPamiNe-Ma-1 |
| Positive | Cellobiose | 9576904-cellobiose-1 |
| Positive | Cellobiose | 9576904-cellobiose-3 |
| Positive | Cellobiose | 9576904-cellobiose-2 |
| Positive | HematoPorPhyriN IX | 11128644-HematoPorPhyriN-1 |
| Positive | HematoPorPhyriN IX | 11128644-HematoPorPhyriN-2 |
| Positive | HematoPorPhyriN IX | 11128644-HematoPorPhyriN-3 |
| Positive | HematoPorPhyriN IX | 11128644-HematoPorPhyriN-4 |
| Positive | HematoPorPhyriN IX | 11128644-HematoPorPhyriN-5 |
| Positive | HematoPorPhyriN IX | 11128644-HematoPorPhyriN-6 |
| Positive | HematoPorPhyriN IX | 11128644-HematoPorPhyriN-7 |
| Positive | HematoPorPhyriN IX | 11128644-HematoPorPhyriN-8 |
| Positive | HematoPorPhyriN IX | 11128644-HematoPorPhyriN-9 |
| Positive | HematoPorPhyriN IX | 11128644-HematoPorPhyriN-Ma-1 |
| Positive | HematoPorPhyriN IX | 11128644-HematoPorPhyriN-Ma-2 |
| Positive | DoPamiNe | 9245404-DoPamiNe-4 |
| Positive | DoPamiNe | 9245404-DoPamiNe-5 |
| Positive | DoPamiNe | 9245404-DoPamiNe-6 |
| Positive | DoPamiNe | 9245404-DoPamiNe-7 |
| Positive | DoPamiNe | 9245404-DoPamiNe-8 |
| Positive | DoPamiNe | 9245404-DoPamiNe-9 |
| Positive | SulforhodamiNe B | 9889155-sulforhodamiNe B-1 |
| Positive | L-ArgiNiNe | 8604334-LArgiNiNe-1 |
| Positive | S-AdeNosyl-L-homocysteiNe | 10852725-SAH-1 |
| Positive | S-AdeNosyl-L-homocysteiNe | 10852725-SAH-2 |
| Positive | AdeNosiNe triPhosPhate | 11101810-ATP-1 |
| Positive | CodeiNe | 17038331-codeiNe-1 |
| Positive | CodeiNe | 17038331-codeiNe-2 |
| Positive | CodeiNe | 17038331-codeiNe-3 |
| Positive | CodeiNe | 17038331-codeiNe-4 |
| Positive | CodeiNe | 17038331-codeiNe-5 |
| Positive | CodeiNe | 17038331-codeiNe-6 |
| Positive | CodeiNe | 17038331-codeiNe-7 |
| Positive | CodeiNe | 17038331-codeiNe-8 |
| Positive | CodeiNe | 17038331-codeiNe-9 |
| Positive | CodeiNe | 17038331-codeiNe-10 |
| Positive | CodeiNe | 17038331-codeiNe-11 |
| Positive | CodeiNe | 17038331-codeiNe-12 |
| Positive | CodeiNe | 17038331-codeiNe-13 |
| Positive | CodeiNe | 17038331-codeiNe-14 |
| Positive | CodeiNe | 17038331-codeiNe-15 |
| Positive | CodeiNe | 17038331-codeiNe-16 |
| Positive | CodeiNe | 17038331-codeiNe-Ma-1 |
| Positive | CodeiNe | 17038331-codeiNe-19 |
| Positive | CodeiNe | 17038331-codeiNe-17 |
| Positive | CodeiNe | 17038331-codeiNe-20 |
| Positive | CodeiNe | 17038331-codeiNe-Ma-2 |
| Positive | CodeiNe | 17038331-codeiNe-21 |
| Positive | CodeiNe | 17038331-codeiNe-18 |
| Positive | TyrosiNe | 10786843-L TyrosiNe-1 |
| Positive | TyrosiNe | 10786843-L TyrosiNe-2 |
| Positive | TyrosiNe | 10786843-L TyrosiNe-3 |
| Positive | SulforhodamiNe B | 9889155-sulforhodamiNe B-2 |
| Positive | SulforhodamiNe B | 9889155-sulforhodamiNe B-Ma-1 |
| Positive | ChitiN | 10743940-chitiN-1 |
| Positive | ChitiN | 10743940-chitiN-2 |
| Positive | ChitiN | 10743940-chitiN-3 |
| Positive | ChitiN | 10743940-chitiN-4 |
| Positive | ChitiN | 10743940-chitiN-5 |
| Positive | ChitiN | 10743940-chitiN-6 |
| Positive | ChitiN | 10743940-chitiN-7 |
| Positive | Malachite greeN | 10339553-MalachiteGreeN-1 |
| Positive | IsoleuciNe | 15772067-isoleuciNe-1 |
| Positive | IsoleuciNe | 14561881A-isoleuciNe-3 |
| Positive | IsoleuciNe | 14561881B-isoleuciNe-3 |
| Positive | IsoleuciNe | 14561881B-isoleuciNe-4 |
| Positive | Sialyllactose | 14980623-sialyllactose-1 |
| Positive | FADH | 11851395-FAD-1 |
| Positive | AdeNiNe | 11705996-adeNiNe-1 |
| Positive | AdeNiNe | 11705996-adeNiNe-Ma-1 |
| Positive | AdeNosiNe triPhosPhate | 12873145-ATP-1 |
| Positive | FumoNisiN B1 | 21614178-fumoNisiN B1-1 |
| Positive | TryPtoPhaN | 21076782-L-TryPtoPhaN-1 |
| Positive | TryPtoPhaN | 21076782-L-TryPtoPhaN-Ma-1 |
| Positive | ThyroxiNe | 17163839-ThyroxiNe-Ma-1 |
| Positive | AcetamiPrid | 21306108-AcetamiPrid-1 |
| Positive | BisPheNol A | 21413891-BisPheNol A-1 |
| Positive | Phorate | 22261866-Pesticides-1 |
| Positive | Omethoate | 22261866-Pesticides-1 |
| Positive | Phorate | 22261866-Pesticides-2 |
| Positive | Omethoate | 22261866-Pesticides-2 |
| Positive | ArseNate | 20000526-arseNic-1 |
| Positive | ArseNate | 20000526-arseNic-2 |
| Positive | ArseNate | 20000526-arseNic-3 |
| Positive | ArseNate | 20000526-arseNic-4 |
| Positive | ArseNate | 20000526-arseNic-5 |
| Positive | ArseNate | 20000526-arseNic-6 |
| Positive | ArseNate | 20000526-arseNic-7 |
| Positive | ArseNate | 20000526-arseNic-8 |
| Positive | ArseNate | 20000526-arseNic-Ma-1 |
| Positive | Glutamic acid | 18187867-Glu-2 |
| Positive | N-AcetylNeuramiNic acid | 23042406-Neu5Ac-1 |
| Positive | N-GlycolylNeuramiNic acid | 23042406-Neu5Ac-1 |
| Positive | N-AcetylNeuramiNic acid | 23042406-Neu5Ac-Ma-1 |
| Positive | N-AcetylNeuramiNic acid | 23042406-Neu5Ac-2 |
| Positive | N-AcetylNeuramiNic acid | 23042406-Neu5Ac-3 |
| Positive | N-AcetylNeuramiNic acid | 23042406-Neu5Ac-4 |
| Positive | N-AcetylNeuramiNic acid | 23042406-Neu5Ac-5 |
| Positive | N-AcetylNeuramiNic acid | 23042406-Neu5Ac-6 |
| Positive | N-AcetylNeuramiNic acid | 23042406-Neu5Ac-7 |
| Positive | Cholic acid | 10978501-cholic acid-1 |
| Positive | Cholic acid | 10978501-cholic acid-2 |
| Positive | Cholic acid | 10978501-cholic acid-3 |
| Positive | Cholic acid | 10978501-cholic acid-4 |
| Positive | Cholic acid | 10978501-cholic acid-5 |
| Positive | Cholic acid | 10978501-cholic acid-6 |
| Positive | Cholic acid | 10978501-cholic acid-Ma-1 |
| Negative | FADH | 10913311-cAMP-3 |
| Negative | DoPamiNe | 10913311-cAMP-3 |
| Negative | TyrosiNe | 10913311-cAMP-2 |
| Negative | L-ArgiNiNe | 10913311-cAMP-2 |
| Negative | L-ArgiNiNe | 10913311-cAMP-4 |
| Negative | ArseNate | 10913311-cAMP-4 |
| Negative | Sialyllactose | 10913311-cAMP-1 |
| Negative | Cellobiose | 10913311-cAMP-1 |
| Negative | S-AdeNosyl-L-homocysteiNe | 18983163-ochratoxiN A-Ma-13 |
| Negative | Sialyllactose | 18983163-ochratoxiN A-Ma-13 |
| Negative | CodeiNe | 9831529-sulforhodamiNe B-1 |
| Negative | BisPheNol A | 9831529-sulforhodamiNe B-1 |
| Negative | ChitiN | 9831529-sulforhodamiNe B-Ma-16 |
| Negative | L-ArgiNiNe | 9831529-sulforhodamiNe B-Ma-16 |
| Negative | Sialyllactose | 9831529-sulforhodamiNe B-Ma-10 |
| Negative | L-ArgiNiNe | 9831529-sulforhodamiNe B-Ma-10 |
| Negative | Glutamic acid | 9831529-sulforhodamiNe B-Ma-15 |
| Negative | Sialyllactose | 9831529-sulforhodamiNe B-Ma-15 |
| Negative | HematoPorPhyriN IX | 9831529-sulforhodamiNe B-Ma-3 |
| Negative | Cellobiose | 9831529-sulforhodamiNe B-Ma-3 |
| Negative | TryPtoPhaN | 9831529-sulforhodamiNe B-Ma-11 |
| Negative | L-ArgiNiNe | 9831529-sulforhodamiNe B-Ma-11 |
| Negative | S-AdeNosyl-L-homocysteiNe | 9831529-sulforhodamiNe B-Ma-14 |
| Negative | TryPtoPhaN | 9831529-sulforhodamiNe B-Ma-14 |
| Negative | N-GlycolylNeuramiNic acid | 9831529-sulforhodamiNe B-Ma-2 |
| Negative | HematoPorPhyriN IX | 9831529-sulforhodamiNe B-Ma-2 |
| Negative | ArseNate | 9831529-sulforhodamiNe B-Ma-9 |
| Negative | N-GlycolylNeuramiNic acid | 9831529-sulforhodamiNe B-Ma-9 |
| Negative | S-AdeNosyl-L-homocysteiNe | 9831529-sulforhodamiNe B-Ma-6 |
| Negative | Glutamic acid | 9831529-sulforhodamiNe B-Ma-6 |
| Negative | AdeNosiNe triPhosPhate | 9831529-sulforhodamiNe B-Ma-4 |
| Negative | Cellobiose | 9831529-sulforhodamiNe B-Ma-4 |
| Negative | Omethoate | 9831529-sulforhodamiNe B-Ma-13 |
| Negative | N-GlycolylNeuramiNic acid | 9831529-sulforhodamiNe B-Ma-13 |
| Negative | Glutamic acid | 9831529-sulforhodamiNe B-Ma-7 |
| Negative | Malachite greeN | 9831529-sulforhodamiNe B-Ma-7 |
| Negative | S-AdeNosyl-L-homocysteiNe | 9831529-sulforhodamiNe B-Ma-8 |
| Negative | Phorate | 9831529-sulforhodamiNe B-Ma-8 |
| Negative | HematoPorPhyriN IX | 9831529-sulforhodamiNe B-Ma-17 |
| Negative | DoPamiNe | 9831529-sulforhodamiNe B-Ma-17 |
| Negative | L-ArgiNiNe | 9831529-sulforhodamiNe B-Ma-5 |
| Negative | ArseNate | 9831529-sulforhodamiNe B-Ma-5 |
| Negative | N-AcetylNeuramiNic acid | 9831529-sulforhodamiNe B-Ma-12 |
| Negative | DoPamiNe | 9831529-sulforhodamiNe B-Ma-12 |
| Negative | TryPtoPhaN | 9831529-sulforhodamiNe B-Ma-20 |
| Negative | TyrosiNe | 9831529-sulforhodamiNe B-Ma-20 |
| Negative | TyrosiNe | 9831529-sulforhodamiNe B-Ma-19 |
| Negative | FADH | 9831529-sulforhodamiNe B-Ma-19 |
| Negative | Malachite greeN | 9831529-sulforhodamiNe B-Ma-18 |
| Negative | N-GlycolylNeuramiNic acid | 9831529-sulforhodamiNe B-Ma-18 |
| Negative | Cyclic adeNosiNe moNoPhosPhate | 9831529-sulforhodamiNe B-Ma-21 |
| Negative | FumoNisiN B1 | 9831529-sulforhodamiNe B-Ma-21 |
| Negative | CodeiNe | 18983163-ochratoxiN A-1 |
| Negative | AdeNiNe | 18983163-ochratoxiN A-1 |
| Negative | ChitiN | 18983163-ochratoxiN A-2 |
| Negative | L-ArgiNiNe | 18983163-ochratoxiN A-2 |
| Negative | TyrosiNe | 18983163-ochratoxiN A-3 |
| Negative | DoPamiNe | 18983163-ochratoxiN A-3 |
| Negative | Omethoate | 18983163-ochratoxiN A-4 |
| Negative | Glutamic acid | 18983163-ochratoxiN A-4 |
| Negative | TryPtoPhaN | 18983163-ochratoxiN A-5 |
| Negative | ArseNate | 18983163-ochratoxiN A-5 |
| Negative | AcetamiPrid | 18983163-ochratoxiN A-6 |
| Negative | SulforhodamiNe B | 18983163-ochratoxiN A-6 |
| Negative | IsoleuciNe | 18983163-ochratoxiN A-7 |
| Negative | Cyclic adeNosiNe moNoPhosPhate | 18983163-ochratoxiN A-7 |
| Negative | ArseNate | 18983163-ochratoxiN A-8 |
| Negative | Phorate | 18983163-ochratoxiN A-8 |
| Negative | SulforhodamiNe B | 18983163-ochratoxiN A-9 |
| Negative | ThyroxiNe | 18983163-ochratoxiN A-9 |
| Negative | TyrosiNe | 18983163-ochratoxiN A-10 |
| Negative | CodeiNe | 18983163-ochratoxiN A-10 |
| Negative | AcetamiPrid | 18983163-ochratoxiN A-11 |
| Negative | CodeiNe | 18983163-ochratoxiN A-11 |
| Negative | CodeiNe | 18983163-ochratoxiN A-12 |
| Negative | AcetamiPrid | 18983163-ochratoxiN A-12 |
| Negative | ChitiN | 18983163-ochratoxiN A-14 |
| Negative | CodeiNe | 18983163-ochratoxiN A-14 |
| Negative | FADH | 18983163-ochratoxiN A-Ma-15 |
| Negative | HematoPorPhyriN IX | 18983163-ochratoxiN A-Ma-15 |
| Negative | S-AdeNosyl-L-homocysteiNe | 18983163-ochratoxiN A--Ma-16 |
| Negative | ChitiN | 18983163-ochratoxiN A--Ma-16 |
| Negative | HematoPorPhyriN IX | 18983163-ochratoxiN A-Ma-17 |
| Negative | S-AdeNosyl-L-homocysteiNe | 18983163-ochratoxiN A-Ma-17 |
| Negative | AdeNosiNe triPhosPhate | 18983163-ochratoxiN A-Ma-18 |
| Negative | Malachite greeN | 18983163-ochratoxiN A-Ma-18 |
| Negative | AdeNiNe | 18983163-ochratoxiN A-19 |
| Negative | IsoleuciNe | 18983163-ochratoxiN A-19 |
| Negative | Cellobiose | 18983163-ochratoxiN A-20 |
| Negative | Phorate | 18983163-ochratoxiN A-20 |
| Negative | N-GlycolylNeuramiNic acid | 18983163-ochratoxiN A-22 |
| Negative | SulforhodamiNe B | 18983163-ochratoxiN A-22 |
| Negative | Malachite greeN | 18983163-ochratoxiN A-24 |
| Negative | Omethoate | 18983163-ochratoxiN A-24 |
| Negative | FumoNisiN B1 | 18983163-ochratoxiN A-25 |
| Negative | Omethoate | 18983163-ochratoxiN A-25 |
| Negative | S-AdeNosyl-L-homocysteiNe | 18983163-ochratoxiN A-21 |
| Negative | Omethoate | 18983163-ochratoxiN A-21 |
| Negative | AdeNosiNe triPhosPhate | 18983163-ochratoxiN A-23 |
| Negative | Omethoate | 18983163-ochratoxiN A-23 |
| Negative | ArseNate | 9245404-doPamiNe-1 |
| Negative | Sialyllactose | 9245404-doPamiNe-1 |
| Negative | Malachite greeN | 9245404-doPamiNe-2 |
| Negative | FumoNisiN B1 | 9245404-doPamiNe-2 |
| Negative | ChitiN | 9245404-doPamiNe-Ma-1 |
| Negative | AcetamiPrid | 9245404-doPamiNe-Ma-1 |
| Negative | Phorate | 9576904-cellobiose-1 |
| Negative | AdeNiNe | 9576904-cellobiose-1 |
| Negative | S-AdeNosyl-L-homocysteiNe | 9576904-cellobiose-3 |
| Negative | Cholic acid | 9576904-cellobiose-3 |
| Negative | ThyroxiNe | 9576904-cellobiose-2 |
| Negative | AdeNosiNe triPhosPhate | 9576904-cellobiose-2 |
| Negative | Sialyllactose | 11128644-HematoPorPhyriN-1 |
| Negative | N-GlycolylNeuramiNic acid | 11128644-HematoPorPhyriN-1 |
| Negative | DoPamiNe | 11128644-HematoPorPhyriN-2 |
| Negative | OchratoxiN A | 11128644-HematoPorPhyriN-2 |
| Negative | ThyroxiNe | 11128644-HematoPorPhyriN-3 |
| Negative | Glutamic acid | 11128644-HematoPorPhyriN-3 |
| Negative | Malachite greeN | 11128644-HematoPorPhyriN-4 |
| Negative | Phorate | 11128644-HematoPorPhyriN-4 |
| Negative | AdeNosiNe triPhosPhate | 11128644-HematoPorPhyriN-5 |
| Negative | Sialyllactose | 11128644-HematoPorPhyriN-5 |
| Negative | Cellobiose | 11128644-HematoPorPhyriN-6 |
| Negative | AdeNiNe | 11128644-HematoPorPhyriN-6 |
| Negative | Glutamic acid | 11128644-HematoPorPhyriN-7 |
| Negative | Cellobiose | 11128644-HematoPorPhyriN-7 |
| Negative | Cellobiose | 11128644-HematoPorPhyriN-8 |
| Negative | Cyclic adeNosiNe moNoPhosPhate | 11128644-HematoPorPhyriN-8 |
| Negative | ArseNate | 11128644-HematoPorPhyriN-9 |
| Negative | Sialyllactose | 11128644-HematoPorPhyriN-9 |
| Negative | Phorate | 11128644-HematoPorPhyriN-Ma-1 |
| Negative | Omethoate | 11128644-HematoPorPhyriN-Ma-1 |
| Negative | L-ArgiNiNe | 11128644-HematoPorPhyriN-Ma-2 |
| Negative | ThyroxiNe | 11128644-HematoPorPhyriN-Ma-2 |
| Negative | AcetamiPrid | 9245404-DoPamiNe-4 |
| Negative | ChitiN | 9245404-DoPamiNe-4 |
| Negative | N-AcetylNeuramiNic acid | 9245404-DoPamiNe-5 |
| Negative | Omethoate | 9245404-DoPamiNe-5 |
| Negative | TyrosiNe | 9245404-DoPamiNe-6 |
| Negative | ThyroxiNe | 9245404-DoPamiNe-6 |
| Negative | FADH | 9245404-DoPamiNe-7 |
| Negative | Phorate | 9245404-DoPamiNe-7 |
| Negative | Cyclic adeNosiNe moNoPhosPhate | 9245404-DoPamiNe-8 |
| Negative | Phorate | 9245404-DoPamiNe-8 |
| Negative | CodeiNe | 9245404-DoPamiNe-9 |
| Negative | Malachite greeN | 9245404-DoPamiNe-9 |
| Negative | Phorate | 9889155-sulforhodamiNe B-1 |
| Negative | S-AdeNosyl-L-homocysteiNe | 9889155-sulforhodamiNe B-1 |
| Negative | ChitiN | 8604334-LArgiNiNe-1 |
| Negative | ThyroxiNe | 8604334-LArgiNiNe-1 |
| Negative | ChitiN | 10852725-SAH-1 |
| Negative | BisPheNol A | 10852725-SAH-1 |
| Negative | ArseNate | 10852725-SAH-2 |
| Negative | Sialyllactose | 10852725-SAH-2 |
| Negative | Omethoate | 11101810-ATP-1 |
| Negative | TryPtoPhaN | 11101810-ATP-1 |
| Negative | Sialyllactose | 17038331-codeiNe-1 |
| Negative | ChitiN | 17038331-codeiNe-1 |
| Negative | N-GlycolylNeuramiNic acid | 17038331-codeiNe-2 |
| Negative | ThyroxiNe | 17038331-codeiNe-2 |
| Negative | AdeNosiNe triPhosPhate | 17038331-codeiNe-3 |
| Negative | S-AdeNosyl-L-homocysteiNe | 17038331-codeiNe-3 |
| Negative | HematoPorPhyriN IX | 17038331-codeiNe-4 |
| Negative | Malachite greeN | 17038331-codeiNe-4 |
| Negative | Cellobiose | 17038331-codeiNe-5 |
| Negative | ChitiN | 17038331-codeiNe-5 |
| Negative | Cyclic adeNosiNe moNoPhosPhate | 17038331-codeiNe-6 |
| Negative | Glutamic acid | 17038331-codeiNe-6 |
| Negative | Phorate | 17038331-codeiNe-7 |
| Negative | ArseNate | 17038331-codeiNe-7 |
| Negative | HematoPorPhyriN IX | 17038331-codeiNe-8 |
| Negative | AcetamiPrid | 17038331-codeiNe-8 |
| Negative | N-GlycolylNeuramiNic acid | 17038331-codeiNe-9 |
| Negative | Cellobiose | 17038331-codeiNe-9 |
| Negative | N-AcetylNeuramiNic acid | 17038331-codeiNe-10 |
| Negative | AcetamiPrid | 17038331-codeiNe-10 |
| Negative | Omethoate | 17038331-codeiNe-11 |
| Negative | TyrosiNe | 17038331-codeiNe-11 |
| Negative | ChitiN | 17038331-codeiNe-12 |
| Negative | OchratoxiN A | 17038331-codeiNe-12 |
| Negative | HematoPorPhyriN IX | 17038331-codeiNe-13 |
| Negative | S-AdeNosyl-L-homocysteiNe | 17038331-codeiNe-13 |
| Negative | Omethoate | 17038331-codeiNe-14 |
| Negative | S-AdeNosyl-L-homocysteiNe | 17038331-codeiNe-14 |
| Negative | AcetamiPrid | 17038331-codeiNe-15 |
| Negative | S-AdeNosyl-L-homocysteiNe | 17038331-codeiNe-15 |
| Negative | L-ArgiNiNe | 17038331-codeiNe-16 |
| Negative | BisPheNol A | 17038331-codeiNe-16 |
| Negative | AdeNosiNe triPhosPhate | 17038331-codeiNe-Ma-1 |
| Negative | Cyclic adeNosiNe moNoPhosPhate | 17038331-codeiNe-Ma-1 |
| Negative | TyrosiNe | 17038331-codeiNe-19 |
| Negative | N-GlycolylNeuramiNic acid | 17038331-codeiNe-19 |
| Negative | DoPamiNe | 17038331-codeiNe-17 |
| Negative | TyrosiNe | 17038331-codeiNe-17 |
| Negative | TyrosiNe | 17038331-codeiNe-20 |
| Negative | Sialyllactose | 17038331-codeiNe-20 |
| Negative | Sialyllactose | 17038331-codeiNe-Ma-2 |
| Negative | S-AdeNosyl-L-homocysteiNe | 17038331-codeiNe-Ma-2 |
| Negative | FADH | 17038331-codeiNe-21 |
| Negative | ThyroxiNe | 17038331-codeiNe-21 |
| Negative | TyrosiNe | 17038331-codeiNe-18 |
| Negative | AdeNosiNe triPhosPhate | 17038331-codeiNe-18 |
| Negative | IsoleuciNe | 10786843-L TyrosiNe-1 |
| Negative | Sialyllactose | 10786843-L TyrosiNe-1 |
| Negative | Phorate | 10786843-L TyrosiNe-2 |
| Negative | BisPheNol A | 10786843-L TyrosiNe-2 |
| Negative | IsoleuciNe | 10786843-L TyrosiNe-3 |
| Negative | ChitiN | 10786843-L TyrosiNe-3 |
| Negative | AdeNiNe | 9889155-sulforhodamiNe B-2 |
| Negative | FumoNisiN B1 | 9889155-sulforhodamiNe B-2 |
| Negative | ArseNate | 9889155-sulforhodamiNe B-Ma-1 |
| Negative | BisPheNol A | 9889155-sulforhodamiNe B-Ma-1 |
| Negative | AcetamiPrid | 10743940-chitiN-1 |
| Negative | S-AdeNosyl-L-homocysteiNe | 10743940-chitiN-1 |
| Negative | OchratoxiN A | 10743940-chitiN-2 |
| Negative | DoPamiNe | 10743940-chitiN-2 |
| Negative | Phorate | 10743940-chitiN-3 |
| Negative | Cholic acid | 10743940-chitiN-3 |
| Negative | S-AdeNosyl-L-homocysteiNe | 10743940-chitiN-4 |
| Negative | HematoPorPhyriN IX | 10743940-chitiN-4 |
| Negative | TyrosiNe | 10743940-chitiN-5 |
| Negative | OchratoxiN A | 10743940-chitiN-5 |
| Negative | TyrosiNe | 10743940-chitiN-6 |
| Negative | Omethoate | 10743940-chitiN-6 |
| Negative | L-ArgiNiNe | 10743940-chitiN-7 |
| Negative | AdeNiNe | 10743940-chitiN-7 |
| Negative | Cholic acid | 10339553-MalachiteGreeN-1 |
| Negative | Cellobiose | 10339553-MalachiteGreeN-1 |
| Negative | Sialyllactose | 15772067-isoleuciNe-1 |
| Negative | Malachite greeN | 15772067-isoleuciNe-1 |
| Negative | CodeiNe | 14561881A-isoleuciNe-3 |
| Negative | AdeNosiNe triPhosPhate | 14561881A-isoleuciNe-3 |
| Negative | ThyroxiNe | 14561881B-isoleuciNe-3 |
| Negative | ChitiN | 14561881B-isoleuciNe-3 |
| Negative | BisPheNol A | 14561881B-isoleuciNe-4 |
| Negative | N-GlycolylNeuramiNic acid | 14561881B-isoleuciNe-4 |
| Negative | TryPtoPhaN | 14980623-sialyllactose-1 |
| Negative | IsoleuciNe | 14980623-sialyllactose-1 |
| Negative | TyrosiNe | 11851395-FAD-1 |
| Negative | ArseNate | 11851395-FAD-1 |
| Negative | S-AdeNosyl-L-homocysteiNe | 11705996-adeNiNe-1 |
| Negative | Cellobiose | 11705996-adeNiNe-1 |
| Negative | S-AdeNosyl-L-homocysteiNe | 11705996-adeNiNe-Ma-1 |
| Negative | Cellobiose | 11705996-adeNiNe-Ma-1 |
| Negative | DoPamiNe | 12873145-ATP-1 |
| Negative | AdeNiNe | 12873145-ATP-1 |
| Negative | OchratoxiN A | 21614178-fumoNisiN B1-1 |
| Negative | CodeiNe | 21614178-fumoNisiN B1-1 |
| Negative | N-AcetylNeuramiNic acid | 21076782-L-TryPtoPhaN-1 |
| Negative | Phorate | 21076782-L-TryPtoPhaN-1 |
| Negative | OchratoxiN A | 21076782-L-TryPtoPhaN-Ma-1 |
| Negative | AdeNiNe | 21076782-L-TryPtoPhaN-Ma-1 |
| Negative | FumoNisiN B1 | 17163839-ThyroxiNe-Ma-1 |
| Negative | Sialyllactose | 17163839-ThyroxiNe-Ma-1 |
| Negative | HematoPorPhyriN IX | 21306108-AcetamiPrid-1 |
| Negative | Phorate | 21306108-AcetamiPrid-1 |
| Negative | Cellobiose | 21413891-BisPheNol A-1 |
| Negative | Sialyllactose | 21413891-BisPheNol A-1 |
| Negative | Cholic acid | 22261866-Pesticides-1 |
| Negative | Malachite greeN | 22261866-Pesticides-1 |
| Negative | Phorate | 22261866-Pesticides-1 |
| Negative | ThyroxiNe | 22261866-Pesticides-1 |
| Negative | AdeNosiNe triPhosPhate | 22261866-Pesticides-2 |
| Negative | ChitiN | 22261866-Pesticides-2 |
| Negative | OchratoxiN A | 22261866-Pesticides-2 |
| Negative | L-ArgiNiNe | 22261866-Pesticides-2 |
| Negative | N-AcetylNeuramiNic acid | 20000526-arseNic-1 |
| Negative | Sialyllactose | 20000526-arseNic-1 |
| Negative | AcetamiPrid | 20000526-arseNic-2 |
| Negative | ChitiN | 20000526-arseNic-2 |
| Negative | Cyclic adeNosiNe moNoPhosPhate | 20000526-arseNic-3 |
| Negative | Phorate | 20000526-arseNic-3 |
| Negative | ChitiN | 20000526-arseNic-4 |
| Negative | DoPamiNe | 20000526-arseNic-4 |
| Negative | Cellobiose | 20000526-arseNic-5 |
| Negative | L-ArgiNiNe | 20000526-arseNic-5 |
| Negative | AdeNiNe | 20000526-arseNic-6 |
| Negative | TyrosiNe | 20000526-arseNic-6 |
| Negative | FumoNisiN B1 | 20000526-arseNic-7 |
| Negative | IsoleuciNe | 20000526-arseNic-7 |
| Negative | AdeNiNe | 20000526-arseNic-8 |
| Negative | Glutamic acid | 20000526-arseNic-8 |
| Negative | TyrosiNe | 20000526-arseNic-Ma-1 |
| Negative | Glutamic acid | 20000526-arseNic-Ma-1 |
| Negative | BisPheNol A | 18187867-Glu-2 |
| Negative | N-GlycolylNeuramiNic acid | 18187867-Glu-2 |
| Negative | TryPtoPhaN | 23042406-Neu5Ac-1 |
| Negative | L-ArgiNiNe | 23042406-Neu5Ac-1 |
| Negative | HematoPorPhyriN IX | 23042406-Neu5Ac-1 |
| Negative | Omethoate | 23042406-Neu5Ac-1 |
| Negative | SulforhodamiNe B | 23042406-Neu5Ac-Ma-1 |
| Negative | ArseNate | 23042406-Neu5Ac-Ma-1 |
| Negative | Cholic acid | 23042406-Neu5Ac-2 |
| Negative | FumoNisiN B1 | 23042406-Neu5Ac-2 |
| Negative | Glutamic acid | 23042406-Neu5Ac-3 |
| Negative | ArseNate | 23042406-Neu5Ac-3 |
| Negative | TyrosiNe | 23042406-Neu5Ac-4 |
| Negative | Cyclic adeNosiNe moNoPhosPhate | 23042406-Neu5Ac-4 |
| Negative | HematoPorPhyriN IX | 23042406-Neu5Ac-5 |
| Negative | DoPamiNe | 23042406-Neu5Ac-5 |
| Negative | Malachite greeN | 23042406-Neu5Ac-6 |
| Negative | FADH | 23042406-Neu5Ac-6 |
| Negative | AdeNosiNe triPhosPhate | 23042406-Neu5Ac-7 |
| Negative | L-ArgiNiNe | 23042406-Neu5Ac-7 |
| Negative | FumoNisiN B1 | 10978501-cholic acid-1 |
| Negative | N-GlycolylNeuramiNic acid | 10978501-cholic acid-1 |
| Negative | DoPamiNe | 10978501-cholic acid-2 |
| Negative | Malachite greeN | 10978501-cholic acid-2 |
| Negative | IsoleuciNe | 10978501-cholic acid-3 |
| Negative | AcetamiPrid | 10978501-cholic acid-3 |
| Negative | L-ArgiNiNe | 10978501-cholic acid-4 |
| Negative | AdeNiNe | 10978501-cholic acid-4 |
| Negative | CodeiNe | 10978501-cholic acid-5 |
| Negative | AcetamiPrid | 10978501-cholic acid-5 |
| Negative | SulforhodamiNe B | 10978501-cholic acid-6 |
| Negative | AdeNosiNe triPhosPhate | 10978501-cholic acid-6 |
| Negative | TryPtoPhaN | 10978501-cholic acid-Ma-1 |
| Negative | OchratoxiN A | 10978501-cholic acid-Ma-1 |
