# Supplementary material for: Analysis and Identification of Aptamer-Compound Interactions with a Maximum Relevance Minimum Redundancy and Nearest Neighbor Algorithm
Source: Biomed Res Int. 2016 Feb 3;2016:8351204. doi: 10.1155/2016/8351204 (PMC4756144; doi:10.1155/2016/8351204)
Supplement: Supplementary file 1 — The Supplementary Material contains four files. In detail, the Supplementary Material I lists 159 positive interactions and 318 negative interactions; the Supplementary Material II lists MaxRel features list and mRMR features list; Supplementary Material III lists the SNs, SPs, ACCs and MCCs obtained by IFS and four basic prediction engines; Supplementary Material IV lists predicted results of all interactions obtained by the optimal prediction model. [file 8351204.f1.zip › Supp-II.docx]

**Supplemental Material II.** MaxRel features list and mRMR features list

(1) MaxRel features list

| **Rank** | **Feature name** |
| --- | --- |
| 1 | Tot dipole of the molecule |
| 2 | Tot point-charge comp. of the molecular dipole |
| 3 | DPSA-1 Difference in CPSAs (PPSA1-PNSA1) [Zefirov's PC] |
| 4 | HA dependent HDCA-2 [Zefirov's PC] |
| 5 | Max partial charge for a H atom [Zefirov's PC] |
| 6 | ESP-HA dependent HDSA-2 [Quantum-Chemical PC] |
| 7 | Number of double bonds |
| 8 | ESP-HA dependent HDCA-2 [Quantum-Chemical PC] |
| 9 | ESP-HACA-2 [Quantum-Chemical PC] |
| 10 | HA dependent HDSA-2 [Quantum-Chemical PC] |
| 11 | PNSA-3 Atomic charge weighted PNSA [Zefirov's PC] |
| 12 | Number of O atoms |
| 13 | Final heat of formation |
| 14 | ESP-Max net atomic charge for a H atom |
| 15 | HACA-2 [Zefirov's PC] |
| 16 | ESP-DPSA-1 Difference in CPSAs (PPSA1-PNSA1) [Quantum-Chemical PC] |
| 17 | HA dependent HDCA-2 [Quantum-Chemical PC] |
| 18 | HACA-1 [Zefirov's PC] |
| 19 | Average Structural Information content (order 1) |
| 20 | HOMO - LUMO energy gap |
| 21 | ESP-HA dependent HDSA-1 [Quantum-Chemical PC] |
| 22 | min(#HA_#HD) [Quantum-Chemical PC] |
| 23 | min(#HA_#HD) [Zefirov's PC] |
| 24 | ESP-count of H-acceptor sites [Quantum-Chemical PC] |
| 25 | count of H-acceptor sites [Zefirov's PC] |
| 26 | ESP-min(#HA_#HD) [Quantum-Chemical PC] |
| 27 | count of H-acceptor sites [Quantum-Chemical PC] |
| 28 | DPSA-1 Difference in CPSAs (PPSA1-PNSA1) [Quantum-Chemical PC] |
| 29 | HA dependent HDSA-1/TMSA [Zefirov's PC] |
| 30 | DPSA-3 Difference in CPSAs (PPSA3-PNSA3) [Zefirov's PC] |
| 31 | HA dependent HDCA-1 [Quantum-Chemical PC] |
| 32 | HA dependent HDCA-1 [Zefirov's PC] |
| 33 | ESP-HDSA H-donors surface area [Quantum-Chemical PC] |
| 34 | HASA-2 [Zefirov's PC] |
| 35 | HASA-2 [Quantum-Chemical PC] |
| 36 | ESP-HASA-2 [Quantum-Chemical PC] |
| 37 | (1/6)X GAMMA polarizability (DIP) |
| 38 | 1X GAMMA polarizability (DIP) |
| 39 | HA dependent HDSA-2/SQRT(TMSA) [Zefirov's PC] |
| 40 | RNCG Relative negative charge (QMNEG/QTMINUS) [Zefirov's PC] |
| 41 | HDSA H-donors surface area [Quantum-Chemical PC] |
| 42 | ESP-HA dependent HDCA-1 [Quantum-Chemical PC] |
| 43 | HASA-1 [Quantum-Chemical PC] |
| 44 | ESP-HASA-1 [Quantum-Chemical PC] |
| 45 | HASA-1 [Zefirov's PC] |
| 46 | Average Bonding Information content (order 1) |
| 47 | HA dependent HDSA-2 [Zefirov's PC] |
| 48 | HACA-2/SQRT(TMSA) [Quantum-Chemical PC] |
| 49 | Relative number of single bonds |
| 50 | Balaban index |
| 51 | ZX Shadow / ZX Rectangle |
| 52 | HDCA H-donors charged surface area [Quantum-Chemical PC] |
| 53 | ESP-HDCA H-donors charged surface area [Quantum-Chemical PC] |
| 54 | HASA-2/TMSA [Quantum-Chemical PC] |
| 55 | ESP-HASA-2/TMSA [Quantum-Chemical PC] |
| 56 | HASA-2/TMSA [Zefirov's PC] |
| 57 | ESP-HACA-2/SQRT(TMSA) [Quantum-Chemical PC] |
| 58 | ESP-HACA-1 [Quantum-Chemical PC] |
| 59 | HA dependent HDSA-1 [Zefirov's PC] |
| 60 | Number of benzene rings |
| 61 | HBSA H-bonding surface area [Quantum-Chemical PC] |
| 62 | Number of rings |
| 63 | RPCG Relative positive charge (QMPOS/QTPLUS) [Zefirov's PC] |
| 64 | PPSA-3 Atomic charge weighted PPSA [Zefirov's PC] |
| 65 | Max net atomic charge for a H atom |
| 66 | HA dependent HDSA-1 [Quantum-Chemical PC] |
| 67 | ESP-HBSA H-bonding surface area [Quantum-Chemical PC] |
| 68 | PNSA-1 Partial negative surface area [Quantum-Chemical PC] |
| 69 | Relative number of double bonds |
| 70 | Final heat of formation / # of atoms |
| 71 | FNSA-3 Fractional PNSA (PNSA-3/TMSA) [Zefirov's PC] |
| 72 | Topographic electronic index (all bonds) [Zefirov's PC] |
| 73 | ESP-PPSA-1 Partial positive surface area [Quantum-Chemical PC] |
| 74 | PPSA-1 Partial positive surface area [Quantum-Chemical PC] |
| 75 | RPCS Relative positive charged SA (SAMPOS*RPCG) [Zefirov's PC] |
| 76 | Molecular volume / XYZ Box |
| 77 | Number of C atoms |
| 78 | HACA-2 [Quantum-Chemical PC] |
| 79 | HACA-1 [Quantum-Chemical PC] |
| 80 | Min e-e repulsion for a H atom |
| 81 | Max partial charge (Qmax) |
| 82 | Polarity parameter (Qmax-Qmin) |
| 83 | Min e-n attraction for a H atom |
| 84 | FNSA-1 Fractional PNSA (PNSA-1/TMSA) [Zefirov's PC] |
| 85 | FPSA-1 Fractional PPSA (PPSA-1/TMSA) [Zefirov's PC] |
| 86 | Min valency of a H atom |
| 87 | Min net atomic charge for a H atom |
| 88 | ESP-PNSA-1 Partial negative surface area [Quantum-Chemical PC] |
| 89 | ESP-HACA-2/TMSA [Quantum-Chemical PC] |
| 90 | FPSA-2 Fractional PPSA (PPSA-2/TMSA) [Zefirov's PC] |
| 91 | HACA-2/TMSA [Quantum-Chemical PC] |
| 92 | Average Structural Information content (order 0) |
| 93 | Relative number of N atoms |
| 94 | ESP-HA dependent HDCA-2/SQRT(TMSA) [Quantum-Chemical PC] |
| 95 | Image of the Onsager-Kirkwood solvation energy |
| 96 | FNSA-2 Fractional PNSA (PNSA-2/TMSA) [Zefirov's PC] |
| 97 | HASA-2/SQRT(TMSA) [Zefirov's PC] |
| 98 | ESP-HASA-2/SQRT(TMSA) [Quantum-Chemical PC] |
| 99 | HASA-2/SQRT(TMSA) [Quantum-Chemical PC] |
| 100 | PPSA-1 Partial positive surface area [Zefirov's PC] |
| 101 | Relative number of benzene rings |
| 102 | Relative number of aromatic bonds |
| 103 | Average Information content (order 0) |
| 104 | Relative number of rings |
| 105 | Max SIGMA-PI bond order |
| 106 | ESP-HASA-1/TMSA [Quantum-Chemical PC] |
| 107 | HASA-1/TMSA [Zefirov's PC] |
| 108 | HASA-1/TMSA [Quantum-Chemical PC] |
| 109 | Number of N atoms |
| 110 | FHBCA Fractional HBSA (HBSA/TMSA) [Quantum-Chemical PC] |
| 111 | FHACA Fractional HACA (HACA/TMSA) [Quantum-Chemical PC] |
| 112 | YZ Shadow / YZ Rectangle |
| 113 | RNCS Relative negative charged SA (SAMNEG*RNCG) [Zefirov's PC] |
| 114 | HA dependent HDCA-2/SQRT(TMSA) [Quantum-Chemical PC] |
| 115 | 1X BETA polarizability (DIP) |
| 116 | (1/2)X BETA polarizability (DIP) |
| 117 | HASA H-acceptors surface area [Quantum-Chemical PC] |
| 118 | ESP-HASA H-acceptors surface area [Quantum-Chemical PC] |
| 119 | XY Shadow |
| 120 | PNSA-2 Total charge weighted PNSA [Zefirov's PC] |
| 121 | Average Complementary Information content (order 1) |
| 122 | Average Bonding Information content (order 0) |
| 123 | HA dependent HDSA-2/SQRT(TMSA) [Quantum-Chemical PC] |
| 124 | ESP-FNSA-1 Fractional PNSA (PNSA-1/TMSA) [Quantum-Chemical PC] |
| 125 | ESP-FPSA-1 Fractional PPSA (PPSA-1/TMSA) [Quantum-Chemical PC] |
| 126 | PNSA-1 Partial negative surface area [Zefirov's PC] |
| 127 | Min (>0.1) bond order of a H atom |
| 128 | FPSA-3 Fractional PPSA (PPSA-3/TMSA) [Zefirov's PC] |
| 129 | RNCS Relative negative charged SA (SAMNEG*RNCG) [Quantum-Chemical PC] |
| 130 | Relative number of O atoms |
| 131 | Min net atomic charge |
| 132 | WPSA-2 Weighted PPSA (PPSA2*TMSA/1000) [Quantum-Chemical PC] |
| 133 | ESP-HA dependent HDSA-2/SQRT(TMSA) [Quantum-Chemical PC] |
| 134 | Topographic electronic index (all pairs) [Zefirov's PC] |
| 135 | ZX Shadow |
| 136 | Kier flexibility index |
| 137 | PNSA-3 Atomic charge weighted PNSA [Quantum-Chemical PC] |
| 138 | DPSA-2 Difference in CPSAs (PPSA2-PNSA2) [Zefirov's PC] |
| 139 | WNSA-2 Weighted PNSA (PNSA2*TMSA/1000) [Zefirov's PC] |
| 140 | ESP-FHACA Fractional HACA (HACA/TMSA) [Quantum-Chemical PC] |
| 141 | ESP-HA dependent HDCA-2/TMSA [Quantum-Chemical PC] |
| 142 | Number of aromatic bonds |
| 143 | Min partial charge (Qmin) |
| 144 | Max atomic orbital electronic population |
| 145 | HACA-2/SQRT(TMSA) [Zefirov's PC] |
| 146 | ESP-HBCA H-bonding charged surface area [Quantum-Chemical PC] |
| 147 | Average Information content (order 1) |
| 148 | HOMO-1 energy |
| 149 | HA dependent HDSA-2/TMSA [Zefirov's PC] |
| 150 | HA dependent HDCA-2/SQRT(TMSA) [Zefirov's PC] |
| 151 | Max e-n attraction for a H atom |
| 152 | Max atomic state energy for a H atom |
| 153 | FPSA-2 Fractional PPSA (PPSA-2/TMSA) [Quantum-Chemical PC] |
| 154 | Min atomic orbital electronic population |
| 155 | FNSA-3 Fractional PNSA (PNSA-3/TMSA) [Quantum-Chemical PC] |
| 156 | ESP-HACA-1/TMSA [Quantum-Chemical PC] |
| 157 | XY Shadow / XY Rectangle |
| 158 | HA dependent HDSA-1/TMSA [Quantum-Chemical PC] |
| 159 | HACA H-acceptors charged surface area [Quantum-Chemical PC] |
| 160 | LUMO energy |
| 161 | Average Complementary Information content (order 0) |
| 162 | LUMO energy |
| 163 | RNCG Relative negative charge (QMNEG/QTMINUS) [Quantum-Chemical PC] |
| 164 | Randic index (order 3) |
| 165 | Number of atoms |
| 166 | Number of bonds |
| 167 | PPSA-3 Atomic charge weighted PPSA [Quantum-Chemical PC] |
| 168 | ESP-FHDCA Fractional HDCA (HDCA/TMSA) [Quantum-Chemical PC] |
| 169 | ESP-HA dependent HDCA-1/TMSA [Quantum-Chemical PC] |
| 170 | HA dependent HDCA-1/TMSA [Quantum-Chemical PC] |
| 171 | HOMO energy |
| 172 | Kier&Hall index (order 1) |
| 173 | ALFA polarizability (DIP) |
| 174 | ESP-RNCG Relative negative charge (QMNEG/QTMINUS) [Quantum-Chemical PC] |
| 175 | ESP-WNSA-2 Weighted PNSA (PNSA2*TMSA/1000) [Quantum-Chemical PC] |
| 176 | ESP-WPSA-2 Weighted PPSA (PPSA2*TMSA/1000) [Quantum-Chemical PC] |
| 177 | ESP-FHDSA Fractional HDSA (HDSA/TMSA) [Quantum-Chemical PC] |
| 178 | HA dependent HDSA-2/TMSA [Quantum-Chemical PC] |
| 179 | HACA-2/TMSA [Zefirov's PC] |
| 180 | ESP-FHBCA Fractional HBSA (HBSA/TMSA) [Quantum-Chemical PC] |
| 181 | HACA-1/TMSA [Quantum-Chemical PC] |
| 182 | Max PI-PI bond order |
| 183 | ESP-WNSA-1 Weighted PNSA (PNSA1*TMSA/1000) [Quantum-Chemical PC] |
| 184 | WPSA-1 Weighted PPSA (PPSA1*TMSA/1000) [Zefirov's PC] |
| 185 | ESP-WPSA-1 Weighted PPSA (PPSA1*TMSA/1000) [Quantum-Chemical PC] |
| 186 | WPSA-1 Weighted PPSA (PPSA1*TMSA/1000) [Quantum-Chemical PC] |
| 187 | Kier&Hall index (order 3) |
| 188 | HA dependent HDCA-2/TMSA [Zefirov's PC] |
| 189 | Relative number of H atoms |
| 190 | Average Information content (order 2) |
| 191 | DPSA-3 Difference in CPSAs (PPSA3-PNSA3) [Quantum-Chemical PC] |
| 192 | ESP-WNSA-3 Weighted PNSA (PNSA3*TMSA/1000) [Quantum-Chemical PC] |
| 193 | Information content (order 1) |
| 194 | Min atomic state energy for a H atom |
| 195 | YZ Shadow |
| 196 | Tot hybridization comp. of the molecular dipole |
| 197 | WNSA-3 Weighted PNSA (PNSA3*TMSA/1000) [Quantum-Chemical PC] |
| 198 | WPSA-3 Weighted PPSA (PPSA3*TMSA/1000) [Quantum-Chemical PC] |
| 199 | Complementary Information content (order 2) |
| 200 | ESP-TMSA Total molecular surface area [Quantum-Chemical PC] |
| 201 | TMSA Total molecular surface area [Zefirov's PC] |
| 202 | Molecular surface area |
| 203 | Randic index (order 0) |
| 204 | Tot molecular 2-center resonance energy |
| 205 | Molecular volume |
| 206 | Bonding Information content (order 1) |
| 207 | Structural Information content (order 1) |
| 208 | Randic index (order 1) |
| 209 | TMSA Total molecular surface area [Quantum-Chemical PC] |
| 210 | Average Structural Information content (order 2) |
| 211 | Polarity parameter / square distance |
| 212 | No. of occupied electronic levels |
| 213 | Information content (order 0) |
| 214 | Kier shape index (order 1) |
| 215 | Kier shape index (order 2) |
| 216 | Wiener index |
| 217 | ESP-HACA H-acceptors charged surface area [Quantum-Chemical PC] |
| 218 | FPSA-3 Fractional PPSA (PPSA-3/TMSA) [Quantum-Chemical PC] |
| 219 | HA dependent HDCA-2/TMSA [Quantum-Chemical PC] |
| 220 | ESP-HA dependent HDSA-2/TMSA [Quantum-Chemical PC] |
| 221 | Gravitation index (all bonds) |
| 222 | HA dependent HDCA-1/TMSA [Zefirov's PC] |
| 223 | FHDSA Fractional HDSA (HDSA/TMSA) [Quantum-Chemical PC] |
| 224 | ESP-FHBSA Fractional HBSA (HBSA/TMSA) [Quantum-Chemical PC] |
| 225 | WNSA-1 Weighted PNSA (PNSA1*TMSA/1000) [Zefirov's PC] |
| 226 | Average Complementary Information content (order 2) |
| 227 | Tot molecular 2-center resonance energy / # of atoms |
| 228 | Tot molecular 1-center E-N attraction |
| 229 | WPSA-3 Weighted PPSA (PPSA3*TMSA/1000) [Zefirov's PC] |
| 230 | Kier&Hall index (order 2) |
| 231 | Relative number of C atoms |
| 232 | Avg valency of a H atom |
| 233 | Moment of inertia A |
| 234 | Structural Information content (order 2) |
| 235 | Information content (order 2) |
| 236 | Bonding Information content (order 2) |
| 237 | ESP-FHASA Fractional HASA (HASA/TMSA) [Quantum-Chemical PC] |
| 238 | FHBSA Fractional HBSA (HBSA/TMSA) [Quantum-Chemical PC] |
| 239 | ESP-PPSA-3 Atomic charge weighted PPSA [Quantum-Chemical PC] |
| 240 | ESP-FPSA-2 Fractional PPSA (PPSA-2/TMSA) [Quantum-Chemical PC] |
| 241 | ESP-PNSA-3 Atomic charge weighted PNSA [Quantum-Chemical PC] |
| 242 | ESP-DPSA-3 Difference in CPSAs (PPSA3-PNSA3) [Quantum-Chemical PC] |
| 243 | ESP-WPSA-3 Weighted PPSA (PPSA3*TMSA/1000) [Quantum-Chemical PC] |
| 244 | ESP-FNSA-2 Fractional PNSA (PNSA-2/TMSA) [Quantum-Chemical PC] |
| 245 | ESP-FPSA-3 Fractional PPSA (PPSA-3/TMSA) [Quantum-Chemical PC] |
| 246 | ESP-DPSA-2 Difference in CPSAs (PPSA2-PNSA2) [Quantum-Chemical PC] |
| 247 | ESP-Min net atomic charge for a H atom |
| 248 | ESP-Min net atomic charge |
| 249 | ESP-Max net atomic charge |
| 250 | ESP-PNSA-2 Total charge weighted PNSA [Quantum-Chemical PC] |
| 251 | ESP-PPSA-2 Total charge weighted PPSA [Quantum-Chemical PC] |
| 252 | Avg bond order of a H atom |
| 253 | FNSA-1 Fractional PNSA (PNSA-1/TMSA) [Quantum-Chemical PC] |
| 254 | FPSA-1 Fractional PPSA (PPSA-1/TMSA) [Quantum-Chemical PC] |
| 255 | FNSA-2 Fractional PNSA (PNSA-2/TMSA) [Quantum-Chemical PC] |
| 256 | Bonding Information content (order 0) |
| 257 | Tot molecular 1-center E-E repulsion |
| 258 | Structural Information content (order 0) |
| 259 | ESP-HA dependent HDSA-1/TMSA [Quantum-Chemical PC] |
| 260 | PPSA-2 Total charge weighted PPSA [Quantum-Chemical PC] |
| 261 | HACA-1/TMSA [Zefirov's PC] |
| 262 | ESP-RPCG Relative positive charge (QMPOS/QTPLUS) [Quantum-Chemical PC] |
| 263 | ESP-RNCS Relative negative charged SA (SAMNEG*RNCG) [Quantum-Chemical PC] |
| 264 | Randic index (order 2) |
| 265 | Kier&Hall index (order 0) |
| 266 | Molecular weight |
| 267 | WPSA-2 Weighted PPSA (PPSA2*TMSA/1000) [Zefirov's PC] |
| 268 | WNSA-1 Weighted PNSA (PNSA1*TMSA/1000) [Quantum-Chemical PC] |
| 269 | PPSA-2 Total charge weighted PPSA [Zefirov's PC] |
| 270 | WNSA-3 Weighted PNSA (PNSA3*TMSA/1000) [Zefirov's PC] |
| 271 | RPCG Relative positive charge (QMPOS/QTPLUS) [Quantum-Chemical PC] |
| 272 | Gravitation index (all pairs) |
| 273 | Number of H atoms |
| 274 | Complementary Information content (order 0) |
| 275 | FHDCA Fractional HDCA (HDCA/TMSA) [Quantum-Chemical PC] |
| 276 | Tot molecular 1-center E-N attraction / # of atoms |
| 277 | Average Bonding Information content (order 2) |
| 278 | FHASA Fractional HASA (HASA/TMSA) [Quantum-Chemical PC] |
| 279 | Max net atomic charge |
| 280 | No. of occupied electronic levels / # of atoms |
| 281 | HBCA H-bonding charged surface area [Quantum-Chemical PC] |
| 282 | Max bond order of a H atom |
| 283 | DPSA-2 Difference in CPSAs (PPSA2-PNSA2) [Quantum-Chemical PC] |
| 284 | WNSA-2 Weighted PNSA (PNSA2*TMSA/1000) [Quantum-Chemical PC] |
| 285 | PNSA-2 Total charge weighted PNSA [Quantum-Chemical PC] |
| 286 | Moment of inertia C |
| 287 | ESP-RPCS Relative positive charged SA (SAMPOS*RPCG) [Quantum-Chemical PC] |
| 288 | Min partial charge for a H atom [Zefirov's PC] |
| 289 | Tot molecular 1-center E-E repulsion / # of atoms |
| 290 | Number of single bonds |
| 291 | count of H-donors sites [Quantum-Chemical PC] |
| 292 | Complementary Information content (order 1) |
| 293 | count of H-donors sites [Zefirov's PC] |
| 294 | ESP-count of H-donors sites [Quantum-Chemical PC] |
| 295 | Relative molecular weight |
| 296 | ESP-FNSA-3 Fractional PNSA (PNSA-3/TMSA) [Quantum-Chemical PC] |
| 297 | Max SIGMA-SIGMA bond order |
| 298 | Max e-e repulsion for a H atom |
| 299 | Max valency of a H atom |
| 300 | Moment of inertia B |
| 301 | RPCS Relative positive charged SA (SAMPOS*RPCG) [Quantum-Chemical PC] |
| 302 | t |
| 303 | a |
| 304 | tg |
| 305 | ct |
| 306 | gt |
| 307 | cc |
| 308 | aa |
| 309 | gc |
| 310 | cg |
| 311 | at |
| 312 | ca |
| 313 | g |
| 314 | tt |
| 315 | c |
| 316 | gg |
| 317 | ga |
| 318 | ta |
| 319 | ag |
| 320 | ac |

(2) mRMR features list

| **Rank** | **Feature name** |
| --- | --- |
| 1 | Tot dipole of the molecule |
| 2 | ESP-DPSA-1 Difference in CPSAs (PPSA1-PNSA1) [Quantum-Chemical PC] |
| 3 | PNSA-2 Total charge weighted PNSA [Zefirov's PC] |
| 4 | Min e-n attraction for a H atom |
| 5 | Relative number of N atoms |
| 6 | ESP-PNSA-3 Atomic charge weighted PNSA [Quantum-Chemical PC] |
| 7 | ga |
| 8 | gc |
| 9 | ZX Shadow / ZX Rectangle |
| 10 | HA dependent HDCA-2 [Zefirov's PC] |
| 11 | aa |
| 12 | (1/6)X GAMMA polarizability (DIP) |
| 13 | cc |
| 14 | Tot point-charge comp. of the molecular dipole |
| 15 | ESP-PPSA-3 Atomic charge weighted PPSA [Quantum-Chemical PC] |
| 16 | HA dependent HDSA-1/TMSA [Zefirov's PC] |
| 17 | ca |
| 18 | Min atomic orbital electronic population |
| 19 | ag |
| 20 | Min partial charge (Qmin) |
| 21 | WPSA-2 Weighted PPSA (PPSA2*TMSA/1000) [Quantum-Chemical PC] |
| 22 | Average Structural Information content (order 1) |
| 23 | Number of rings |
| 24 | ESP-FPSA-2 Fractional PPSA (PPSA-2/TMSA) [Quantum-Chemical PC] |
| 25 | tg |
| 26 | ESP-HA dependent HDSA-2 [Quantum-Chemical PC] |
| 27 | 1X GAMMA polarizability (DIP) |
| 28 | ESP-DPSA-3 Difference in CPSAs (PPSA3-PNSA3) [Quantum-Chemical PC] |
| 29 | ESP-Max net atomic charge for a H atom |
| 30 | ct |
| 31 | WNSA-2 Weighted PNSA (PNSA2*TMSA/1000) [Zefirov's PC] |
| 32 | cg |
| 33 | ESP-WPSA-3 Weighted PPSA (PPSA3*TMSA/1000) [Quantum-Chemical PC] |
| 34 | DPSA-1 Difference in CPSAs (PPSA1-PNSA1) [Zefirov's PC] |
| 35 | Number of double bonds |
| 36 | ESP-FNSA-2 Fractional PNSA (PNSA-2/TMSA) [Quantum-Chemical PC] |
| 37 | Max e-e repulsion for a H atom |
| 38 | ta |
| 39 | FNSA-2 Fractional PNSA (PNSA-2/TMSA) [Zefirov's PC] |
| 40 | gt |
| 41 | ESP-WNSA-2 Weighted PNSA (PNSA2*TMSA/1000) [Quantum-Chemical PC] |
| 42 | Relative number of benzene rings |
| 43 | Max partial charge for a H atom [Zefirov's PC] |
| 44 | ESP-FPSA-3 Fractional PPSA (PPSA-3/TMSA) [Quantum-Chemical PC] |
| 45 | c |
| 46 | DPSA-2 Difference in CPSAs (PPSA2-PNSA2) [Zefirov's PC] |
| 47 | Polarity parameter / square distance |
| 48 | Image of the Onsager-Kirkwood solvation energy |
| 49 | ESP-DPSA-2 Difference in CPSAs (PPSA2-PNSA2) [Quantum-Chemical PC] |
| 50 | Moment of inertia C |
| 51 | Average Information content (order 0) |
| 52 | tc |
| 53 | a |
| 54 | HOMO - LUMO energy gap |
| 55 | ESP-Min net atomic charge for a H atom |
| 56 | Min atomic state energy for a H atom |
| 57 | Number of O atoms |
| 58 | ESP-Min net atomic charge |
| 59 | Min net atomic charge for a H atom |
| 60 | ESP-WPSA-2 Weighted PPSA (PPSA2*TMSA/1000) [Quantum-Chemical PC] |
| 61 | 1X BETA polarizability (DIP) |
| 62 | ESP-HA dependent HDSA-1 [Quantum-Chemical PC] |
| 63 | ESP-Max net atomic charge |
| 64 | at |
| 65 | ESP-RPCS Relative positive charged SA (SAMPOS*RPCG) [Quantum-Chemical PC] |
| 66 | HACA-1 [Zefirov's PC] |
| 67 | ESP-PNSA-2 Total charge weighted PNSA [Quantum-Chemical PC] |
| 68 | Number of benzene rings |
| 69 | tt |
| 70 | ESP-PPSA-2 Total charge weighted PPSA [Quantum-Chemical PC] |
| 71 | Min partial charge for a H atom [Zefirov's PC] |
| 72 | min(#HA_#HD) [Quantum-Chemical PC] |
| 73 | Max partial charge (Qmax) |
| 74 | (1/2)X BETA polarizability (DIP) |
| 75 | WNSA-3 Weighted PNSA (PNSA3*TMSA/1000) [Quantum-Chemical PC] |
| 76 | WPSA-2 Weighted PPSA (PPSA2*TMSA/1000) [Zefirov's PC] |
| 77 | Max SIGMA-SIGMA bond order |
| 78 | Moment of inertia B |
| 79 | ESP-HACA-2 [Quantum-Chemical PC] |
| 80 | ac |
| 81 | WPSA-3 Weighted PPSA (PPSA3*TMSA/1000) [Quantum-Chemical PC] |
| 82 | Min (>0.1) bond order of a H atom |
| 83 | WNSA-1 Weighted PNSA (PNSA1*TMSA/1000) [Quantum-Chemical PC] |
| 84 | min(#HA_#HD) [Zefirov's PC] |
| 85 | RPCS Relative positive charged SA (SAMPOS*RPCG) [Quantum-Chemical PC] |
| 86 | Final heat of formation |
| 87 | t |
| 88 | Max bond order of a H atom |
| 89 | Average Bonding Information content (order 1) |
| 90 | Max PI-PI bond order |
| 91 | PPSA-2 Total charge weighted PPSA [Zefirov's PC] |
| 92 | ESP-WNSA-3 Weighted PNSA (PNSA3*TMSA/1000) [Quantum-Chemical PC] |
| 93 | ESP-HA dependent HDCA-2 [Quantum-Chemical PC] |
| 94 | Relative molecular weight |
| 95 | Max SIGMA-PI bond order |
| 96 | gg |
| 97 | Max valency of a H atom |
| 98 | Balaban index |
| 99 | PNSA-1 Partial negative surface area [Quantum-Chemical PC] |
| 100 | DPSA-1 Difference in CPSAs (PPSA1-PNSA1) [Quantum-Chemical PC] |
| 101 | Min valency of a H atom |
| 102 | ESP-FNSA-3 Fractional PNSA (PNSA-3/TMSA) [Quantum-Chemical PC] |
| 103 | Moment of inertia A |
| 104 | PNSA-3 Atomic charge weighted PNSA [Zefirov's PC] |
| 105 | Complementary Information content (order 2) |
| 106 | ESP-HDCA H-donors charged surface area [Quantum-Chemical PC] |
| 107 | HOMO-1 energy |
| 108 | Topographic electronic index (all bonds) [Zefirov's PC] |
| 109 | g |
| 110 | Polarity parameter (Qmax-Qmin) |
| 111 | WNSA-3 Weighted PNSA (PNSA3*TMSA/1000) [Zefirov's PC] |
| 112 | ESP-RPCG Relative positive charge (QMPOS/QTPLUS) [Quantum-Chemical PC] |
| 113 | ESP-count of H-acceptor sites [Quantum-Chemical PC] |
| 114 | XY Shadow / XY Rectangle |
| 115 | Avg valency of a H atom |
| 116 | FNSA-3 Fractional PNSA (PNSA-3/TMSA) [Zefirov's PC] |
| 117 | PPSA-2 Total charge weighted PPSA [Quantum-Chemical PC] |
| 118 | Max net atomic charge |
| 119 | Relative number of single bonds |
| 120 | LUMO energy |
| 121 | Final heat of formation / # of atoms |
| 122 | count of H-acceptor sites [Zefirov's PC] |
| 123 | ESP-RNCS Relative negative charged SA (SAMNEG*RNCG) [Quantum-Chemical PC] |
| 124 | FNSA-1 Fractional PNSA (PNSA-1/TMSA) [Zefirov's PC] |
| 125 | Max atomic orbital electronic population |
| 126 | WPSA-3 Weighted PPSA (PPSA3*TMSA/1000) [Zefirov's PC] |
| 127 | Relative number of rings |
| 128 | Molecular volume / XYZ Box |
| 129 | FPSA-3 Fractional PPSA (PPSA-3/TMSA) [Quantum-Chemical PC] |
| 130 | ESP-min(#HA_#HD) [Quantum-Chemical PC] |
| 131 | Average Complementary Information content (order 1) |
| 132 | RPCG Relative positive charge (QMPOS/QTPLUS) [Quantum-Chemical PC] |
| 133 | HOMO energy |
| 134 | Relative number of aromatic bonds |
| 135 | RNCS Relative negative charged SA (SAMNEG*RNCG) [Zefirov's PC] |
| 136 | FPSA-2 Fractional PPSA (PPSA-2/TMSA) [Quantum-Chemical PC] |
| 137 | count of H-acceptor sites [Quantum-Chemical PC] |
| 138 | HACA-1/TMSA [Quantum-Chemical PC] |
| 139 | Avg bond order of a H atom |
| 140 | Wiener index |
| 141 | HACA-2 [Zefirov's PC] |
| 142 | ESP-FHDSA Fractional HDSA (HDSA/TMSA) [Quantum-Chemical PC] |
| 143 | Number of N atoms |
| 144 | FPSA-1 Fractional PPSA (PPSA-1/TMSA) [Zefirov's PC] |
| 145 | Max net atomic charge for a H atom |
| 146 | Average Information content (order 2) |
| 147 | ESP-FHDCA Fractional HDCA (HDCA/TMSA) [Quantum-Chemical PC] |
| 148 | DPSA-3 Difference in CPSAs (PPSA3-PNSA3) [Zefirov's PC] |
| 149 | ESP-HBCA H-bonding charged surface area [Quantum-Chemical PC] |
| 150 | FNSA-2 Fractional PNSA (PNSA-2/TMSA) [Quantum-Chemical PC] |
| 151 | HA dependent HDSA-2/SQRT(TMSA) [Zefirov's PC] |
| 152 | FNSA-3 Fractional PNSA (PNSA-3/TMSA) [Quantum-Chemical PC] |
| 153 | ESP-PNSA-1 Partial negative surface area [Quantum-Chemical PC] |
| 154 | Number of C atoms |
| 155 | HA dependent HDCA-2 [Quantum-Chemical PC] |
| 156 | Average Bonding Information content (order 0) |
| 157 | DPSA-2 Difference in CPSAs (PPSA2-PNSA2) [Quantum-Chemical PC] |
| 158 | HACA-2/SQRT(TMSA) [Quantum-Chemical PC] |
| 159 | WNSA-1 Weighted PNSA (PNSA1*TMSA/1000) [Zefirov's PC] |
| 160 | HASA-2/TMSA [Quantum-Chemical PC] |
| 161 | Min e-e repulsion for a H atom |
| 162 | ESP-FNSA-1 Fractional PNSA (PNSA-1/TMSA) [Quantum-Chemical PC] |
| 163 | WNSA-2 Weighted PNSA (PNSA2*TMSA/1000) [Quantum-Chemical PC] |
| 164 | Relative number of H atoms |
| 165 | ESP-HDSA H-donors surface area [Quantum-Chemical PC] |
| 166 | YZ Shadow / YZ Rectangle |
| 167 | RPCS Relative positive charged SA (SAMPOS*RPCG) [Zefirov's PC] |
| 168 | RPCG Relative positive charge (QMPOS/QTPLUS) [Zefirov's PC] |
| 169 | PNSA-2 Total charge weighted PNSA [Quantum-Chemical PC] |
| 170 | No. of occupied electronic levels / # of atoms |
| 171 | Average Structural Information content (order 2) |
| 172 | LUMO energy |
| 173 | PPSA-3 Atomic charge weighted PPSA [Zefirov's PC] |
| 174 | Relative number of double bonds |
| 175 | ESP-FPSA-1 Fractional PPSA (PPSA-1/TMSA) [Quantum-Chemical PC] |
| 176 | PPSA-3 Atomic charge weighted PPSA [Quantum-Chemical PC] |
| 177 | ESP-HASA-2/TMSA [Quantum-Chemical PC] |
| 178 | HA dependent HDSA-2 [Quantum-Chemical PC] |
| 179 | Tot molecular 1-center E-N attraction / # of atoms |
| 180 | ESP-PPSA-1 Partial positive surface area [Quantum-Chemical PC] |
| 181 | HACA-2 [Quantum-Chemical PC] |
| 182 | Min net atomic charge |
| 183 | HA dependent HDSA-2 [Zefirov's PC] |
| 184 | ESP-HACA-1/TMSA [Quantum-Chemical PC] |
| 185 | Average Structural Information content (order 0) |
| 186 | PNSA-1 Partial negative surface area [Zefirov's PC] |
| 187 | Relative number of O atoms |
| 188 | PPSA-1 Partial positive surface area [Quantum-Chemical PC] |
| 189 | DPSA-3 Difference in CPSAs (PPSA3-PNSA3) [Quantum-Chemical PC] |
| 190 | HASA-2/TMSA [Zefirov's PC] |
| 191 | HA dependent HDSA-1 [Zefirov's PC] |
| 192 | ESP-FHBCA Fractional HBSA (HBSA/TMSA) [Quantum-Chemical PC] |
| 193 | RNCS Relative negative charged SA (SAMNEG*RNCG) [Quantum-Chemical PC] |
| 194 | RNCG Relative negative charge (QMNEG/QTMINUS) [Zefirov's PC] |
| 195 | Average Complementary Information content (order 0) |
| 196 | Average Complementary Information content (order 2) |
| 197 | HACA-1 [Quantum-Chemical PC] |
| 198 | Tot molecular 1-center E-E repulsion / # of atoms |
| 199 | HDSA H-donors surface area [Quantum-Chemical PC] |
| 200 | Relative number of C atoms |
| 201 | PNSA-3 Atomic charge weighted PNSA [Quantum-Chemical PC] |
| 202 | ZX Shadow |
| 203 | Tot molecular 2-center resonance energy / # of atoms |
| 204 | ESP-HASA-1/TMSA [Quantum-Chemical PC] |
| 205 | Topographic electronic index (all pairs) [Zefirov's PC] |
| 206 | Average Information content (order 1) |
| 207 | XY Shadow |
| 208 | ESP-HACA-2/SQRT(TMSA) [Quantum-Chemical PC] |
| 209 | ESP-HA dependent HDCA-2/TMSA [Quantum-Chemical PC] |
| 210 | Kier&Hall index (order 3) |
| 211 | Number of aromatic bonds |
| 212 | FHBCA Fractional HBSA (HBSA/TMSA) [Quantum-Chemical PC] |
| 213 | ESP-HA dependent HDCA-1 [Quantum-Chemical PC] |
| 214 | Average Bonding Information content (order 2) |
| 215 | PPSA-1 Partial positive surface area [Zefirov's PC] |
| 216 | FHACA Fractional HACA (HACA/TMSA) [Quantum-Chemical PC] |
| 217 | HA dependent HDCA-1 [Zefirov's PC] |
| 218 | Kier flexibility index |
| 219 | ESP-HACA-1 [Quantum-Chemical PC] |
| 220 | Tot hybridization comp. of the molecular dipole |
| 221 | HASA-1/TMSA [Zefirov's PC] |
| 222 | FPSA-2 Fractional PPSA (PPSA-2/TMSA) [Zefirov's PC] |
| 223 | ESP-HACA-2/TMSA [Quantum-Chemical PC] |
| 224 | FPSA-3 Fractional PPSA (PPSA-3/TMSA) [Zefirov's PC] |
| 225 | ALFA polarizability (DIP) |
| 226 | ESP-HBSA H-bonding surface area [Quantum-Chemical PC] |
| 227 | ESP-FHACA Fractional HACA (HACA/TMSA) [Quantum-Chemical PC] |
| 228 | RNCG Relative negative charge (QMNEG/QTMINUS) [Quantum-Chemical PC] |
| 229 | HA dependent HDSA-2/TMSA [Zefirov's PC] |
| 230 | HACA-2/TMSA [Quantum-Chemical PC] |
| 231 | HASA-2 [Zefirov's PC] |
| 232 | ESP-FHASA Fractional HASA (HASA/TMSA) [Quantum-Chemical PC] |
| 233 | HA dependent HDCA-1 [Quantum-Chemical PC] |
| 234 | Max e-n attraction for a H atom |
| 235 | HACA-2/TMSA [Zefirov's PC] |
| 236 | ESP-FHBSA Fractional HBSA (HBSA/TMSA) [Quantum-Chemical PC] |
| 237 | Max atomic state energy for a H atom |
| 238 | HA dependent HDSA-1 [Quantum-Chemical PC] |
| 239 | HA dependent HDCA-1/TMSA [Zefirov's PC] |
| 240 | HASA-2 [Quantum-Chemical PC] |
| 241 | ESP-HA dependent HDCA-2/SQRT(TMSA) [Quantum-Chemical PC] |
| 242 | Randic index (order 3) |
| 243 | HASA-1/TMSA [Quantum-Chemical PC] |
| 244 | HACA H-acceptors charged surface area [Quantum-Chemical PC] |
| 245 | HA dependent HDSA-1/TMSA [Quantum-Chemical PC] |
| 246 | ESP-HASA H-acceptors surface area [Quantum-Chemical PC] |
| 247 | Number of H atoms |
| 248 | ESP-HASA-2 [Quantum-Chemical PC] |
| 249 | Structural Information content (order 2) |
| 250 | Gravitation index (all bonds) |
| 251 | HA dependent HDSA-2/TMSA [Quantum-Chemical PC] |
| 252 | Complementary Information content (order 0) |
| 253 | FHBSA Fractional HBSA (HBSA/TMSA) [Quantum-Chemical PC] |
| 254 | ESP-RNCG Relative negative charge (QMNEG/QTMINUS) [Quantum-Chemical PC] |
| 255 | HASA-1 [Quantum-Chemical PC] |
| 256 | ESP-HA dependent HDCA-1/TMSA [Quantum-Chemical PC] |
| 257 | ESP-WNSA-1 Weighted PNSA (PNSA1*TMSA/1000) [Quantum-Chemical PC] |
| 258 | HASA H-acceptors surface area [Quantum-Chemical PC] |
| 259 | HA dependent HDCA-2/TMSA [Zefirov's PC] |
| 260 | HACA-2/SQRT(TMSA) [Zefirov's PC] |
| 261 | HDCA H-donors charged surface area [Quantum-Chemical PC] |
| 262 | Information content (order 2) |
| 263 | FNSA-1 Fractional PNSA (PNSA-1/TMSA) [Quantum-Chemical PC] |
| 264 | ESP-HA dependent HDSA-2/TMSA [Quantum-Chemical PC] |
| 265 | ESP-HACA H-acceptors charged surface area [Quantum-Chemical PC] |
| 266 | ESP-HASA-1 [Quantum-Chemical PC] |
| 267 | Number of single bonds |
| 268 | HA dependent HDSA-2/SQRT(TMSA) [Quantum-Chemical PC] |
| 269 | Bonding Information content (order 2) |
| 270 | HASA-1 [Zefirov's PC] |
| 271 | count of H-donors sites [Quantum-Chemical PC] |
| 272 | FPSA-1 Fractional PPSA (PPSA-1/TMSA) [Quantum-Chemical PC] |
| 273 | ESP-HA dependent HDSA-1/TMSA [Quantum-Chemical PC] |
| 274 | HBSA H-bonding surface area [Quantum-Chemical PC] |
| 275 | HACA-1/TMSA [Zefirov's PC] |
| 276 | Complementary Information content (order 1) |
| 277 | FHASA Fractional HASA (HASA/TMSA) [Quantum-Chemical PC] |
| 278 | Kier&Hall index (order 1) |
| 279 | ESP-HA dependent HDSA-2/SQRT(TMSA) [Quantum-Chemical PC] |
| 280 | count of H-donors sites [Zefirov's PC] |
| 281 | HA dependent HDCA-1/TMSA [Quantum-Chemical PC] |
| 282 | ESP-count of H-donors sites [Quantum-Chemical PC] |
| 283 | Tot molecular 1-center E-N attraction |
| 284 | WPSA-1 Weighted PPSA (PPSA1*TMSA/1000) [Zefirov's PC] |
| 285 | HASA-2/SQRT(TMSA) [Zefirov's PC] |
| 286 | ESP-WPSA-1 Weighted PPSA (PPSA1*TMSA/1000) [Quantum-Chemical PC] |
| 287 | ESP-HASA-2/SQRT(TMSA) [Quantum-Chemical PC] |
| 288 | WPSA-1 Weighted PPSA (PPSA1*TMSA/1000) [Quantum-Chemical PC] |
| 289 | FHDSA Fractional HDSA (HDSA/TMSA) [Quantum-Chemical PC] |
| 290 | Kier&Hall index (order 2) |
| 291 | HASA-2/SQRT(TMSA) [Quantum-Chemical PC] |
| 292 | HA dependent HDCA-2/TMSA [Quantum-Chemical PC] |
| 293 | Information content (order 0) |
| 294 | Number of atoms |
| 295 | HA dependent HDCA-2/SQRT(TMSA) [Zefirov's PC] |
| 296 | HBCA H-bonding charged surface area [Quantum-Chemical PC] |
| 297 | Number of bonds |
| 298 | HA dependent HDCA-2/SQRT(TMSA) [Quantum-Chemical PC] |
| 299 | Kier&Hall index (order 0) |
| 300 | YZ Shadow |
| 301 | FHDCA Fractional HDCA (HDCA/TMSA) [Quantum-Chemical PC] |
| 302 | Molecular weight |
| 303 | ESP-TMSA Total molecular surface area [Quantum-Chemical PC] |
| 304 | No. of occupied electronic levels |
| 305 | Kier shape index (order 1) |
| 306 | TMSA Total molecular surface area [Zefirov's PC] |
| 307 | Kier shape index (order 2) |
| 308 | Molecular surface area |
| 309 | Randic index (order 2) |
| 310 | Randic index (order 0) |
| 311 | Information content (order 1) |
| 312 | Bonding Information content (order 0) |
| 313 | Molecular volume |
| 314 | Tot molecular 1-center E-E repulsion |
| 315 | Bonding Information content (order 1) |
| 316 | Structural Information content (order 0) |
| 317 | Structural Information content (order 1) |
| 318 | Randic index (order 1) |
| 319 | TMSA Total molecular surface area [Quantum-Chemical PC] |
| 320 | Tot molecular 2-center resonance energy |
| 321 | Gravitation index (all pairs) |
