# Supplementary material for: Analysis and Identification of Aptamer-Compound Interactions with a Maximum Relevance Minimum Redundancy and Nearest Neighbor Algorithm
Source: Biomed Res Int. 2016 Feb 3;2016:8351204. doi: 10.1155/2016/8351204 (PMC4756144; doi:10.1155/2016/8351204)
Supplement: Supplementary file 1 — The Supplementary Material contains four files. In detail, the Supplementary Material I lists 159 positive interactions and 318 negative interactions; the Supplementary Material II lists MaxRel features list and mRMR features list; Supplementary Material III lists the SNs, SPs, ACCs and MCCs obtained by IFS and four basic prediction engines; Supplementary Material IV lists predicted results of all interactions obtained by the optimal prediction model. [file 8351204.f1.zip › Supp-III.docx]

**Supplemental Material III.** The SNs, SPs, ACCs and MCCs obtained by IFS and four basic prediction engines

1. Results for NNA

| **Number of features** | **SN** | **SP** | **ACC** | **MCC** |
| --- | --- | --- | --- | --- |
| 4 | 0.559748 | 0.764151 | 0.696017 | 0.321461 |
| 5 | 0.559748 | 0.764151 | 0.696017 | 0.321461 |
| 6 | 0.559748 | 0.764151 | 0.696017 | 0.321461 |
| 7 | 0.616352 | 0.798742 | 0.737945 | 0.413187 |
| 8 | 0.559748 | 0.811321 | 0.727463 | 0.37743 |
| 9 | 0.559748 | 0.814465 | 0.72956 | 0.381332 |
| 10 | 0.559748 | 0.811321 | 0.727463 | 0.37743 |
| 11 | 0.641509 | 0.811321 | 0.754717 | 0.450749 |
| 12 | 0.641509 | 0.811321 | 0.754717 | 0.450749 |
| 13 | 0.685535 | 0.820755 | 0.775681 | 0.501045 |
| 14 | 0.679245 | 0.820755 | 0.773585 | 0.495522 |
| 15 | 0.679245 | 0.820755 | 0.773585 | 0.495522 |
| 16 | 0.685535 | 0.820755 | 0.775681 | 0.501045 |
| 17 | 0.748428 | 0.823899 | 0.798742 | 0.559801 |
| 18 | 0.748428 | 0.827044 | 0.800839 | 0.56356 |
| 19 | 0.767296 | 0.830189 | 0.809224 | 0.583713 |
| 20 | 0.779874 | 0.836478 | 0.81761 | 0.602146 |
| 21 | 0.779874 | 0.836478 | 0.81761 | 0.602146 |
| 22 | 0.767296 | 0.836478 | 0.813417 | 0.591276 |
| 23 | 0.767296 | 0.842767 | 0.81761 | 0.598925 |
| 24 | 0.767296 | 0.842767 | 0.81761 | 0.598925 |
| 25 | 0.761006 | 0.842767 | 0.815514 | 0.593509 |
| 26 | 0.761006 | 0.839623 | 0.813417 | 0.589663 |
| 27 | 0.761006 | 0.839623 | 0.813417 | 0.589663 |
| 28 | 0.761006 | 0.839623 | 0.813417 | 0.589663 |
| 29 | 0.761006 | 0.833333 | 0.809224 | 0.582037 |
| 30 | 0.748428 | 0.842767 | 0.811321 | 0.582672 |
| 31 | 0.748428 | 0.842767 | 0.811321 | 0.582672 |
| 32 | 0.767296 | 0.830189 | 0.809224 | 0.583713 |
| 33 | 0.767296 | 0.830189 | 0.809224 | 0.583713 |
| 34 | 0.767296 | 0.830189 | 0.809224 | 0.583713 |
| 35 | 0.767296 | 0.830189 | 0.809224 | 0.583713 |
| 36 | 0.767296 | 0.830189 | 0.809224 | 0.583713 |
| 37 | 0.767296 | 0.830189 | 0.809224 | 0.583713 |
| 38 | 0.779874 | 0.852201 | 0.828092 | 0.62133 |
| 39 | 0.779874 | 0.852201 | 0.828092 | 0.62133 |
| 40 | 0.767296 | 0.858491 | 0.828092 | 0.618442 |
| 41 | 0.767296 | 0.858491 | 0.828092 | 0.618442 |
| 42 | 0.767296 | 0.858491 | 0.828092 | 0.618442 |
| 43 | 0.767296 | 0.855346 | 0.825996 | 0.614492 |
| 44 | 0.767296 | 0.855346 | 0.825996 | 0.614492 |
| 45 | 0.773585 | 0.86478 | 0.834382 | 0.631752 |
| 46 | 0.773585 | 0.86478 | 0.834382 | 0.631752 |
| 47 | 0.773585 | 0.86478 | 0.834382 | 0.631752 |
| 48 | 0.773585 | 0.86478 | 0.834382 | 0.631752 |
| 49 | 0.773585 | 0.86478 | 0.834382 | 0.631752 |
| 50 | 0.773585 | 0.86478 | 0.834382 | 0.631752 |
| 51 | 0.773585 | 0.86478 | 0.834382 | 0.631752 |
| 52 | 0.767296 | 0.877358 | 0.840671 | 0.642662 |
| 53 | 0.761006 | 0.871069 | 0.834382 | 0.62917 |
| 54 | 0.761006 | 0.871069 | 0.834382 | 0.62917 |
| 55 | 0.761006 | 0.871069 | 0.834382 | 0.62917 |
| 56 | 0.761006 | 0.871069 | 0.834382 | 0.62917 |
| 57 | 0.761006 | 0.871069 | 0.834382 | 0.62917 |
| 58 | 0.761006 | 0.871069 | 0.834382 | 0.62917 |
| 59 | 0.761006 | 0.871069 | 0.834382 | 0.62917 |
| 60 | 0.761006 | 0.871069 | 0.834382 | 0.62917 |
| 61 | 0.761006 | 0.871069 | 0.834382 | 0.62917 |
| 62 | 0.761006 | 0.871069 | 0.834382 | 0.62917 |
| 63 | 0.761006 | 0.871069 | 0.834382 | 0.62917 |
| 64 | 0.761006 | 0.877358 | 0.838574 | 0.63737 |
| 65 | 0.761006 | 0.877358 | 0.838574 | 0.63737 |
| 66 | 0.761006 | 0.877358 | 0.838574 | 0.63737 |
| 67 | 0.761006 | 0.877358 | 0.838574 | 0.63737 |
| 68 | 0.761006 | 0.877358 | 0.838574 | 0.63737 |
| 69 | 0.786164 | 0.874214 | 0.844864 | 0.654463 |
| 70 | 0.786164 | 0.874214 | 0.844864 | 0.654463 |
| 71 | 0.786164 | 0.877358 | 0.84696 | 0.658527 |
| 72 | 0.786164 | 0.874214 | 0.844864 | 0.654463 |
| 73 | 0.786164 | 0.874214 | 0.844864 | 0.654463 |
| 74 | 0.786164 | 0.874214 | 0.844864 | 0.654463 |
| 75 | 0.786164 | 0.874214 | 0.844864 | 0.654463 |
| 76 | 0.786164 | 0.874214 | 0.844864 | 0.654463 |
| 77 | 0.786164 | 0.874214 | 0.844864 | 0.654463 |
| 78 | 0.786164 | 0.874214 | 0.844864 | 0.654463 |
| 79 | 0.786164 | 0.874214 | 0.844864 | 0.654463 |
| 80 | 0.779874 | 0.889937 | 0.853249 | 0.669811 |
| 81 | 0.779874 | 0.889937 | 0.853249 | 0.669811 |
| 82 | 0.779874 | 0.889937 | 0.853249 | 0.669811 |
| 83 | 0.779874 | 0.889937 | 0.853249 | 0.669811 |
| 84 | 0.779874 | 0.889937 | 0.853249 | 0.669811 |
| 85 | 0.779874 | 0.889937 | 0.853249 | 0.669811 |
| 86 | 0.779874 | 0.889937 | 0.853249 | 0.669811 |
| 87 | 0.773585 | 0.889937 | 0.851153 | 0.664574 |
| 88 | 0.773585 | 0.889937 | 0.851153 | 0.664574 |
| 89 | 0.773585 | 0.889937 | 0.851153 | 0.664574 |
| 90 | 0.773585 | 0.889937 | 0.851153 | 0.664574 |
| 91 | 0.773585 | 0.889937 | 0.851153 | 0.664574 |
| 92 | 0.773585 | 0.889937 | 0.851153 | 0.664574 |
| 93 | 0.773585 | 0.889937 | 0.851153 | 0.664574 |
| 94 | 0.773585 | 0.889937 | 0.851153 | 0.664574 |
| 95 | 0.773585 | 0.889937 | 0.851153 | 0.664574 |
| 96 | 0.773585 | 0.889937 | 0.851153 | 0.664574 |
| 97 | 0.773585 | 0.886792 | 0.849057 | 0.660377 |
| 98 | 0.773585 | 0.886792 | 0.849057 | 0.660377 |
| 99 | 0.773585 | 0.886792 | 0.849057 | 0.660377 |
| 100 | 0.773585 | 0.886792 | 0.849057 | 0.660377 |
| 101 | 0.773585 | 0.886792 | 0.849057 | 0.660377 |
| 102 | 0.773585 | 0.886792 | 0.849057 | 0.660377 |
| 103 | 0.773585 | 0.886792 | 0.849057 | 0.660377 |
| 104 | 0.773585 | 0.886792 | 0.849057 | 0.660377 |
| 105 | 0.773585 | 0.886792 | 0.849057 | 0.660377 |
| 106 | 0.773585 | 0.886792 | 0.849057 | 0.660377 |
| 107 | 0.773585 | 0.886792 | 0.849057 | 0.660377 |
| 108 | 0.773585 | 0.886792 | 0.849057 | 0.660377 |
| 109 | 0.773585 | 0.883648 | 0.84696 | 0.656208 |
| 110 | 0.773585 | 0.883648 | 0.84696 | 0.656208 |
| 111 | 0.773585 | 0.883648 | 0.84696 | 0.656208 |
| 112 | 0.773585 | 0.883648 | 0.84696 | 0.656208 |
| 113 | 0.773585 | 0.883648 | 0.84696 | 0.656208 |
| 114 | 0.773585 | 0.883648 | 0.84696 | 0.656208 |
| 115 | 0.773585 | 0.883648 | 0.84696 | 0.656208 |
| 116 | 0.773585 | 0.883648 | 0.84696 | 0.656208 |
| 117 | 0.773585 | 0.883648 | 0.84696 | 0.656208 |
| 118 | 0.773585 | 0.883648 | 0.84696 | 0.656208 |
| 119 | 0.773585 | 0.880503 | 0.844864 | 0.652066 |
| 120 | 0.773585 | 0.880503 | 0.844864 | 0.652066 |
| 121 | 0.773585 | 0.880503 | 0.844864 | 0.652066 |
| 122 | 0.773585 | 0.880503 | 0.844864 | 0.652066 |
| 123 | 0.773585 | 0.880503 | 0.844864 | 0.652066 |
| 124 | 0.773585 | 0.883648 | 0.84696 | 0.656208 |
| 125 | 0.773585 | 0.883648 | 0.84696 | 0.656208 |
| 126 | 0.773585 | 0.883648 | 0.84696 | 0.656208 |
| 127 | 0.773585 | 0.883648 | 0.84696 | 0.656208 |
| 128 | 0.773585 | 0.883648 | 0.84696 | 0.656208 |
| 129 | 0.773585 | 0.883648 | 0.84696 | 0.656208 |
| 130 | 0.773585 | 0.883648 | 0.84696 | 0.656208 |
| 131 | 0.773585 | 0.883648 | 0.84696 | 0.656208 |
| 132 | 0.773585 | 0.883648 | 0.84696 | 0.656208 |
| 133 | 0.773585 | 0.883648 | 0.84696 | 0.656208 |
| 134 | 0.773585 | 0.883648 | 0.84696 | 0.656208 |
| 135 | 0.773585 | 0.883648 | 0.84696 | 0.656208 |
| 136 | 0.773585 | 0.883648 | 0.84696 | 0.656208 |
| 137 | 0.773585 | 0.883648 | 0.84696 | 0.656208 |
| 138 | 0.773585 | 0.883648 | 0.84696 | 0.656208 |
| 139 | 0.773585 | 0.883648 | 0.84696 | 0.656208 |
| 140 | 0.773585 | 0.883648 | 0.84696 | 0.656208 |
| 141 | 0.773585 | 0.883648 | 0.84696 | 0.656208 |
| 142 | 0.773585 | 0.883648 | 0.84696 | 0.656208 |
| 143 | 0.773585 | 0.883648 | 0.84696 | 0.656208 |
| 144 | 0.773585 | 0.883648 | 0.84696 | 0.656208 |
| 145 | 0.773585 | 0.883648 | 0.84696 | 0.656208 |
| 146 | 0.773585 | 0.883648 | 0.84696 | 0.656208 |
| 147 | 0.773585 | 0.883648 | 0.84696 | 0.656208 |
| 148 | 0.773585 | 0.883648 | 0.84696 | 0.656208 |
| 149 | 0.773585 | 0.883648 | 0.84696 | 0.656208 |
| 150 | 0.773585 | 0.883648 | 0.84696 | 0.656208 |
| 151 | 0.773585 | 0.883648 | 0.84696 | 0.656208 |
| 152 | 0.773585 | 0.883648 | 0.84696 | 0.656208 |
| 153 | 0.773585 | 0.883648 | 0.84696 | 0.656208 |
| 154 | 0.773585 | 0.883648 | 0.84696 | 0.656208 |
| 155 | 0.773585 | 0.883648 | 0.84696 | 0.656208 |
| 156 | 0.773585 | 0.883648 | 0.84696 | 0.656208 |
| 157 | 0.773585 | 0.883648 | 0.84696 | 0.656208 |
| 158 | 0.773585 | 0.883648 | 0.84696 | 0.656208 |
| 159 | 0.773585 | 0.883648 | 0.84696 | 0.656208 |
| 160 | 0.773585 | 0.883648 | 0.84696 | 0.656208 |
| 161 | 0.773585 | 0.883648 | 0.84696 | 0.656208 |
| 162 | 0.773585 | 0.883648 | 0.84696 | 0.656208 |
| 163 | 0.773585 | 0.883648 | 0.84696 | 0.656208 |
| 164 | 0.773585 | 0.883648 | 0.84696 | 0.656208 |
| 165 | 0.773585 | 0.883648 | 0.84696 | 0.656208 |
| 166 | 0.773585 | 0.883648 | 0.84696 | 0.656208 |
| 167 | 0.773585 | 0.883648 | 0.84696 | 0.656208 |
| 168 | 0.773585 | 0.883648 | 0.84696 | 0.656208 |
| 169 | 0.773585 | 0.883648 | 0.84696 | 0.656208 |
| 170 | 0.773585 | 0.883648 | 0.84696 | 0.656208 |
| 171 | 0.773585 | 0.883648 | 0.84696 | 0.656208 |
| 172 | 0.773585 | 0.883648 | 0.84696 | 0.656208 |
| 173 | 0.773585 | 0.883648 | 0.84696 | 0.656208 |
| 174 | 0.773585 | 0.883648 | 0.84696 | 0.656208 |
| 175 | 0.773585 | 0.883648 | 0.84696 | 0.656208 |
| 176 | 0.773585 | 0.883648 | 0.84696 | 0.656208 |
| 177 | 0.773585 | 0.883648 | 0.84696 | 0.656208 |
| 178 | 0.773585 | 0.883648 | 0.84696 | 0.656208 |
| 179 | 0.773585 | 0.883648 | 0.84696 | 0.656208 |
| 180 | 0.773585 | 0.883648 | 0.84696 | 0.656208 |
| 181 | 0.773585 | 0.883648 | 0.84696 | 0.656208 |
| 182 | 0.773585 | 0.883648 | 0.84696 | 0.656208 |
| 183 | 0.773585 | 0.883648 | 0.84696 | 0.656208 |
| 184 | 0.773585 | 0.883648 | 0.84696 | 0.656208 |
| 185 | 0.773585 | 0.883648 | 0.84696 | 0.656208 |
| 186 | 0.773585 | 0.883648 | 0.84696 | 0.656208 |
| 187 | 0.773585 | 0.883648 | 0.84696 | 0.656208 |
| 188 | 0.773585 | 0.883648 | 0.84696 | 0.656208 |
| 189 | 0.773585 | 0.883648 | 0.84696 | 0.656208 |
| 190 | 0.773585 | 0.883648 | 0.84696 | 0.656208 |
| 191 | 0.773585 | 0.883648 | 0.84696 | 0.656208 |
| 192 | 0.773585 | 0.883648 | 0.84696 | 0.656208 |
| 193 | 0.773585 | 0.883648 | 0.84696 | 0.656208 |
| 194 | 0.773585 | 0.883648 | 0.84696 | 0.656208 |
| 195 | 0.773585 | 0.883648 | 0.84696 | 0.656208 |
| 196 | 0.773585 | 0.883648 | 0.84696 | 0.656208 |
| 197 | 0.773585 | 0.883648 | 0.84696 | 0.656208 |
| 198 | 0.773585 | 0.883648 | 0.84696 | 0.656208 |
| 199 | 0.773585 | 0.883648 | 0.84696 | 0.656208 |
| 200 | 0.773585 | 0.883648 | 0.84696 | 0.656208 |
| 201 | 0.773585 | 0.883648 | 0.84696 | 0.656208 |
| 202 | 0.773585 | 0.883648 | 0.84696 | 0.656208 |
| 203 | 0.773585 | 0.883648 | 0.84696 | 0.656208 |
| 204 | 0.773585 | 0.883648 | 0.84696 | 0.656208 |
| 205 | 0.773585 | 0.883648 | 0.84696 | 0.656208 |
| 206 | 0.773585 | 0.883648 | 0.84696 | 0.656208 |
| 207 | 0.773585 | 0.883648 | 0.84696 | 0.656208 |
| 208 | 0.773585 | 0.883648 | 0.84696 | 0.656208 |
| 209 | 0.773585 | 0.883648 | 0.84696 | 0.656208 |
| 210 | 0.773585 | 0.883648 | 0.84696 | 0.656208 |
| 211 | 0.773585 | 0.883648 | 0.84696 | 0.656208 |
| 212 | 0.773585 | 0.883648 | 0.84696 | 0.656208 |
| 213 | 0.773585 | 0.883648 | 0.84696 | 0.656208 |
| 214 | 0.773585 | 0.883648 | 0.84696 | 0.656208 |
| 215 | 0.773585 | 0.883648 | 0.84696 | 0.656208 |
| 216 | 0.773585 | 0.883648 | 0.84696 | 0.656208 |
| 217 | 0.773585 | 0.883648 | 0.84696 | 0.656208 |
| 218 | 0.773585 | 0.883648 | 0.84696 | 0.656208 |
| 219 | 0.773585 | 0.883648 | 0.84696 | 0.656208 |
| 220 | 0.773585 | 0.883648 | 0.84696 | 0.656208 |
| 221 | 0.773585 | 0.883648 | 0.84696 | 0.656208 |
| 222 | 0.773585 | 0.883648 | 0.84696 | 0.656208 |
| 223 | 0.773585 | 0.883648 | 0.84696 | 0.656208 |
| 224 | 0.773585 | 0.883648 | 0.84696 | 0.656208 |
| 225 | 0.773585 | 0.883648 | 0.84696 | 0.656208 |
| 226 | 0.773585 | 0.883648 | 0.84696 | 0.656208 |
| 227 | 0.773585 | 0.883648 | 0.84696 | 0.656208 |
| 228 | 0.773585 | 0.883648 | 0.84696 | 0.656208 |
| 229 | 0.773585 | 0.883648 | 0.84696 | 0.656208 |
| 230 | 0.773585 | 0.883648 | 0.84696 | 0.656208 |
| 231 | 0.773585 | 0.883648 | 0.84696 | 0.656208 |
| 232 | 0.767296 | 0.883648 | 0.844864 | 0.650943 |
| 233 | 0.767296 | 0.883648 | 0.844864 | 0.650943 |
| 234 | 0.767296 | 0.883648 | 0.844864 | 0.650943 |
| 235 | 0.767296 | 0.883648 | 0.844864 | 0.650943 |
| 236 | 0.767296 | 0.883648 | 0.844864 | 0.650943 |
| 237 | 0.767296 | 0.883648 | 0.844864 | 0.650943 |
| 238 | 0.767296 | 0.883648 | 0.844864 | 0.650943 |
| 239 | 0.767296 | 0.883648 | 0.844864 | 0.650943 |
| 240 | 0.767296 | 0.883648 | 0.844864 | 0.650943 |
| 241 | 0.767296 | 0.883648 | 0.844864 | 0.650943 |
| 242 | 0.767296 | 0.883648 | 0.844864 | 0.650943 |
| 243 | 0.767296 | 0.883648 | 0.844864 | 0.650943 |
| 244 | 0.767296 | 0.883648 | 0.844864 | 0.650943 |
| 245 | 0.767296 | 0.883648 | 0.844864 | 0.650943 |
| 246 | 0.767296 | 0.883648 | 0.844864 | 0.650943 |
| 247 | 0.767296 | 0.883648 | 0.844864 | 0.650943 |
| 248 | 0.767296 | 0.883648 | 0.844864 | 0.650943 |
| 249 | 0.767296 | 0.883648 | 0.844864 | 0.650943 |
| 250 | 0.767296 | 0.883648 | 0.844864 | 0.650943 |
| 251 | 0.767296 | 0.883648 | 0.844864 | 0.650943 |
| 252 | 0.767296 | 0.883648 | 0.844864 | 0.650943 |
| 253 | 0.767296 | 0.883648 | 0.844864 | 0.650943 |
| 254 | 0.767296 | 0.883648 | 0.844864 | 0.650943 |
| 255 | 0.767296 | 0.883648 | 0.844864 | 0.650943 |
| 256 | 0.767296 | 0.883648 | 0.844864 | 0.650943 |
| 257 | 0.767296 | 0.883648 | 0.844864 | 0.650943 |
| 258 | 0.767296 | 0.883648 | 0.844864 | 0.650943 |
| 259 | 0.767296 | 0.883648 | 0.844864 | 0.650943 |
| 260 | 0.767296 | 0.883648 | 0.844864 | 0.650943 |
| 261 | 0.767296 | 0.883648 | 0.844864 | 0.650943 |
| 262 | 0.767296 | 0.883648 | 0.844864 | 0.650943 |
| 263 | 0.767296 | 0.883648 | 0.844864 | 0.650943 |
| 264 | 0.767296 | 0.883648 | 0.844864 | 0.650943 |
| 265 | 0.767296 | 0.883648 | 0.844864 | 0.650943 |
| 266 | 0.767296 | 0.883648 | 0.844864 | 0.650943 |
| 267 | 0.767296 | 0.883648 | 0.844864 | 0.650943 |
| 268 | 0.767296 | 0.883648 | 0.844864 | 0.650943 |
| 269 | 0.767296 | 0.883648 | 0.844864 | 0.650943 |
| 270 | 0.767296 | 0.883648 | 0.844864 | 0.650943 |
| 271 | 0.767296 | 0.883648 | 0.844864 | 0.650943 |
| 272 | 0.767296 | 0.883648 | 0.844864 | 0.650943 |
| 273 | 0.767296 | 0.883648 | 0.844864 | 0.650943 |
| 274 | 0.767296 | 0.883648 | 0.844864 | 0.650943 |
| 275 | 0.767296 | 0.883648 | 0.844864 | 0.650943 |
| 276 | 0.767296 | 0.883648 | 0.844864 | 0.650943 |
| 277 | 0.767296 | 0.883648 | 0.844864 | 0.650943 |
| 278 | 0.767296 | 0.883648 | 0.844864 | 0.650943 |
| 279 | 0.767296 | 0.883648 | 0.844864 | 0.650943 |
| 280 | 0.767296 | 0.883648 | 0.844864 | 0.650943 |
| 281 | 0.767296 | 0.883648 | 0.844864 | 0.650943 |
| 282 | 0.767296 | 0.883648 | 0.844864 | 0.650943 |
| 283 | 0.767296 | 0.883648 | 0.844864 | 0.650943 |
| 284 | 0.767296 | 0.883648 | 0.844864 | 0.650943 |
| 285 | 0.767296 | 0.883648 | 0.844864 | 0.650943 |
| 286 | 0.767296 | 0.883648 | 0.844864 | 0.650943 |
| 287 | 0.767296 | 0.883648 | 0.844864 | 0.650943 |
| 288 | 0.767296 | 0.883648 | 0.844864 | 0.650943 |
| 289 | 0.767296 | 0.883648 | 0.844864 | 0.650943 |
| 290 | 0.767296 | 0.883648 | 0.844864 | 0.650943 |
| 291 | 0.767296 | 0.883648 | 0.844864 | 0.650943 |
| 292 | 0.767296 | 0.883648 | 0.844864 | 0.650943 |
| 293 | 0.767296 | 0.883648 | 0.844864 | 0.650943 |
| 294 | 0.767296 | 0.883648 | 0.844864 | 0.650943 |
| 295 | 0.767296 | 0.883648 | 0.844864 | 0.650943 |
| 296 | 0.767296 | 0.883648 | 0.844864 | 0.650943 |
| 297 | 0.767296 | 0.883648 | 0.844864 | 0.650943 |
| 298 | 0.767296 | 0.883648 | 0.844864 | 0.650943 |
| 299 | 0.767296 | 0.883648 | 0.844864 | 0.650943 |
| 300 | 0.767296 | 0.883648 | 0.844864 | 0.650943 |
| 301 | 0.767296 | 0.883648 | 0.844864 | 0.650943 |
| 302 | 0.767296 | 0.883648 | 0.844864 | 0.650943 |
| 303 | 0.767296 | 0.883648 | 0.844864 | 0.650943 |
| 304 | 0.767296 | 0.883648 | 0.844864 | 0.650943 |
| 305 | 0.767296 | 0.883648 | 0.844864 | 0.650943 |
| 306 | 0.767296 | 0.883648 | 0.844864 | 0.650943 |
| 307 | 0.767296 | 0.883648 | 0.844864 | 0.650943 |
| 308 | 0.767296 | 0.883648 | 0.844864 | 0.650943 |
| 309 | 0.767296 | 0.883648 | 0.844864 | 0.650943 |
| 310 | 0.767296 | 0.883648 | 0.844864 | 0.650943 |
| 311 | 0.767296 | 0.883648 | 0.844864 | 0.650943 |
| 312 | 0.767296 | 0.883648 | 0.844864 | 0.650943 |
| 313 | 0.767296 | 0.883648 | 0.844864 | 0.650943 |
| 314 | 0.767296 | 0.883648 | 0.844864 | 0.650943 |
| 315 | 0.767296 | 0.883648 | 0.844864 | 0.650943 |
| 316 | 0.767296 | 0.883648 | 0.844864 | 0.650943 |
| 317 | 0.767296 | 0.883648 | 0.844864 | 0.650943 |
| 318 | 0.767296 | 0.883648 | 0.844864 | 0.650943 |
| 319 | 0.767296 | 0.883648 | 0.844864 | 0.650943 |
| 320 | 0.767296 | 0.883648 | 0.844864 | 0.650943 |
| 321 | 0.767296 | 0.883648 | 0.844864 | 0.650943 |

1. Results for RF

| **Number of features** | **SN** | **SP** | **ACC** | **MCC** |
| --- | --- | --- | --- | --- |
| 4 | 0.540881 | 0.858491 | 0.752621 | 0.421808 |
| 5 | 0.540881 | 0.858491 | 0.752621 | 0.421808 |
| 6 | 0.540881 | 0.858491 | 0.752621 | 0.421808 |
| 7 | 0.63522 | 0.795597 | 0.742138 | 0.426355 |
| 8 | 0.622642 | 0.839623 | 0.767296 | 0.469335 |
| 9 | 0.616352 | 0.808176 | 0.744235 | 0.424528 |
| 10 | 0.63522 | 0.836478 | 0.769392 | 0.476385 |
| 11 | 0.691824 | 0.833333 | 0.786164 | 0.521967 |
| 12 | 0.654088 | 0.808176 | 0.756813 | 0.458124 |
| 13 | 0.698113 | 0.836478 | 0.790356 | 0.531343 |
| 14 | 0.647799 | 0.830189 | 0.769392 | 0.479517 |
| 15 | 0.685535 | 0.852201 | 0.796646 | 0.540339 |
| 16 | 0.679245 | 0.827044 | 0.777778 | 0.503213 |
| 17 | 0.691824 | 0.855346 | 0.800839 | 0.549819 |
| 18 | 0.716981 | 0.849057 | 0.805031 | 0.563436 |
| 19 | 0.685535 | 0.836478 | 0.786164 | 0.520399 |
| 20 | 0.679245 | 0.86478 | 0.802935 | 0.551358 |
| 21 | 0.704403 | 0.871069 | 0.815514 | 0.58119 |
| 22 | 0.672956 | 0.861635 | 0.798742 | 0.541797 |
| 23 | 0.691824 | 0.858491 | 0.802935 | 0.553897 |
| 24 | 0.716981 | 0.845912 | 0.802935 | 0.559473 |
| 25 | 0.704403 | 0.858491 | 0.807128 | 0.564694 |
| 26 | 0.685535 | 0.849057 | 0.794549 | 0.536302 |
| 27 | 0.698113 | 0.867925 | 0.811321 | 0.571662 |
| 28 | 0.716981 | 0.852201 | 0.807128 | 0.567423 |
| 29 | 0.698113 | 0.839623 | 0.792453 | 0.535264 |
| 30 | 0.660377 | 0.858491 | 0.792453 | 0.526804 |
| 31 | 0.698113 | 0.861635 | 0.807128 | 0.563393 |
| 32 | 0.660377 | 0.858491 | 0.792453 | 0.526804 |
| 33 | 0.660377 | 0.842767 | 0.781971 | 0.50642 |
| 34 | 0.704403 | 0.842767 | 0.796646 | 0.544655 |
| 35 | 0.685535 | 0.833333 | 0.784067 | 0.516483 |
| 36 | 0.691824 | 0.836478 | 0.78826 | 0.525874 |
| 37 | 0.641509 | 0.86478 | 0.790356 | 0.518869 |
| 38 | 0.716981 | 0.871069 | 0.819706 | 0.591879 |
| 39 | 0.691824 | 0.877358 | 0.815514 | 0.57894 |
| 40 | 0.679245 | 0.849057 | 0.792453 | 0.530859 |
| 41 | 0.660377 | 0.861635 | 0.794549 | 0.530962 |
| 42 | 0.698113 | 0.855346 | 0.802935 | 0.55523 |
| 43 | 0.691824 | 0.845912 | 0.794549 | 0.537736 |
| 44 | 0.710692 | 0.845912 | 0.800839 | 0.554046 |
| 45 | 0.679245 | 0.852201 | 0.794549 | 0.534907 |
| 46 | 0.704403 | 0.861635 | 0.809224 | 0.568778 |
| 47 | 0.666667 | 0.858491 | 0.794549 | 0.532236 |
| 48 | 0.679245 | 0.852201 | 0.794549 | 0.534907 |
| 49 | 0.704403 | 0.86478 | 0.811321 | 0.572888 |
| 50 | 0.761006 | 0.867925 | 0.832285 | 0.62511 |
| 51 | 0.704403 | 0.858491 | 0.807128 | 0.564694 |
| 52 | 0.704403 | 0.836478 | 0.792453 | 0.536808 |
| 53 | 0.685535 | 0.852201 | 0.796646 | 0.540339 |
| 54 | 0.685535 | 0.845912 | 0.792453 | 0.532289 |
| 55 | 0.704403 | 0.86478 | 0.811321 | 0.572888 |
| 56 | 0.685535 | 0.845912 | 0.792453 | 0.532289 |
| 57 | 0.679245 | 0.858491 | 0.798742 | 0.543079 |
| 58 | 0.704403 | 0.86478 | 0.811321 | 0.572888 |
| 59 | 0.679245 | 0.861635 | 0.800839 | 0.547205 |
| 60 | 0.704403 | 0.871069 | 0.815514 | 0.58119 |
| 61 | 0.679245 | 0.845912 | 0.790356 | 0.526837 |
| 62 | 0.716981 | 0.858491 | 0.811321 | 0.575472 |
| 63 | 0.698113 | 0.871069 | 0.813417 | 0.575838 |
| 64 | 0.72327 | 0.849057 | 0.807128 | 0.56885 |
| 65 | 0.641509 | 0.86478 | 0.790356 | 0.518869 |
| 66 | 0.716981 | 0.855346 | 0.809224 | 0.571435 |
| 67 | 0.654088 | 0.877358 | 0.802935 | 0.546802 |
| 68 | 0.704403 | 0.880503 | 0.821803 | 0.593852 |
| 69 | 0.672956 | 0.842767 | 0.786164 | 0.517373 |
| 70 | 0.685535 | 0.877358 | 0.813417 | 0.5736 |
| 71 | 0.691824 | 0.833333 | 0.786164 | 0.521967 |
| 72 | 0.698113 | 0.858491 | 0.805031 | 0.559298 |
| 73 | 0.679245 | 0.861635 | 0.800839 | 0.547205 |
| 74 | 0.679245 | 0.867925 | 0.805031 | 0.555539 |
| 75 | 0.666667 | 0.852201 | 0.790356 | 0.524023 |
| 76 | 0.691824 | 0.852201 | 0.798742 | 0.545766 |
| 77 | 0.672956 | 0.880503 | 0.811321 | 0.567211 |
| 78 | 0.685535 | 0.855346 | 0.798742 | 0.544402 |
| 79 | 0.679245 | 0.871069 | 0.807128 | 0.559749 |
| 80 | 0.710692 | 0.858491 | 0.809224 | 0.570085 |
| 81 | 0.716981 | 0.833333 | 0.794549 | 0.543856 |
| 82 | 0.679245 | 0.86478 | 0.802935 | 0.551358 |
| 83 | 0.716981 | 0.871069 | 0.819706 | 0.591879 |
| 84 | 0.710692 | 0.86478 | 0.813417 | 0.578258 |
| 85 | 0.666667 | 0.842767 | 0.784067 | 0.5119 |
| 86 | 0.698113 | 0.836478 | 0.790356 | 0.531343 |
| 87 | 0.72956 | 0.855346 | 0.813417 | 0.582217 |
| 88 | 0.704403 | 0.830189 | 0.78826 | 0.529054 |
| 89 | 0.716981 | 0.86478 | 0.815514 | 0.583622 |
| 90 | 0.72327 | 0.874214 | 0.823899 | 0.601374 |
| 91 | 0.698113 | 0.845912 | 0.796646 | 0.543177 |
| 92 | 0.710692 | 0.877358 | 0.821803 | 0.594926 |
| 93 | 0.698113 | 0.874214 | 0.815514 | 0.580042 |
| 94 | 0.710692 | 0.842767 | 0.798742 | 0.550097 |
| 95 | 0.704403 | 0.833333 | 0.790356 | 0.53292 |
| 96 | 0.666667 | 0.86478 | 0.798742 | 0.540557 |
| 97 | 0.679245 | 0.880503 | 0.813417 | 0.572552 |
| 98 | 0.691824 | 0.861635 | 0.805031 | 0.558002 |
| 99 | 0.698113 | 0.833333 | 0.78826 | 0.527446 |
| 100 | 0.704403 | 0.86478 | 0.811321 | 0.572888 |
| 101 | 0.704403 | 0.883648 | 0.823899 | 0.598131 |
| 102 | 0.691824 | 0.849057 | 0.796646 | 0.541738 |
| 103 | 0.698113 | 0.836478 | 0.790356 | 0.531343 |
| 104 | 0.672956 | 0.849057 | 0.790356 | 0.525411 |
| 105 | 0.685535 | 0.861635 | 0.802935 | 0.552607 |
| 106 | 0.698113 | 0.871069 | 0.813417 | 0.575838 |
| 107 | 0.672956 | 0.86478 | 0.800839 | 0.545961 |
| 108 | 0.704403 | 0.86478 | 0.811321 | 0.572888 |
| 109 | 0.710692 | 0.842767 | 0.798742 | 0.550097 |
| 110 | 0.710692 | 0.867925 | 0.815514 | 0.582383 |
| 111 | 0.710692 | 0.86478 | 0.813417 | 0.578258 |
| 112 | 0.710692 | 0.845912 | 0.800839 | 0.554046 |
| 113 | 0.698113 | 0.855346 | 0.802935 | 0.55523 |
| 114 | 0.710692 | 0.861635 | 0.811321 | 0.574158 |
| 115 | 0.691824 | 0.855346 | 0.800839 | 0.549819 |
| 116 | 0.691824 | 0.861635 | 0.805031 | 0.558002 |
| 117 | 0.685535 | 0.867925 | 0.807128 | 0.560919 |
| 118 | 0.704403 | 0.852201 | 0.802935 | 0.556604 |
| 119 | 0.704403 | 0.836478 | 0.792453 | 0.536808 |
| 120 | 0.679245 | 0.871069 | 0.807128 | 0.559749 |
| 121 | 0.672956 | 0.867925 | 0.802935 | 0.550153 |
| 122 | 0.685535 | 0.855346 | 0.798742 | 0.544402 |
| 123 | 0.698113 | 0.867925 | 0.811321 | 0.571662 |
| 124 | 0.691824 | 0.852201 | 0.798742 | 0.545766 |
| 125 | 0.698113 | 0.849057 | 0.798742 | 0.54717 |
| 126 | 0.704403 | 0.852201 | 0.802935 | 0.556604 |
| 127 | 0.685535 | 0.861635 | 0.802935 | 0.552607 |
| 128 | 0.698113 | 0.86478 | 0.809224 | 0.567514 |
| 129 | 0.691824 | 0.861635 | 0.805031 | 0.558002 |
| 130 | 0.704403 | 0.871069 | 0.815514 | 0.58119 |
| 131 | 0.72327 | 0.880503 | 0.828092 | 0.609773 |
| 132 | 0.666667 | 0.874214 | 0.805031 | 0.553252 |
| 133 | 0.704403 | 0.849057 | 0.800839 | 0.552596 |
| 134 | 0.685535 | 0.858491 | 0.800839 | 0.548491 |
| 135 | 0.761006 | 0.871069 | 0.834382 | 0.62917 |
| 136 | 0.672956 | 0.858491 | 0.796646 | 0.537661 |
| 137 | 0.698113 | 0.852201 | 0.800839 | 0.551187 |
| 138 | 0.698113 | 0.858491 | 0.805031 | 0.559298 |
| 139 | 0.685535 | 0.880503 | 0.815514 | 0.577886 |
| 140 | 0.685535 | 0.852201 | 0.796646 | 0.540339 |
| 141 | 0.704403 | 0.86478 | 0.811321 | 0.572888 |
| 142 | 0.716981 | 0.874214 | 0.821803 | 0.596048 |
| 143 | 0.691824 | 0.858491 | 0.802935 | 0.553897 |
| 144 | 0.710692 | 0.849057 | 0.802935 | 0.558018 |
| 145 | 0.716981 | 0.867925 | 0.81761 | 0.587737 |
| 146 | 0.704403 | 0.877358 | 0.819706 | 0.589603 |
| 147 | 0.704403 | 0.861635 | 0.809224 | 0.568778 |
| 148 | 0.685535 | 0.849057 | 0.794549 | 0.536302 |
| 149 | 0.685535 | 0.833333 | 0.784067 | 0.516483 |
| 150 | 0.672956 | 0.867925 | 0.802935 | 0.550153 |
| 151 | 0.691824 | 0.836478 | 0.78826 | 0.525874 |
| 152 | 0.710692 | 0.852201 | 0.805031 | 0.562016 |
| 153 | 0.72956 | 0.86478 | 0.819706 | 0.59434 |
| 154 | 0.72327 | 0.852201 | 0.809224 | 0.572827 |
| 155 | 0.704403 | 0.858491 | 0.807128 | 0.564694 |
| 156 | 0.748428 | 0.874214 | 0.832285 | 0.622642 |
| 157 | 0.698113 | 0.861635 | 0.807128 | 0.563393 |
| 158 | 0.691824 | 0.889937 | 0.823899 | 0.596216 |
| 159 | 0.716981 | 0.852201 | 0.807128 | 0.567423 |
| 160 | 0.698113 | 0.836478 | 0.790356 | 0.531343 |
| 161 | 0.685535 | 0.861635 | 0.802935 | 0.552607 |
| 162 | 0.691824 | 0.874214 | 0.813417 | 0.574696 |
| 163 | 0.685535 | 0.877358 | 0.813417 | 0.5736 |
| 164 | 0.685535 | 0.849057 | 0.794549 | 0.536302 |
| 165 | 0.716981 | 0.852201 | 0.807128 | 0.567423 |
| 166 | 0.704403 | 0.845912 | 0.798742 | 0.548614 |
| 167 | 0.672956 | 0.858491 | 0.796646 | 0.537661 |
| 168 | 0.710692 | 0.861635 | 0.811321 | 0.574158 |
| 169 | 0.716981 | 0.861635 | 0.813417 | 0.579534 |
| 170 | 0.72327 | 0.871069 | 0.821803 | 0.597217 |
| 171 | 0.685535 | 0.867925 | 0.807128 | 0.560919 |
| 172 | 0.679245 | 0.867925 | 0.805031 | 0.555539 |
| 173 | 0.685535 | 0.867925 | 0.807128 | 0.560919 |
| 174 | 0.698113 | 0.867925 | 0.811321 | 0.571662 |
| 175 | 0.72327 | 0.858491 | 0.813417 | 0.580854 |
| 176 | 0.742138 | 0.86478 | 0.823899 | 0.605042 |
| 177 | 0.735849 | 0.886792 | 0.836478 | 0.628828 |
| 178 | 0.704403 | 0.852201 | 0.802935 | 0.556604 |
| 179 | 0.698113 | 0.842767 | 0.794549 | 0.539209 |
| 180 | 0.679245 | 0.871069 | 0.807128 | 0.559749 |
| 181 | 0.716981 | 0.855346 | 0.809224 | 0.571435 |
| 182 | 0.710692 | 0.86478 | 0.813417 | 0.578258 |
| 183 | 0.72956 | 0.874214 | 0.825996 | 0.606696 |
| 184 | 0.704403 | 0.842767 | 0.796646 | 0.544655 |
| 185 | 0.704403 | 0.839623 | 0.794549 | 0.54072 |
| 186 | 0.716981 | 0.874214 | 0.821803 | 0.596048 |
| 187 | 0.698113 | 0.886792 | 0.823899 | 0.597148 |
| 188 | 0.691824 | 0.867925 | 0.809224 | 0.566293 |
| 189 | 0.698113 | 0.867925 | 0.811321 | 0.571662 |
| 190 | 0.672956 | 0.871069 | 0.805031 | 0.554373 |
| 191 | 0.691824 | 0.858491 | 0.802935 | 0.553897 |
| 192 | 0.685535 | 0.867925 | 0.807128 | 0.560919 |
| 193 | 0.679245 | 0.883648 | 0.815514 | 0.576879 |
| 194 | 0.72327 | 0.858491 | 0.813417 | 0.580854 |
| 195 | 0.704403 | 0.861635 | 0.809224 | 0.568778 |
| 196 | 0.704403 | 0.861635 | 0.809224 | 0.568778 |
| 197 | 0.685535 | 0.86478 | 0.805031 | 0.556749 |
| 198 | 0.698113 | 0.867925 | 0.811321 | 0.571662 |
| 199 | 0.698113 | 0.877358 | 0.81761 | 0.584274 |
| 200 | 0.742138 | 0.836478 | 0.805031 | 0.56952 |
| 201 | 0.710692 | 0.849057 | 0.802935 | 0.558018 |
| 202 | 0.710692 | 0.877358 | 0.821803 | 0.594926 |
| 203 | 0.748428 | 0.845912 | 0.813417 | 0.586561 |
| 204 | 0.716981 | 0.842767 | 0.800839 | 0.555534 |
| 205 | 0.72956 | 0.845912 | 0.807128 | 0.570318 |
| 206 | 0.716981 | 0.871069 | 0.819706 | 0.591879 |
| 207 | 0.716981 | 0.858491 | 0.811321 | 0.575472 |
| 208 | 0.748428 | 0.839623 | 0.809224 | 0.578806 |
| 209 | 0.698113 | 0.861635 | 0.807128 | 0.563393 |
| 210 | 0.710692 | 0.858491 | 0.809224 | 0.570085 |
| 211 | 0.691824 | 0.867925 | 0.809224 | 0.566293 |
| 212 | 0.72956 | 0.871069 | 0.823899 | 0.602551 |
| 213 | 0.704403 | 0.86478 | 0.811321 | 0.572888 |
| 214 | 0.704403 | 0.874214 | 0.81761 | 0.585382 |
| 215 | 0.710692 | 0.874214 | 0.819706 | 0.590717 |
| 216 | 0.704403 | 0.855346 | 0.805031 | 0.560636 |
| 217 | 0.698113 | 0.880503 | 0.819706 | 0.588536 |
| 218 | 0.735849 | 0.867925 | 0.823899 | 0.603774 |
| 219 | 0.72956 | 0.86478 | 0.819706 | 0.59434 |
| 220 | 0.710692 | 0.86478 | 0.813417 | 0.578258 |
| 221 | 0.691824 | 0.849057 | 0.796646 | 0.541738 |
| 222 | 0.628931 | 0.836478 | 0.767296 | 0.470851 |
| 223 | 0.710692 | 0.861635 | 0.811321 | 0.574158 |
| 224 | 0.742138 | 0.86478 | 0.823899 | 0.605042 |
| 225 | 0.672956 | 0.86478 | 0.800839 | 0.545961 |
| 226 | 0.72956 | 0.861635 | 0.81761 | 0.590273 |
| 227 | 0.698113 | 0.867925 | 0.811321 | 0.571662 |
| 228 | 0.72956 | 0.867925 | 0.821803 | 0.598432 |
| 229 | 0.72956 | 0.842767 | 0.805031 | 0.566399 |
| 230 | 0.710692 | 0.852201 | 0.805031 | 0.562016 |
| 231 | 0.691824 | 0.858491 | 0.802935 | 0.553897 |
| 232 | 0.72956 | 0.861635 | 0.81761 | 0.590273 |
| 233 | 0.72327 | 0.855346 | 0.811321 | 0.576828 |
| 234 | 0.666667 | 0.845912 | 0.786164 | 0.515916 |
| 235 | 0.710692 | 0.861635 | 0.811321 | 0.574158 |
| 236 | 0.716981 | 0.867925 | 0.81761 | 0.587737 |
| 237 | 0.685535 | 0.842767 | 0.790356 | 0.528302 |
| 238 | 0.710692 | 0.874214 | 0.819706 | 0.590717 |
| 239 | 0.72956 | 0.855346 | 0.813417 | 0.582217 |
| 240 | 0.72327 | 0.86478 | 0.81761 | 0.588983 |
| 241 | 0.716981 | 0.880503 | 0.825996 | 0.604471 |
| 242 | 0.710692 | 0.852201 | 0.805031 | 0.562016 |
| 243 | 0.716981 | 0.855346 | 0.809224 | 0.571435 |
| 244 | 0.742138 | 0.861635 | 0.821803 | 0.600999 |
| 245 | 0.710692 | 0.880503 | 0.823899 | 0.599164 |
| 246 | 0.710692 | 0.874214 | 0.819706 | 0.590717 |
| 247 | 0.691824 | 0.871069 | 0.811321 | 0.57048 |
| 248 | 0.72956 | 0.836478 | 0.800839 | 0.558629 |
| 249 | 0.710692 | 0.867925 | 0.815514 | 0.582383 |
| 250 | 0.72327 | 0.874214 | 0.823899 | 0.601374 |
| 251 | 0.691824 | 0.886792 | 0.821803 | 0.591851 |
| 252 | 0.716981 | 0.855346 | 0.809224 | 0.571435 |
| 253 | 0.710692 | 0.855346 | 0.807128 | 0.566038 |
| 254 | 0.704403 | 0.877358 | 0.819706 | 0.589603 |
| 255 | 0.710692 | 0.849057 | 0.802935 | 0.558018 |
| 256 | 0.735849 | 0.880503 | 0.832285 | 0.620365 |
| 257 | 0.710692 | 0.871069 | 0.81761 | 0.586537 |
| 258 | 0.742138 | 0.849057 | 0.813417 | 0.585071 |
| 259 | 0.698113 | 0.858491 | 0.805031 | 0.559298 |
| 260 | 0.641509 | 0.877358 | 0.798742 | 0.536031 |
| 261 | 0.672956 | 0.852201 | 0.792453 | 0.529468 |
| 262 | 0.72327 | 0.867925 | 0.819706 | 0.593086 |
| 263 | 0.716981 | 0.877358 | 0.823899 | 0.600245 |
| 264 | 0.72327 | 0.845912 | 0.805031 | 0.564897 |
| 265 | 0.698113 | 0.90566 | 0.836478 | 0.623745 |
| 266 | 0.704403 | 0.836478 | 0.792453 | 0.536808 |
| 267 | 0.72327 | 0.849057 | 0.807128 | 0.56885 |
| 268 | 0.710692 | 0.858491 | 0.809224 | 0.570085 |
| 269 | 0.735849 | 0.867925 | 0.823899 | 0.603774 |
| 270 | 0.685535 | 0.839623 | 0.78826 | 0.524339 |
| 271 | 0.710692 | 0.858491 | 0.809224 | 0.570085 |
| 272 | 0.72956 | 0.861635 | 0.81761 | 0.590273 |
| 273 | 0.748428 | 0.861635 | 0.823899 | 0.606357 |
| 274 | 0.710692 | 0.849057 | 0.802935 | 0.558018 |
| 275 | 0.691824 | 0.871069 | 0.811321 | 0.57048 |
| 276 | 0.72956 | 0.836478 | 0.800839 | 0.558629 |
| 277 | 0.704403 | 0.867925 | 0.813417 | 0.577025 |
| 278 | 0.698113 | 0.861635 | 0.807128 | 0.563393 |
| 279 | 0.710692 | 0.886792 | 0.828092 | 0.607727 |
| 280 | 0.679245 | 0.871069 | 0.807128 | 0.559749 |
| 281 | 0.685535 | 0.86478 | 0.805031 | 0.556749 |
| 282 | 0.748428 | 0.852201 | 0.81761 | 0.594407 |
| 283 | 0.704403 | 0.883648 | 0.823899 | 0.598131 |
| 284 | 0.72956 | 0.883648 | 0.832285 | 0.6193 |
| 285 | 0.698113 | 0.883648 | 0.821803 | 0.592827 |
| 286 | 0.710692 | 0.836478 | 0.794549 | 0.542269 |
| 287 | 0.716981 | 0.852201 | 0.807128 | 0.567423 |
| 288 | 0.72327 | 0.855346 | 0.811321 | 0.576828 |
| 289 | 0.691824 | 0.880503 | 0.81761 | 0.583214 |
| 290 | 0.698113 | 0.86478 | 0.809224 | 0.567514 |
| 291 | 0.710692 | 0.842767 | 0.798742 | 0.550097 |
| 292 | 0.735849 | 0.849057 | 0.811321 | 0.579667 |
| 293 | 0.716981 | 0.861635 | 0.813417 | 0.579534 |
| 294 | 0.698113 | 0.880503 | 0.819706 | 0.588536 |
| 295 | 0.666667 | 0.852201 | 0.790356 | 0.524023 |
| 296 | 0.72956 | 0.883648 | 0.832285 | 0.6193 |
| 297 | 0.685535 | 0.858491 | 0.800839 | 0.548491 |
| 298 | 0.710692 | 0.858491 | 0.809224 | 0.570085 |
| 299 | 0.698113 | 0.861635 | 0.807128 | 0.563393 |
| 300 | 0.72956 | 0.871069 | 0.823899 | 0.602551 |
| 301 | 0.685535 | 0.871069 | 0.809224 | 0.565118 |
| 302 | 0.704403 | 0.86478 | 0.811321 | 0.572888 |
| 303 | 0.748428 | 0.855346 | 0.819706 | 0.598366 |
| 304 | 0.710692 | 0.858491 | 0.809224 | 0.570085 |
| 305 | 0.716981 | 0.880503 | 0.825996 | 0.604471 |
| 306 | 0.72956 | 0.855346 | 0.813417 | 0.582217 |
| 307 | 0.72956 | 0.845912 | 0.807128 | 0.570318 |
| 308 | 0.710692 | 0.845912 | 0.800839 | 0.554046 |
| 309 | 0.685535 | 0.86478 | 0.805031 | 0.556749 |
| 310 | 0.72956 | 0.852201 | 0.811321 | 0.578227 |
| 311 | 0.704403 | 0.880503 | 0.821803 | 0.593852 |
| 312 | 0.685535 | 0.871069 | 0.809224 | 0.565118 |
| 313 | 0.761006 | 0.852201 | 0.821803 | 0.605182 |
| 314 | 0.716981 | 0.855346 | 0.809224 | 0.571435 |
| 315 | 0.72327 | 0.858491 | 0.813417 | 0.580854 |
| 316 | 0.704403 | 0.886792 | 0.825996 | 0.60244 |
| 317 | 0.72327 | 0.852201 | 0.809224 | 0.572827 |
| 318 | 0.698113 | 0.855346 | 0.802935 | 0.55523 |
| 319 | 0.710692 | 0.86478 | 0.813417 | 0.578258 |
| 320 | 0.704403 | 0.867925 | 0.813417 | 0.577025 |
| 321 | 0.685535 | 0.855346 | 0.798742 | 0.544402 |

1. Results for SMO

| **Number of features** | **SN** | **SP** | **ACC** | **MCC** |
| --- | --- | --- | --- | --- |
| 4 | 0.327044 | 0.933962 | 0.731656 | 0.341752 |
| 5 | 0.308176 | 0.959119 | 0.742138 | 0.3747 |
| 6 | 0.308176 | 0.959119 | 0.742138 | 0.3747 |
| 7 | 0.308176 | 0.959119 | 0.742138 | 0.3747 |
| 8 | 0.308176 | 0.959119 | 0.742138 | 0.3747 |
| 9 | 0.314465 | 0.937107 | 0.72956 | 0.335142 |
| 10 | 0.314465 | 0.937107 | 0.72956 | 0.335142 |
| 11 | 0.314465 | 0.940252 | 0.731656 | 0.341362 |
| 12 | 0.320755 | 0.921384 | 0.721174 | 0.311887 |
| 13 | 0.314465 | 0.924528 | 0.721174 | 0.311193 |
| 14 | 0.314465 | 0.924528 | 0.721174 | 0.311193 |
| 15 | 0.314465 | 0.930818 | 0.725367 | 0.322988 |
| 16 | 0.314465 | 0.930818 | 0.725367 | 0.322988 |
| 17 | 0.314465 | 0.930818 | 0.725367 | 0.322988 |
| 18 | 0.314465 | 0.937107 | 0.72956 | 0.335142 |
| 19 | 0.314465 | 0.940252 | 0.731656 | 0.341362 |
| 20 | 0.314465 | 0.933962 | 0.727463 | 0.329018 |
| 21 | 0.36478 | 0.921384 | 0.735849 | 0.355827 |
| 22 | 0.345912 | 0.924528 | 0.731656 | 0.342948 |
| 23 | 0.54717 | 0.842767 | 0.744235 | 0.406263 |
| 24 | 0.54717 | 0.842767 | 0.744235 | 0.406263 |
| 25 | 0.54717 | 0.842767 | 0.744235 | 0.406263 |
| 26 | 0.54717 | 0.842767 | 0.744235 | 0.406263 |
| 27 | 0.54717 | 0.842767 | 0.744235 | 0.406263 |
| 28 | 0.54717 | 0.842767 | 0.744235 | 0.406263 |
| 29 | 0.528302 | 0.858491 | 0.748428 | 0.410493 |
| 30 | 0.528302 | 0.858491 | 0.748428 | 0.410493 |
| 31 | 0.528302 | 0.858491 | 0.748428 | 0.410493 |
| 32 | 0.528302 | 0.858491 | 0.748428 | 0.410493 |
| 33 | 0.528302 | 0.858491 | 0.748428 | 0.410493 |
| 34 | 0.528302 | 0.858491 | 0.748428 | 0.410493 |
| 35 | 0.534591 | 0.845912 | 0.742138 | 0.39909 |
| 36 | 0.534591 | 0.845912 | 0.742138 | 0.39909 |
| 37 | 0.522013 | 0.845912 | 0.737945 | 0.387681 |
| 38 | 0.522013 | 0.845912 | 0.737945 | 0.387681 |
| 39 | 0.515723 | 0.849057 | 0.737945 | 0.386195 |
| 40 | 0.515723 | 0.849057 | 0.737945 | 0.386195 |
| 41 | 0.515723 | 0.849057 | 0.737945 | 0.386195 |
| 42 | 0.553459 | 0.852201 | 0.752621 | 0.424517 |
| 43 | 0.540881 | 0.855346 | 0.750524 | 0.417502 |
| 44 | 0.540881 | 0.855346 | 0.750524 | 0.417502 |
| 45 | 0.540881 | 0.855346 | 0.750524 | 0.417502 |
| 46 | 0.528302 | 0.858491 | 0.748428 | 0.410493 |
| 47 | 0.540881 | 0.855346 | 0.750524 | 0.417502 |
| 48 | 0.528302 | 0.852201 | 0.744235 | 0.40188 |
| 49 | 0.528302 | 0.852201 | 0.744235 | 0.40188 |
| 50 | 0.528302 | 0.849057 | 0.742138 | 0.397621 |
| 51 | 0.522013 | 0.849057 | 0.740042 | 0.391916 |
| 52 | 0.522013 | 0.849057 | 0.740042 | 0.391916 |
| 53 | 0.522013 | 0.849057 | 0.740042 | 0.391916 |
| 54 | 0.522013 | 0.849057 | 0.740042 | 0.391916 |
| 55 | 0.522013 | 0.849057 | 0.740042 | 0.391916 |
| 56 | 0.534591 | 0.849057 | 0.744235 | 0.403311 |
| 57 | 0.522013 | 0.852201 | 0.742138 | 0.396183 |
| 58 | 0.522013 | 0.852201 | 0.742138 | 0.396183 |
| 59 | 0.522013 | 0.852201 | 0.742138 | 0.396183 |
| 60 | 0.522013 | 0.852201 | 0.742138 | 0.396183 |
| 61 | 0.528302 | 0.858491 | 0.748428 | 0.410493 |
| 62 | 0.528302 | 0.858491 | 0.748428 | 0.410493 |
| 63 | 0.528302 | 0.858491 | 0.748428 | 0.410493 |
| 64 | 0.528302 | 0.858491 | 0.748428 | 0.410493 |
| 65 | 0.528302 | 0.858491 | 0.748428 | 0.410493 |
| 66 | 0.528302 | 0.858491 | 0.748428 | 0.410493 |
| 67 | 0.528302 | 0.858491 | 0.748428 | 0.410493 |
| 68 | 0.515723 | 0.861635 | 0.746331 | 0.403485 |
| 69 | 0.515723 | 0.861635 | 0.746331 | 0.403485 |
| 70 | 0.515723 | 0.861635 | 0.746331 | 0.403485 |
| 71 | 0.515723 | 0.858491 | 0.744235 | 0.399113 |
| 72 | 0.515723 | 0.858491 | 0.744235 | 0.399113 |
| 73 | 0.515723 | 0.858491 | 0.744235 | 0.399113 |
| 74 | 0.515723 | 0.861635 | 0.746331 | 0.403485 |
| 75 | 0.515723 | 0.858491 | 0.744235 | 0.399113 |
| 76 | 0.515723 | 0.861635 | 0.746331 | 0.403485 |
| 77 | 0.515723 | 0.861635 | 0.746331 | 0.403485 |
| 78 | 0.515723 | 0.861635 | 0.746331 | 0.403485 |
| 79 | 0.515723 | 0.861635 | 0.746331 | 0.403485 |
| 80 | 0.515723 | 0.861635 | 0.746331 | 0.403485 |
| 81 | 0.515723 | 0.861635 | 0.746331 | 0.403485 |
| 82 | 0.509434 | 0.86478 | 0.746331 | 0.402192 |
| 83 | 0.509434 | 0.86478 | 0.746331 | 0.402192 |
| 84 | 0.509434 | 0.86478 | 0.746331 | 0.402192 |
| 85 | 0.509434 | 0.867925 | 0.748428 | 0.406642 |
| 86 | 0.509434 | 0.867925 | 0.748428 | 0.406642 |
| 87 | 0.509434 | 0.867925 | 0.748428 | 0.406642 |
| 88 | 0.509434 | 0.871069 | 0.750524 | 0.411127 |
| 89 | 0.509434 | 0.867925 | 0.748428 | 0.406642 |
| 90 | 0.509434 | 0.867925 | 0.748428 | 0.406642 |
| 91 | 0.509434 | 0.867925 | 0.748428 | 0.406642 |
| 92 | 0.509434 | 0.867925 | 0.748428 | 0.406642 |
| 93 | 0.509434 | 0.867925 | 0.748428 | 0.406642 |
| 94 | 0.509434 | 0.867925 | 0.748428 | 0.406642 |
| 95 | 0.496855 | 0.880503 | 0.752621 | 0.413449 |
| 96 | 0.496855 | 0.880503 | 0.752621 | 0.413449 |
| 97 | 0.496855 | 0.880503 | 0.752621 | 0.413449 |
| 98 | 0.503145 | 0.883648 | 0.756813 | 0.423785 |
| 99 | 0.503145 | 0.883648 | 0.756813 | 0.423785 |
| 100 | 0.503145 | 0.883648 | 0.756813 | 0.423785 |
| 101 | 0.503145 | 0.883648 | 0.756813 | 0.423785 |
| 102 | 0.503145 | 0.883648 | 0.756813 | 0.423785 |
| 103 | 0.503145 | 0.883648 | 0.756813 | 0.423785 |
| 104 | 0.503145 | 0.883648 | 0.756813 | 0.423785 |
| 105 | 0.484277 | 0.883648 | 0.750524 | 0.406692 |
| 106 | 0.484277 | 0.883648 | 0.750524 | 0.406692 |
| 107 | 0.503145 | 0.883648 | 0.756813 | 0.423785 |
| 108 | 0.503145 | 0.883648 | 0.756813 | 0.423785 |
| 109 | 0.503145 | 0.883648 | 0.756813 | 0.423785 |
| 110 | 0.503145 | 0.883648 | 0.756813 | 0.423785 |
| 111 | 0.503145 | 0.883648 | 0.756813 | 0.423785 |
| 112 | 0.503145 | 0.883648 | 0.756813 | 0.423785 |
| 113 | 0.503145 | 0.883648 | 0.756813 | 0.423785 |
| 114 | 0.503145 | 0.883648 | 0.756813 | 0.423785 |
| 115 | 0.503145 | 0.883648 | 0.756813 | 0.423785 |
| 116 | 0.503145 | 0.883648 | 0.756813 | 0.423785 |
| 117 | 0.503145 | 0.883648 | 0.756813 | 0.423785 |
| 118 | 0.503145 | 0.883648 | 0.756813 | 0.423785 |
| 119 | 0.496855 | 0.883648 | 0.754717 | 0.418107 |
| 120 | 0.503145 | 0.883648 | 0.756813 | 0.423785 |
| 121 | 0.503145 | 0.883648 | 0.756813 | 0.423785 |
| 122 | 0.503145 | 0.883648 | 0.756813 | 0.423785 |
| 123 | 0.503145 | 0.883648 | 0.756813 | 0.423785 |
| 124 | 0.503145 | 0.883648 | 0.756813 | 0.423785 |
| 125 | 0.503145 | 0.883648 | 0.756813 | 0.423785 |
| 126 | 0.503145 | 0.883648 | 0.756813 | 0.423785 |
| 127 | 0.484277 | 0.883648 | 0.750524 | 0.406692 |
| 128 | 0.471698 | 0.883648 | 0.746331 | 0.395192 |
| 129 | 0.471698 | 0.883648 | 0.746331 | 0.395192 |
| 130 | 0.471698 | 0.883648 | 0.746331 | 0.395192 |
| 131 | 0.471698 | 0.883648 | 0.746331 | 0.395192 |
| 132 | 0.471698 | 0.883648 | 0.746331 | 0.395192 |
| 133 | 0.471698 | 0.883648 | 0.746331 | 0.395192 |
| 134 | 0.471698 | 0.883648 | 0.746331 | 0.395192 |
| 135 | 0.465409 | 0.883648 | 0.744235 | 0.389409 |
| 136 | 0.465409 | 0.883648 | 0.744235 | 0.389409 |
| 137 | 0.465409 | 0.883648 | 0.744235 | 0.389409 |
| 138 | 0.465409 | 0.883648 | 0.744235 | 0.389409 |
| 139 | 0.465409 | 0.883648 | 0.744235 | 0.389409 |
| 140 | 0.465409 | 0.883648 | 0.744235 | 0.389409 |
| 141 | 0.465409 | 0.883648 | 0.744235 | 0.389409 |
| 142 | 0.471698 | 0.883648 | 0.746331 | 0.395192 |
| 143 | 0.471698 | 0.883648 | 0.746331 | 0.395192 |
| 144 | 0.471698 | 0.883648 | 0.746331 | 0.395192 |
| 145 | 0.471698 | 0.883648 | 0.746331 | 0.395192 |
| 146 | 0.471698 | 0.883648 | 0.746331 | 0.395192 |
| 147 | 0.471698 | 0.883648 | 0.746331 | 0.395192 |
| 148 | 0.471698 | 0.883648 | 0.746331 | 0.395192 |
| 149 | 0.471698 | 0.883648 | 0.746331 | 0.395192 |
| 150 | 0.471698 | 0.883648 | 0.746331 | 0.395192 |
| 151 | 0.471698 | 0.883648 | 0.746331 | 0.395192 |
| 152 | 0.471698 | 0.883648 | 0.746331 | 0.395192 |
| 153 | 0.471698 | 0.883648 | 0.746331 | 0.395192 |
| 154 | 0.471698 | 0.883648 | 0.746331 | 0.395192 |
| 155 | 0.471698 | 0.883648 | 0.746331 | 0.395192 |
| 156 | 0.471698 | 0.883648 | 0.746331 | 0.395192 |
| 157 | 0.471698 | 0.883648 | 0.746331 | 0.395192 |
| 158 | 0.471698 | 0.883648 | 0.746331 | 0.395192 |
| 159 | 0.471698 | 0.883648 | 0.746331 | 0.395192 |
| 160 | 0.471698 | 0.883648 | 0.746331 | 0.395192 |
| 161 | 0.471698 | 0.883648 | 0.746331 | 0.395192 |
| 162 | 0.471698 | 0.883648 | 0.746331 | 0.395192 |
| 163 | 0.471698 | 0.883648 | 0.746331 | 0.395192 |
| 164 | 0.471698 | 0.883648 | 0.746331 | 0.395192 |
| 165 | 0.471698 | 0.883648 | 0.746331 | 0.395192 |
| 166 | 0.471698 | 0.883648 | 0.746331 | 0.395192 |
| 167 | 0.496855 | 0.883648 | 0.754717 | 0.418107 |
| 168 | 0.503145 | 0.883648 | 0.756813 | 0.423785 |
| 169 | 0.503145 | 0.883648 | 0.756813 | 0.423785 |
| 170 | 0.503145 | 0.883648 | 0.756813 | 0.423785 |
| 171 | 0.496855 | 0.883648 | 0.754717 | 0.418107 |
| 172 | 0.496855 | 0.883648 | 0.754717 | 0.418107 |
| 173 | 0.496855 | 0.883648 | 0.754717 | 0.418107 |
| 174 | 0.503145 | 0.883648 | 0.756813 | 0.423785 |
| 175 | 0.503145 | 0.883648 | 0.756813 | 0.423785 |
| 176 | 0.503145 | 0.883648 | 0.756813 | 0.423785 |
| 177 | 0.503145 | 0.883648 | 0.756813 | 0.423785 |
| 178 | 0.496855 | 0.883648 | 0.754717 | 0.418107 |
| 179 | 0.496855 | 0.883648 | 0.754717 | 0.418107 |
| 180 | 0.496855 | 0.883648 | 0.754717 | 0.418107 |
| 181 | 0.496855 | 0.883648 | 0.754717 | 0.418107 |
| 182 | 0.503145 | 0.883648 | 0.756813 | 0.423785 |
| 183 | 0.503145 | 0.883648 | 0.756813 | 0.423785 |
| 184 | 0.503145 | 0.883648 | 0.756813 | 0.423785 |
| 185 | 0.503145 | 0.883648 | 0.756813 | 0.423785 |
| 186 | 0.503145 | 0.883648 | 0.756813 | 0.423785 |
| 187 | 0.503145 | 0.883648 | 0.756813 | 0.423785 |
| 188 | 0.503145 | 0.883648 | 0.756813 | 0.423785 |
| 189 | 0.503145 | 0.883648 | 0.756813 | 0.423785 |
| 190 | 0.503145 | 0.883648 | 0.756813 | 0.423785 |
| 191 | 0.503145 | 0.883648 | 0.756813 | 0.423785 |
| 192 | 0.503145 | 0.883648 | 0.756813 | 0.423785 |
| 193 | 0.503145 | 0.883648 | 0.756813 | 0.423785 |
| 194 | 0.503145 | 0.883648 | 0.756813 | 0.423785 |
| 195 | 0.503145 | 0.883648 | 0.756813 | 0.423785 |
| 196 | 0.503145 | 0.883648 | 0.756813 | 0.423785 |
| 197 | 0.503145 | 0.883648 | 0.756813 | 0.423785 |
| 198 | 0.503145 | 0.883648 | 0.756813 | 0.423785 |
| 199 | 0.503145 | 0.883648 | 0.756813 | 0.423785 |
| 200 | 0.503145 | 0.883648 | 0.756813 | 0.423785 |
| 201 | 0.503145 | 0.883648 | 0.756813 | 0.423785 |
| 202 | 0.503145 | 0.883648 | 0.756813 | 0.423785 |
| 203 | 0.503145 | 0.883648 | 0.756813 | 0.423785 |
| 204 | 0.503145 | 0.883648 | 0.756813 | 0.423785 |
| 205 | 0.503145 | 0.883648 | 0.756813 | 0.423785 |
| 206 | 0.503145 | 0.883648 | 0.756813 | 0.423785 |
| 207 | 0.503145 | 0.883648 | 0.756813 | 0.423785 |
| 208 | 0.503145 | 0.883648 | 0.756813 | 0.423785 |
| 209 | 0.503145 | 0.883648 | 0.756813 | 0.423785 |
| 210 | 0.503145 | 0.883648 | 0.756813 | 0.423785 |
| 211 | 0.503145 | 0.883648 | 0.756813 | 0.423785 |
| 212 | 0.503145 | 0.883648 | 0.756813 | 0.423785 |
| 213 | 0.503145 | 0.883648 | 0.756813 | 0.423785 |
| 214 | 0.503145 | 0.883648 | 0.756813 | 0.423785 |
| 215 | 0.503145 | 0.883648 | 0.756813 | 0.423785 |
| 216 | 0.503145 | 0.883648 | 0.756813 | 0.423785 |
| 217 | 0.503145 | 0.883648 | 0.756813 | 0.423785 |
| 218 | 0.503145 | 0.883648 | 0.756813 | 0.423785 |
| 219 | 0.503145 | 0.883648 | 0.756813 | 0.423785 |
| 220 | 0.503145 | 0.883648 | 0.756813 | 0.423785 |
| 221 | 0.503145 | 0.883648 | 0.756813 | 0.423785 |
| 222 | 0.503145 | 0.883648 | 0.756813 | 0.423785 |
| 223 | 0.503145 | 0.883648 | 0.756813 | 0.423785 |
| 224 | 0.503145 | 0.883648 | 0.756813 | 0.423785 |
| 225 | 0.503145 | 0.883648 | 0.756813 | 0.423785 |
| 226 | 0.503145 | 0.883648 | 0.756813 | 0.423785 |
| 227 | 0.503145 | 0.883648 | 0.756813 | 0.423785 |
| 228 | 0.503145 | 0.883648 | 0.756813 | 0.423785 |
| 229 | 0.503145 | 0.883648 | 0.756813 | 0.423785 |
| 230 | 0.503145 | 0.883648 | 0.756813 | 0.423785 |
| 231 | 0.503145 | 0.883648 | 0.756813 | 0.423785 |
| 232 | 0.503145 | 0.883648 | 0.756813 | 0.423785 |
| 233 | 0.503145 | 0.883648 | 0.756813 | 0.423785 |
| 234 | 0.503145 | 0.883648 | 0.756813 | 0.423785 |
| 235 | 0.503145 | 0.883648 | 0.756813 | 0.423785 |
| 236 | 0.503145 | 0.883648 | 0.756813 | 0.423785 |
| 237 | 0.503145 | 0.883648 | 0.756813 | 0.423785 |
| 238 | 0.503145 | 0.883648 | 0.756813 | 0.423785 |
| 239 | 0.503145 | 0.883648 | 0.756813 | 0.423785 |
| 240 | 0.503145 | 0.883648 | 0.756813 | 0.423785 |
| 241 | 0.503145 | 0.883648 | 0.756813 | 0.423785 |
| 242 | 0.503145 | 0.883648 | 0.756813 | 0.423785 |
| 243 | 0.503145 | 0.883648 | 0.756813 | 0.423785 |
| 244 | 0.503145 | 0.883648 | 0.756813 | 0.423785 |
| 245 | 0.503145 | 0.883648 | 0.756813 | 0.423785 |
| 246 | 0.503145 | 0.883648 | 0.756813 | 0.423785 |
| 247 | 0.503145 | 0.883648 | 0.756813 | 0.423785 |
| 248 | 0.503145 | 0.883648 | 0.756813 | 0.423785 |
| 249 | 0.503145 | 0.883648 | 0.756813 | 0.423785 |
| 250 | 0.503145 | 0.883648 | 0.756813 | 0.423785 |
| 251 | 0.503145 | 0.883648 | 0.756813 | 0.423785 |
| 252 | 0.503145 | 0.883648 | 0.756813 | 0.423785 |
| 253 | 0.503145 | 0.883648 | 0.756813 | 0.423785 |
| 254 | 0.503145 | 0.883648 | 0.756813 | 0.423785 |
| 255 | 0.503145 | 0.883648 | 0.756813 | 0.423785 |
| 256 | 0.503145 | 0.883648 | 0.756813 | 0.423785 |
| 257 | 0.503145 | 0.883648 | 0.756813 | 0.423785 |
| 258 | 0.503145 | 0.883648 | 0.756813 | 0.423785 |
| 259 | 0.503145 | 0.883648 | 0.756813 | 0.423785 |
| 260 | 0.503145 | 0.883648 | 0.756813 | 0.423785 |
| 261 | 0.503145 | 0.883648 | 0.756813 | 0.423785 |
| 262 | 0.503145 | 0.883648 | 0.756813 | 0.423785 |
| 263 | 0.503145 | 0.883648 | 0.756813 | 0.423785 |
| 264 | 0.503145 | 0.883648 | 0.756813 | 0.423785 |
| 265 | 0.503145 | 0.883648 | 0.756813 | 0.423785 |
| 266 | 0.503145 | 0.883648 | 0.756813 | 0.423785 |
| 267 | 0.503145 | 0.883648 | 0.756813 | 0.423785 |
| 268 | 0.503145 | 0.883648 | 0.756813 | 0.423785 |
| 269 | 0.503145 | 0.883648 | 0.756813 | 0.423785 |
| 270 | 0.503145 | 0.883648 | 0.756813 | 0.423785 |
| 271 | 0.503145 | 0.883648 | 0.756813 | 0.423785 |
| 272 | 0.503145 | 0.883648 | 0.756813 | 0.423785 |
| 273 | 0.503145 | 0.883648 | 0.756813 | 0.423785 |
| 274 | 0.503145 | 0.883648 | 0.756813 | 0.423785 |
| 275 | 0.503145 | 0.883648 | 0.756813 | 0.423785 |
| 276 | 0.503145 | 0.883648 | 0.756813 | 0.423785 |
| 277 | 0.503145 | 0.883648 | 0.756813 | 0.423785 |
| 278 | 0.503145 | 0.883648 | 0.756813 | 0.423785 |
| 279 | 0.503145 | 0.883648 | 0.756813 | 0.423785 |
| 280 | 0.503145 | 0.883648 | 0.756813 | 0.423785 |
| 281 | 0.503145 | 0.883648 | 0.756813 | 0.423785 |
| 282 | 0.503145 | 0.883648 | 0.756813 | 0.423785 |
| 283 | 0.503145 | 0.883648 | 0.756813 | 0.423785 |
| 284 | 0.503145 | 0.883648 | 0.756813 | 0.423785 |
| 285 | 0.503145 | 0.883648 | 0.756813 | 0.423785 |
| 286 | 0.503145 | 0.883648 | 0.756813 | 0.423785 |
| 287 | 0.503145 | 0.883648 | 0.756813 | 0.423785 |
| 288 | 0.503145 | 0.883648 | 0.756813 | 0.423785 |
| 289 | 0.503145 | 0.883648 | 0.756813 | 0.423785 |
| 290 | 0.503145 | 0.883648 | 0.756813 | 0.423785 |
| 291 | 0.503145 | 0.883648 | 0.756813 | 0.423785 |
| 292 | 0.503145 | 0.883648 | 0.756813 | 0.423785 |
| 293 | 0.503145 | 0.883648 | 0.756813 | 0.423785 |
| 294 | 0.503145 | 0.883648 | 0.756813 | 0.423785 |
| 295 | 0.503145 | 0.883648 | 0.756813 | 0.423785 |
| 296 | 0.503145 | 0.883648 | 0.756813 | 0.423785 |
| 297 | 0.503145 | 0.883648 | 0.756813 | 0.423785 |
| 298 | 0.503145 | 0.883648 | 0.756813 | 0.423785 |
| 299 | 0.503145 | 0.883648 | 0.756813 | 0.423785 |
| 300 | 0.503145 | 0.883648 | 0.756813 | 0.423785 |
| 301 | 0.503145 | 0.883648 | 0.756813 | 0.423785 |
| 302 | 0.503145 | 0.883648 | 0.756813 | 0.423785 |
| 303 | 0.503145 | 0.883648 | 0.756813 | 0.423785 |
| 304 | 0.503145 | 0.883648 | 0.756813 | 0.423785 |
| 305 | 0.503145 | 0.883648 | 0.756813 | 0.423785 |
| 306 | 0.503145 | 0.883648 | 0.756813 | 0.423785 |
| 307 | 0.503145 | 0.883648 | 0.756813 | 0.423785 |
| 308 | 0.503145 | 0.883648 | 0.756813 | 0.423785 |
| 309 | 0.503145 | 0.883648 | 0.756813 | 0.423785 |
| 310 | 0.503145 | 0.883648 | 0.756813 | 0.423785 |
| 311 | 0.503145 | 0.883648 | 0.756813 | 0.423785 |
| 312 | 0.503145 | 0.883648 | 0.756813 | 0.423785 |
| 313 | 0.503145 | 0.883648 | 0.756813 | 0.423785 |
| 314 | 0.503145 | 0.883648 | 0.756813 | 0.423785 |
| 315 | 0.503145 | 0.883648 | 0.756813 | 0.423785 |
| 316 | 0.503145 | 0.883648 | 0.756813 | 0.423785 |
| 317 | 0.503145 | 0.883648 | 0.756813 | 0.423785 |
| 318 | 0.503145 | 0.883648 | 0.756813 | 0.423785 |
| 319 | 0.503145 | 0.883648 | 0.756813 | 0.423785 |
| 320 | 0.503145 | 0.883648 | 0.756813 | 0.423785 |
| 321 | 0.503145 | 0.883648 | 0.756813 | 0.423785 |

1. Results for Dagging

| **Number of features** | **SN** | **SP** | **ACC** | **MCC** |
| --- | --- | --- | --- | --- |
| 4 | 0.113208 | 0.977987 | 0.689727 | 0.192905 |
| 5 | 0.207547 | 0.965409 | 0.712788 | 0.281759 |
| 6 | 0.245283 | 0.962264 | 0.72327 | 0.316621 |
| 7 | 0.27044 | 0.968553 | 0.735849 | 0.358491 |
| 8 | 0.226415 | 0.959119 | 0.714885 | 0.288083 |
| 9 | 0.251572 | 0.962264 | 0.725367 | 0.323443 |
| 10 | 0.308176 | 0.943396 | 0.731656 | 0.341307 |
| 11 | 0.308176 | 0.959119 | 0.742138 | 0.3747 |
| 12 | 0.27673 | 0.927673 | 0.710692 | 0.277312 |
| 13 | 0.352201 | 0.924528 | 0.733753 | 0.349162 |
| 14 | 0.333333 | 0.940252 | 0.737945 | 0.360255 |
| 15 | 0.345912 | 0.908805 | 0.721174 | 0.315235 |
| 16 | 0.339623 | 0.924528 | 0.72956 | 0.33669 |
| 17 | 0.358491 | 0.921384 | 0.733753 | 0.349679 |
| 18 | 0.339623 | 0.927673 | 0.731656 | 0.342475 |
| 19 | 0.358491 | 0.921384 | 0.733753 | 0.349679 |
| 20 | 0.352201 | 0.915094 | 0.727463 | 0.332366 |
| 21 | 0.371069 | 0.933962 | 0.746331 | 0.384872 |
| 22 | 0.389937 | 0.902516 | 0.731656 | 0.347985 |
| 23 | 0.515723 | 0.874214 | 0.754717 | 0.421321 |
| 24 | 0.477987 | 0.877358 | 0.744235 | 0.391616 |
| 25 | 0.45283 | 0.874214 | 0.733753 | 0.363716 |
| 26 | 0.509434 | 0.86478 | 0.746331 | 0.402192 |
| 27 | 0.471698 | 0.899371 | 0.756813 | 0.419348 |
| 28 | 0.515723 | 0.867925 | 0.750524 | 0.412332 |
| 29 | 0.522013 | 0.871069 | 0.754717 | 0.422471 |
| 30 | 0.522013 | 0.886792 | 0.765199 | 0.445362 |
| 31 | 0.509434 | 0.858491 | 0.742138 | 0.393398 |
| 32 | 0.515723 | 0.855346 | 0.742138 | 0.394775 |
| 33 | 0.490566 | 0.880503 | 0.750524 | 0.407741 |
| 34 | 0.509434 | 0.842767 | 0.731656 | 0.371988 |
| 35 | 0.528302 | 0.877358 | 0.761006 | 0.43714 |
| 36 | 0.490566 | 0.871069 | 0.744235 | 0.393974 |
| 37 | 0.490566 | 0.874214 | 0.746331 | 0.398524 |
| 38 | 0.471698 | 0.852201 | 0.725367 | 0.349968 |
| 39 | 0.509434 | 0.874214 | 0.752621 | 0.415649 |
| 40 | 0.503145 | 0.874214 | 0.750524 | 0.40996 |
| 41 | 0.540881 | 0.867925 | 0.75891 | 0.434921 |
| 42 | 0.540881 | 0.877358 | 0.765199 | 0.448343 |
| 43 | 0.528302 | 0.867925 | 0.754717 | 0.423659 |
| 44 | 0.553459 | 0.861635 | 0.75891 | 0.437384 |
| 45 | 0.54717 | 0.86478 | 0.75891 | 0.436134 |
| 46 | 0.540881 | 0.861635 | 0.754717 | 0.426146 |
| 47 | 0.559748 | 0.871069 | 0.767296 | 0.45611 |
| 48 | 0.54717 | 0.855346 | 0.752621 | 0.423146 |
| 49 | 0.540881 | 0.871069 | 0.761006 | 0.43936 |
| 50 | 0.528302 | 0.867925 | 0.754717 | 0.423659 |
| 51 | 0.540881 | 0.839623 | 0.740042 | 0.396434 |
| 52 | 0.54717 | 0.852201 | 0.750524 | 0.41888 |
| 53 | 0.534591 | 0.852201 | 0.746331 | 0.407562 |
| 54 | 0.559748 | 0.889937 | 0.779874 | 0.483307 |
| 55 | 0.522013 | 0.86478 | 0.750524 | 0.413572 |
| 56 | 0.54717 | 0.86478 | 0.75891 | 0.436134 |
| 57 | 0.54717 | 0.871069 | 0.763103 | 0.444958 |
| 58 | 0.515723 | 0.871069 | 0.752621 | 0.416808 |
| 59 | 0.534591 | 0.861635 | 0.752621 | 0.420505 |
| 60 | 0.528302 | 0.845912 | 0.740042 | 0.393393 |
| 61 | 0.528302 | 0.867925 | 0.754717 | 0.423659 |
| 62 | 0.534591 | 0.867925 | 0.756813 | 0.429298 |
| 63 | 0.54717 | 0.86478 | 0.75891 | 0.436134 |
| 64 | 0.559748 | 0.871069 | 0.767296 | 0.45611 |
| 65 | 0.540881 | 0.852201 | 0.748428 | 0.413228 |
| 66 | 0.509434 | 0.852201 | 0.737945 | 0.384738 |
| 67 | 0.559748 | 0.861635 | 0.761006 | 0.442981 |
| 68 | 0.553459 | 0.845912 | 0.748428 | 0.416092 |
| 69 | 0.515723 | 0.867925 | 0.750524 | 0.412332 |
| 70 | 0.540881 | 0.849057 | 0.746331 | 0.408985 |
| 71 | 0.553459 | 0.861635 | 0.75891 | 0.437384 |
| 72 | 0.528302 | 0.86478 | 0.752621 | 0.419237 |
| 73 | 0.522013 | 0.845912 | 0.737945 | 0.387681 |
| 74 | 0.540881 | 0.842767 | 0.742138 | 0.400588 |
| 75 | 0.54717 | 0.842767 | 0.744235 | 0.406263 |
| 76 | 0.540881 | 0.871069 | 0.761006 | 0.43936 |
| 77 | 0.522013 | 0.861635 | 0.748428 | 0.409175 |
| 78 | 0.540881 | 0.855346 | 0.750524 | 0.417502 |
| 79 | 0.572327 | 0.852201 | 0.75891 | 0.441346 |
| 80 | 0.534591 | 0.849057 | 0.744235 | 0.403311 |
| 81 | 0.522013 | 0.849057 | 0.740042 | 0.391916 |
| 82 | 0.54717 | 0.852201 | 0.750524 | 0.41888 |
| 83 | 0.553459 | 0.855346 | 0.754717 | 0.428774 |
| 84 | 0.522013 | 0.858491 | 0.746331 | 0.404811 |
| 85 | 0.509434 | 0.871069 | 0.750524 | 0.411127 |
| 86 | 0.54717 | 0.842767 | 0.744235 | 0.406263 |
| 87 | 0.54717 | 0.827044 | 0.733753 | 0.385803 |
| 88 | 0.496855 | 0.855346 | 0.735849 | 0.377552 |
| 89 | 0.515723 | 0.871069 | 0.752621 | 0.416808 |
| 90 | 0.503145 | 0.855346 | 0.737945 | 0.383311 |
| 91 | 0.553459 | 0.86478 | 0.761006 | 0.441736 |
| 92 | 0.578616 | 0.852201 | 0.761006 | 0.44693 |
| 93 | 0.54717 | 0.855346 | 0.752621 | 0.423146 |
| 94 | 0.477987 | 0.855346 | 0.72956 | 0.360161 |
| 95 | 0.534591 | 0.861635 | 0.752621 | 0.420505 |
| 96 | 0.534591 | 0.839623 | 0.737945 | 0.390738 |
| 97 | 0.566038 | 0.852201 | 0.756813 | 0.43575 |
| 98 | 0.503145 | 0.849057 | 0.733753 | 0.374701 |
| 99 | 0.534591 | 0.845912 | 0.742138 | 0.39909 |
| 100 | 0.566038 | 0.871069 | 0.769392 | 0.461664 |
| 101 | 0.534591 | 0.861635 | 0.752621 | 0.420505 |
| 102 | 0.484277 | 0.867925 | 0.740042 | 0.383697 |
| 103 | 0.528302 | 0.871069 | 0.756813 | 0.428117 |
| 104 | 0.572327 | 0.877358 | 0.775681 | 0.476085 |
| 105 | 0.534591 | 0.852201 | 0.746331 | 0.407562 |
| 106 | 0.559748 | 0.845912 | 0.750524 | 0.421731 |
| 107 | 0.522013 | 0.839623 | 0.733753 | 0.3793 |
| 108 | 0.534591 | 0.86478 | 0.754717 | 0.424885 |
| 109 | 0.528302 | 0.849057 | 0.742138 | 0.397621 |
| 110 | 0.528302 | 0.867925 | 0.754717 | 0.423659 |
| 111 | 0.534591 | 0.858491 | 0.750524 | 0.416158 |
| 112 | 0.528302 | 0.855346 | 0.746331 | 0.406171 |
| 113 | 0.540881 | 0.849057 | 0.746331 | 0.408985 |
| 114 | 0.522013 | 0.827044 | 0.725367 | 0.362885 |
| 115 | 0.553459 | 0.842767 | 0.746331 | 0.411923 |
| 116 | 0.509434 | 0.855346 | 0.740042 | 0.389052 |
| 117 | 0.54717 | 0.855346 | 0.752621 | 0.423146 |
| 118 | 0.528302 | 0.845912 | 0.740042 | 0.393393 |
| 119 | 0.553459 | 0.845912 | 0.748428 | 0.416092 |
| 120 | 0.528302 | 0.877358 | 0.761006 | 0.43714 |
| 121 | 0.522013 | 0.880503 | 0.761006 | 0.436091 |
| 122 | 0.540881 | 0.855346 | 0.750524 | 0.417502 |
| 123 | 0.534591 | 0.842767 | 0.740042 | 0.394899 |
| 124 | 0.528302 | 0.852201 | 0.744235 | 0.40188 |
| 125 | 0.553459 | 0.845912 | 0.748428 | 0.416092 |
| 126 | 0.528302 | 0.852201 | 0.744235 | 0.40188 |
| 127 | 0.528302 | 0.861635 | 0.750524 | 0.414848 |
| 128 | 0.528302 | 0.874214 | 0.75891 | 0.43261 |
| 129 | 0.54717 | 0.849057 | 0.748428 | 0.414644 |
| 130 | 0.54717 | 0.858491 | 0.754717 | 0.427443 |
| 131 | 0.54717 | 0.852201 | 0.750524 | 0.41888 |
| 132 | 0.572327 | 0.852201 | 0.75891 | 0.441346 |
| 133 | 0.54717 | 0.861635 | 0.756813 | 0.431772 |
| 134 | 0.522013 | 0.86478 | 0.750524 | 0.413572 |
| 135 | 0.534591 | 0.852201 | 0.746331 | 0.407562 |
| 136 | 0.566038 | 0.852201 | 0.756813 | 0.43575 |
| 137 | 0.534591 | 0.830189 | 0.731656 | 0.378423 |
| 138 | 0.54717 | 0.86478 | 0.75891 | 0.436134 |
| 139 | 0.540881 | 0.852201 | 0.748428 | 0.413228 |
| 140 | 0.54717 | 0.839623 | 0.742138 | 0.402115 |
| 141 | 0.540881 | 0.858491 | 0.752621 | 0.421808 |
| 142 | 0.559748 | 0.867925 | 0.765199 | 0.4517 |
| 143 | 0.509434 | 0.839623 | 0.72956 | 0.3678 |
| 144 | 0.540881 | 0.830189 | 0.733753 | 0.384139 |
| 145 | 0.540881 | 0.830189 | 0.733753 | 0.384139 |
| 146 | 0.54717 | 0.845912 | 0.746331 | 0.410439 |
| 147 | 0.515723 | 0.855346 | 0.742138 | 0.394775 |
| 148 | 0.540881 | 0.858491 | 0.752621 | 0.421808 |
| 149 | 0.540881 | 0.855346 | 0.750524 | 0.417502 |
| 150 | 0.559748 | 0.845912 | 0.750524 | 0.421731 |
| 151 | 0.528302 | 0.852201 | 0.744235 | 0.40188 |
| 152 | 0.528302 | 0.849057 | 0.742138 | 0.397621 |
| 153 | 0.54717 | 0.849057 | 0.748428 | 0.414644 |
| 154 | 0.534591 | 0.86478 | 0.754717 | 0.424885 |
| 155 | 0.553459 | 0.823899 | 0.733753 | 0.387492 |
| 156 | 0.553459 | 0.858491 | 0.756813 | 0.433063 |
| 157 | 0.534591 | 0.855346 | 0.748428 | 0.411844 |
| 158 | 0.528302 | 0.861635 | 0.750524 | 0.414848 |
| 159 | 0.528302 | 0.858491 | 0.748428 | 0.410493 |
| 160 | 0.503145 | 0.861635 | 0.742138 | 0.392053 |
| 161 | 0.553459 | 0.845912 | 0.748428 | 0.416092 |
| 162 | 0.54717 | 0.845912 | 0.746331 | 0.410439 |
| 163 | 0.515723 | 0.849057 | 0.737945 | 0.386195 |
| 164 | 0.54717 | 0.858491 | 0.754717 | 0.427443 |
| 165 | 0.54717 | 0.86478 | 0.75891 | 0.436134 |
| 166 | 0.559748 | 0.874214 | 0.769392 | 0.460553 |
| 167 | 0.54717 | 0.855346 | 0.752621 | 0.423146 |
| 168 | 0.515723 | 0.833333 | 0.727463 | 0.365282 |
| 169 | 0.534591 | 0.842767 | 0.740042 | 0.394899 |
| 170 | 0.566038 | 0.836478 | 0.746331 | 0.414981 |
| 171 | 0.528302 | 0.852201 | 0.744235 | 0.40188 |
| 172 | 0.540881 | 0.86478 | 0.756813 | 0.430517 |
| 173 | 0.553459 | 0.849057 | 0.750524 | 0.420289 |
| 174 | 0.553459 | 0.877358 | 0.769392 | 0.459483 |
| 175 | 0.566038 | 0.86478 | 0.765199 | 0.452898 |
| 176 | 0.522013 | 0.858491 | 0.746331 | 0.404811 |
| 177 | 0.528302 | 0.852201 | 0.744235 | 0.40188 |
| 178 | 0.559748 | 0.845912 | 0.750524 | 0.421731 |
| 179 | 0.540881 | 0.855346 | 0.750524 | 0.417502 |
| 180 | 0.540881 | 0.852201 | 0.748428 | 0.413228 |
| 181 | 0.553459 | 0.86478 | 0.761006 | 0.441736 |
| 182 | 0.522013 | 0.855346 | 0.744235 | 0.400481 |
| 183 | 0.540881 | 0.852201 | 0.748428 | 0.413228 |
| 184 | 0.528302 | 0.849057 | 0.742138 | 0.397621 |
| 185 | 0.540881 | 0.874214 | 0.763103 | 0.443834 |
| 186 | 0.578616 | 0.839623 | 0.752621 | 0.430324 |
| 187 | 0.534591 | 0.858491 | 0.750524 | 0.416158 |
| 188 | 0.54717 | 0.867925 | 0.761006 | 0.440529 |
| 189 | 0.553459 | 0.852201 | 0.752621 | 0.424517 |
| 190 | 0.528302 | 0.867925 | 0.754717 | 0.423659 |
| 191 | 0.553459 | 0.849057 | 0.750524 | 0.420289 |
| 192 | 0.553459 | 0.833333 | 0.740042 | 0.399587 |
| 193 | 0.54717 | 0.86478 | 0.75891 | 0.436134 |
| 194 | 0.534591 | 0.858491 | 0.750524 | 0.416158 |
| 195 | 0.540881 | 0.861635 | 0.754717 | 0.426146 |
| 196 | 0.528302 | 0.861635 | 0.750524 | 0.414848 |
| 197 | 0.515723 | 0.86478 | 0.748428 | 0.407891 |
| 198 | 0.522013 | 0.852201 | 0.742138 | 0.396183 |
| 199 | 0.534591 | 0.839623 | 0.737945 | 0.390738 |
| 200 | 0.522013 | 0.855346 | 0.744235 | 0.400481 |
| 201 | 0.54717 | 0.858491 | 0.754717 | 0.427443 |
| 202 | 0.553459 | 0.823899 | 0.733753 | 0.387492 |
| 203 | 0.578616 | 0.842767 | 0.754717 | 0.434433 |
| 204 | 0.566038 | 0.861635 | 0.763103 | 0.448564 |
| 205 | 0.553459 | 0.842767 | 0.746331 | 0.411923 |
| 206 | 0.559748 | 0.861635 | 0.761006 | 0.442981 |
| 207 | 0.522013 | 0.852201 | 0.742138 | 0.396183 |
| 208 | 0.534591 | 0.842767 | 0.740042 | 0.394899 |
| 209 | 0.515723 | 0.842767 | 0.733753 | 0.37774 |
| 210 | 0.553459 | 0.86478 | 0.761006 | 0.441736 |
| 211 | 0.515723 | 0.839623 | 0.731656 | 0.373558 |
| 212 | 0.540881 | 0.849057 | 0.746331 | 0.408985 |
| 213 | 0.522013 | 0.849057 | 0.740042 | 0.391916 |
| 214 | 0.540881 | 0.855346 | 0.750524 | 0.417502 |
| 215 | 0.591195 | 0.836478 | 0.754717 | 0.437455 |
| 216 | 0.553459 | 0.855346 | 0.754717 | 0.428774 |
| 217 | 0.534591 | 0.849057 | 0.744235 | 0.403311 |
| 218 | 0.566038 | 0.836478 | 0.746331 | 0.414981 |
| 219 | 0.522013 | 0.852201 | 0.742138 | 0.396183 |
| 220 | 0.553459 | 0.852201 | 0.752621 | 0.424517 |
| 221 | 0.515723 | 0.852201 | 0.740042 | 0.390469 |
| 222 | 0.515723 | 0.839623 | 0.731656 | 0.373558 |
| 223 | 0.54717 | 0.855346 | 0.752621 | 0.423146 |
| 224 | 0.534591 | 0.852201 | 0.746331 | 0.407562 |
| 225 | 0.509434 | 0.855346 | 0.740042 | 0.389052 |
| 226 | 0.534591 | 0.858491 | 0.750524 | 0.416158 |
| 227 | 0.553459 | 0.833333 | 0.740042 | 0.399587 |
| 228 | 0.540881 | 0.836478 | 0.737945 | 0.392308 |
| 229 | 0.566038 | 0.845912 | 0.752621 | 0.427357 |
| 230 | 0.54717 | 0.867925 | 0.761006 | 0.440529 |
| 231 | 0.553459 | 0.830189 | 0.737945 | 0.395529 |
| 232 | 0.540881 | 0.845912 | 0.744235 | 0.404772 |
| 233 | 0.553459 | 0.849057 | 0.750524 | 0.420289 |
| 234 | 0.522013 | 0.86478 | 0.750524 | 0.413572 |
| 235 | 0.528302 | 0.845912 | 0.740042 | 0.393393 |
| 236 | 0.540881 | 0.842767 | 0.742138 | 0.400588 |
| 237 | 0.534591 | 0.849057 | 0.744235 | 0.403311 |
| 238 | 0.534591 | 0.867925 | 0.756813 | 0.429298 |
| 239 | 0.528302 | 0.839623 | 0.735849 | 0.385027 |
| 240 | 0.553459 | 0.839623 | 0.744235 | 0.407783 |
| 241 | 0.528302 | 0.81761 | 0.721174 | 0.356624 |
| 242 | 0.54717 | 0.849057 | 0.748428 | 0.414644 |
| 243 | 0.553459 | 0.830189 | 0.737945 | 0.395529 |
| 244 | 0.54717 | 0.855346 | 0.752621 | 0.423146 |
| 245 | 0.515723 | 0.867925 | 0.750524 | 0.412332 |
| 246 | 0.566038 | 0.852201 | 0.756813 | 0.43575 |
| 247 | 0.540881 | 0.830189 | 0.733753 | 0.384139 |
| 248 | 0.553459 | 0.858491 | 0.756813 | 0.433063 |
| 249 | 0.528302 | 0.852201 | 0.744235 | 0.40188 |
| 250 | 0.528302 | 0.867925 | 0.754717 | 0.423659 |
| 251 | 0.572327 | 0.833333 | 0.746331 | 0.416554 |
| 252 | 0.54717 | 0.839623 | 0.742138 | 0.402115 |
| 253 | 0.540881 | 0.839623 | 0.740042 | 0.396434 |
| 254 | 0.540881 | 0.823899 | 0.72956 | 0.376077 |
| 255 | 0.534591 | 0.861635 | 0.752621 | 0.420505 |
| 256 | 0.528302 | 0.839623 | 0.735849 | 0.385027 |
| 257 | 0.509434 | 0.833333 | 0.725367 | 0.359511 |
| 258 | 0.534591 | 0.871069 | 0.75891 | 0.433746 |
| 259 | 0.515723 | 0.858491 | 0.744235 | 0.399113 |
| 260 | 0.559748 | 0.852201 | 0.754717 | 0.43014 |
| 261 | 0.566038 | 0.836478 | 0.746331 | 0.414981 |
| 262 | 0.584906 | 0.842767 | 0.756813 | 0.44003 |
| 263 | 0.559748 | 0.842767 | 0.748428 | 0.41757 |
| 264 | 0.540881 | 0.814465 | 0.72327 | 0.364175 |
| 265 | 0.553459 | 0.839623 | 0.744235 | 0.407783 |
| 266 | 0.528302 | 0.849057 | 0.742138 | 0.397621 |
| 267 | 0.553459 | 0.823899 | 0.733753 | 0.387492 |
| 268 | 0.54717 | 0.858491 | 0.754717 | 0.427443 |
| 269 | 0.572327 | 0.836478 | 0.748428 | 0.420617 |
| 270 | 0.597484 | 0.86478 | 0.775681 | 0.480577 |
| 271 | 0.528302 | 0.867925 | 0.754717 | 0.423659 |
| 272 | 0.534591 | 0.852201 | 0.746331 | 0.407562 |
| 273 | 0.559748 | 0.858491 | 0.75891 | 0.438669 |
| 274 | 0.534591 | 0.845912 | 0.742138 | 0.39909 |
| 275 | 0.528302 | 0.867925 | 0.754717 | 0.423659 |
| 276 | 0.540881 | 0.858491 | 0.752621 | 0.421808 |
| 277 | 0.566038 | 0.833333 | 0.744235 | 0.410911 |
| 278 | 0.559748 | 0.86478 | 0.763103 | 0.447324 |
| 279 | 0.572327 | 0.830189 | 0.744235 | 0.412517 |
| 280 | 0.54717 | 0.839623 | 0.742138 | 0.402115 |
| 281 | 0.553459 | 0.845912 | 0.748428 | 0.416092 |
| 282 | 0.54717 | 0.858491 | 0.754717 | 0.427443 |
| 283 | 0.509434 | 0.861635 | 0.744235 | 0.397778 |
| 284 | 0.566038 | 0.833333 | 0.744235 | 0.410911 |
| 285 | 0.54717 | 0.852201 | 0.750524 | 0.41888 |
| 286 | 0.540881 | 0.833333 | 0.735849 | 0.38821 |
| 287 | 0.54717 | 0.830189 | 0.735849 | 0.389841 |
| 288 | 0.534591 | 0.849057 | 0.744235 | 0.403311 |
| 289 | 0.553459 | 0.842767 | 0.746331 | 0.411923 |
| 290 | 0.559748 | 0.842767 | 0.748428 | 0.41757 |
| 291 | 0.54717 | 0.842767 | 0.744235 | 0.406263 |
| 292 | 0.553459 | 0.852201 | 0.752621 | 0.424517 |
| 293 | 0.553459 | 0.836478 | 0.742138 | 0.403671 |
| 294 | 0.528302 | 0.836478 | 0.733753 | 0.380887 |
| 295 | 0.566038 | 0.855346 | 0.75891 | 0.43999 |
| 296 | 0.54717 | 0.842767 | 0.744235 | 0.406263 |
| 297 | 0.534591 | 0.855346 | 0.748428 | 0.411844 |
| 298 | 0.584906 | 0.830189 | 0.748428 | 0.423783 |
| 299 | 0.553459 | 0.86478 | 0.761006 | 0.441736 |
| 300 | 0.553459 | 0.839623 | 0.744235 | 0.407783 |
| 301 | 0.522013 | 0.849057 | 0.740042 | 0.391916 |
| 302 | 0.528302 | 0.845912 | 0.740042 | 0.393393 |
| 303 | 0.528302 | 0.855346 | 0.746331 | 0.406171 |
| 304 | 0.54717 | 0.852201 | 0.750524 | 0.41888 |
| 305 | 0.553459 | 0.833333 | 0.740042 | 0.399587 |
| 306 | 0.54717 | 0.867925 | 0.761006 | 0.440529 |
| 307 | 0.54717 | 0.86478 | 0.75891 | 0.436134 |
| 308 | 0.534591 | 0.849057 | 0.744235 | 0.403311 |
| 309 | 0.566038 | 0.836478 | 0.746331 | 0.414981 |
| 310 | 0.553459 | 0.830189 | 0.737945 | 0.395529 |
| 311 | 0.54717 | 0.861635 | 0.756813 | 0.431772 |
| 312 | 0.528302 | 0.867925 | 0.754717 | 0.423659 |
| 313 | 0.540881 | 0.833333 | 0.735849 | 0.38821 |
| 314 | 0.553459 | 0.839623 | 0.744235 | 0.407783 |
| 315 | 0.54717 | 0.845912 | 0.746331 | 0.410439 |
| 316 | 0.559748 | 0.855346 | 0.756813 | 0.434389 |
| 317 | 0.553459 | 0.830189 | 0.737945 | 0.395529 |
| 318 | 0.528302 | 0.845912 | 0.740042 | 0.393393 |
| 319 | 0.559748 | 0.845912 | 0.750524 | 0.421731 |
| 320 | 0.540881 | 0.849057 | 0.746331 | 0.408985 |
| 321 | 0.540881 | 0.852201 | 0.748428 | 0.413228 |
