# Supplementary material for: Analysis and Identification of Aptamer-Compound Interactions with a Maximum Relevance Minimum Redundancy and Nearest Neighbor Algorithm
Source: Biomed Res Int. 2016 Feb 3;2016:8351204. doi: 10.1155/2016/8351204 (PMC4756144; doi:10.1155/2016/8351204)
Supplement: Supplementary file 1 — The Supplementary Material contains four files. In detail, the Supplementary Material I lists 159 positive interactions and 318 negative interactions; the Supplementary Material II lists MaxRel features list and mRMR features list; Supplementary Material III lists the SNs, SPs, ACCs and MCCs obtained by IFS and four basic prediction engines; Supplementary Material IV lists predicted results of all interactions obtained by the optimal prediction model. [file 8351204.f1.zip › Supp-IV.docx]

**Supplemental Material IV.** Predicted results of all interactions obtained by the optimal prediction model

| **Compound name** | **Aptamer name** | **Sequence of aptamer** | **Predicted class** | **True class** |
| --- | --- | --- | --- | --- |
| Arsenate | 20000526-arsenic-5 | CAGTCAGAATCCGGGCTTACACCCATTTGTTTATTGTGCA | Positive | Positive |
| Arsenate | 20000526-arsenic-8 | TGGGGATTGTACGTACACCACCAATTCACACGCGAGTATG | Positive | Positive |
| Arsenate | 20000526-arsenic-3 | TTACAGAACAACCAACGTCGCTCCGGGTACTTCTTCATCG | Positive | Positive |
| Arsenate | 20000526-arsenic-Ma-1 | TTACAGAACAACCAACGTCGCTCCGGGTACTTCTTCATCG | Positive | Positive |
| Arsenate | 20000526-arsenic-7 | ATGCAAACCCTTAAGAAAGTGGTCGTCCAAAAAACCATTG | Positive | Positive |
| Isoleucine | 14561881A-isoleucine-3 | RCGACCGUAUGMGAAAGUUGGG | Positive | Positive |
| Isoleucine | 15772067-isoleucine-1 | GGAUUGUGUUCCGGUAAUGAAACGAGUAAAAGAGCAAGUCC | Positive | Positive |
| Adenine | 11705996-adenine-Ma-1 | GGGACGAUUAUCGAAAAUCACCAGAUUGGACCCUGGUUAACGAUCCC | Positive | Positive |
| Adenine | 11705996-adenine-1 | ATAGGACGATTATCGAAAATCACCAGATTGGACCCTGGTTAACGAT | Positive | Positive |
| Dopamine | 9245404-Dopamine-4 | GGGAAUUCCGCGUGUGCCAGCCACGUAGAAUCGUCUCUGCGCCGGGGAGAGUGUCAUGGUUAACGGGUAACAGCUCGUGGAGACGACCUCGGUUUCGGUCCGUUCGGGAUCCUC | Positive | Positive |
| Dopamine | 9245404-Dopamine-5 | GGGAAUUCCGCGUGUGCACAGCCAGGGUAAGAGUCUAGAGUAGUUCAUUCCUAGUAUCUGGAGGCUGGUACUAUAAGGGUAGUACUGCCCGCGUACUGUCCGUUCGGGAUCCUC | Positive | Positive |
| Dopamine | 9245404-Dopamine-7 | GGGAAUUCCGCGUGUGCAUGGUUAAUGGAAUCCUGGGCUCCGGAGAUCACCCAUCGGAGGUAAUUUCUUUAGAAUGCUAGACGGACACUACCAGCGUGUCCGUUCGGGAUCCUC | Positive | Positive |
| Dopamine | 9245404-Dopamine-9 | GGGAAUUCCGCGUGUGCUUAACCUAAUGGAAUACAAUUCUAACAGGAAUAGGAGGGUAGCUCCAAGUACUAACGACCGUUCCGGUAGCCGGCUGGCUGUCCGUUCGGGAUCCUC | Positive | Positive |
| Dopamine | 9245404-dopamine-1 | GGGAAUUCCGCGUGUGCGCCGCGGAAGACGUUGGAAGGAUAGAUACCUACAACGGGGAAUAUAGAGGCCAGCACAUAGUGAGGCCCUCCUCCCAAGGUCCGUUCGGGAUCCUC | Positive | Positive |
| Dopamine | 9245404-dopamine-2 | GGGAAUUCCGCGUGUGCUGGCGGGGAGAACUUACAUGGAUUAGAGAGUGGUGCUUCAUAAGAAGGACAACUGCGUUCCAGCCGCACCCCCCCCCCAGUCCGUUCGGGAUCCUC | Positive | Positive |
| Dopamine | 9245404-dopamine-Ma-1 | GGGAAUUCCGCGUGUGCGCCGCGGAAGACGUUGGAAGGAUAGAUACCUACAACGGGGAAUAUAGAGGCCAGCACAUAGUGAGGCCCUCCUCCC | Positive | Positive |
| Dopamine | 9245404-Dopamine-8 | GGGAAUUCCGCGUGUGCUAAGUACUUGGCGGGAUAACGUCCAAGAGACGAGAGUCACGCACCUUAAACAGCUGUACUACGUGCCCCCGUAGGUGUCCGUUCGGGAUCCUC | Positive | Positive |
| Dopamine | 9245404-Dopamine-6 | GGGAAUUCCGCGUGUGCCUUUCCAUGUAUUAGAGAGGAGAGCGCAAACACGAAGGCUCGUCGUUACAGCCGAAGAGCUCCAGUACCAUGGUUCAUGAGUCCGUUCGGGAUCCUC | Positive | Positive |
| Chitin | 10743940-chitin-1 | TAGGGAATTCGTCGACGGATCCCCCAGCAGCACTGGTAGTGAGGCAGTTCACCGGTGGGGCGGTGAGTTTGGCTGCTATTTATCTCCAGGTCGACGC-ATGCGCCG | Positive | Positive |
| Chitin | 10743940-chitin-5 | TAGGGAATTCGTCGACGGATCCCCGTAACCCTGCGGGGGGGGGAGAAGGCAATGGGGGACAACTCGCCGGTAGCCATCCATATCTCCAGGTCGACGC-ATGCGCCG | Positive | Positive |
| Chitin | 10743940-chitin-6 | TAGGGAATTCGTCGACGGATCCCCTAAGGGGGGACTCAGCATTTTGTGCGGGCGGCGCTAACACAATCAGATAGAGCGGGGTTCTCCAGGTCGACGC-ATGCGCCG | Positive | Positive |
| Chitin | 10743940-chitin-3 | TAGGGAATTCGTCGACGGATCCGGCAAGATGTGCCCAGAACAGTTGCTTGTATGGTGGGGAGCGTCCATATTGGCTTAAACCTCCAGGTCGACGCATGCGCCG | Positive | Positive |
| Chitin | 10743940-chitin-2 | TAGGGAATTCGTCGACGGATCCTGCGCATGTGAAAGGTTGCCTAACTGGACAGGGTTTAGGAGCGACTAGACACAGCTCCAGGTCGACGC-ATGCGCCG | Positive | Positive |
| Chitin | 10743940-chitin-7 | TAGGGAATTCGTCGACGGATCCCGTACAGTGGGCTATATTGCTGACGTAAGAGCTGCTTTCAATGCATTGGCATAGATGTCACTCCAGGTCGACGCATGCGCCG | Positive | Positive |
| Cellobiose | 9576904-cellobiose-3 | GTCAAGGTGGGTGGGTGGGGTTGGTTGTTGTTTTGA | Positive | Positive |
| Cellobiose | 9576904-cellobiose-2 | TAGCGGGTGTGGTGGGTGGGGGAGGCATGGTTTTTGGTAA | Positive | Positive |
| Tyrosine | 10786843-L Tyrosine-3 | AAGGGCAGUCCCCCCUUCGCUGGGGGGGUGGUUGUAGGGCUAAAACAAACCACGGGUGAUACGGGGGCCAUACCCUAGGAAGGCCCUGCUCCC | Positive | Positive |
| Omethoate | 22261866-pesticides-2 | AAGCTTTTTTGACTGACTGCAGCGATTCTTGATCGCCACGGTCTGGAAAAAGAG | Positive | Positive |
| Omethoate | 22261866-pesticides-1 | AAGCTTGCTTTATAGCCTGCAGCGATTCTTGATCGGAAAAGGCTGAGAGCTACGC | Positive | Positive |
| N-Acetylneuraminic acid | 23042406-Neu5Ac-1 | GCGGAAGCGUGCUGGGCCUAGUCCGUGACUAUAUCGAUUGCCUUUCAGACUUGCAUAACCCAGAGGUCGAUCCUAGGGGGAGA | Positive | Positive |
| N-Acetylneuraminic acid | 23042406-Neu5Ac-5 | GCGGAAGCGUGCUGGGCCUUGAAACGCUUUAUCGUUUAACGUUGCCUUCUUUGCUGUCAUAACCCAGAGGUCGAU | Positive | Positive |
| N-Acetylneuraminic acid | 23042406-Neu5Ac-7 | GCGGAAGCGUGCUGGGCCGUUAUAACCGGGUUACUAUCUGCCAAGUAGUGGAAUUCCCAUAACCCAGAGGUCGAU | Positive | Positive |
| N-Acetylneuraminic acid | 23042406-Neu5Ac-Ma-1 | GACGCUGGGCCUAGUCCGUGACUAUAUCGAUUGCCUUUUCAGACUUGCGUC | Positive | Positive |
| N-Acetylneuraminic acid | 23042406-Neu5Ac-6 | GCGGAAGCGUGCUGGGCCUGCAUCCCUGAGUCGCAAUCUGUUCCAGUUAAUGGAUUUCAUAACCCAGAGGUCGAU | Positive | Positive |
| N-Acetylneuraminic acid | 23042406-Neu5Ac-3 | GCGGAAGCGUGCUGGGCCCGGGAAAUGGACCUAUAUCGUACCUUACAACUUGGUUAGAACAUAACCCAGAGGUCGAU | Positive | Positive |
| N-Acetylneuraminic acid | 23042406-Neu5Ac-2 | GCGGAAGCGUGCUGGGCCACUAUAUCGAUGCCUUUCAGACUUGGCAUAACCCAGAGGUCGAU | Positive | Positive |
| N-Acetylneuraminic acid | 23042406-Neu5Ac-4 | GCGGAAGCGUGCUGGGCCGACAUGAAUGGCACUAGAUCGACGUUAGCGAUGUGAUGAUCAUAACCCAGAGGUCGAU | Positive | Positive |
| N-Glycolylneuraminic acid | 23042406-Neu5Ac-1 | GCGGAAGCGUGCUGGGCCUAGUCCGUGACUAUAUCGAUUGCCUUUCAGACUUGCAUAACCCAGAGGUCGAUCCUAGGGGGAGA | Positive | Positive |
| Tryptophan | 21076782-L-Tryptophan-1 | CAATGGCAGGCACGTTGGTTAGGTCAGGTTT-GGGTTTCGTGCTT | Positive | Positive |
| Tryptophan | 21076782-L-Tryptophan-Ma-1 | AGCACGTTGGTAGGTCGGTTTGGGTTTCGTGC | Positive | Positive |
| Codeine | 17038331-codeine-Ma-1 | GGGACAGGGCUAGCAGUAGGAUUGGGUGAGGGGAUGUGCUG | Positive | Positive |
| Codeine | 17038331-codeine-Ma-2 | GGGACAGGGCUAGCUUAGUGCUAUGUGAGAAAAGGGUGUGGGGG | Positive | Positive |
| Codeine | 17038331-codeine-16 | GGGACAGGGCTAGCCTATAGTGAGGGTATTAAGGGTTGTGGGGGAGGCAAAGCTTCCG | Positive | Positive |
| Codeine | 17038331-codeine-6 | GGGACAGGGCTAGCAGTAGGATTGGGTGAGGGGATGTGCTGTGAGGCAAAGCTTCCG | Positive | Positive |
| Codeine | 17038331-codeine-9 | GGGACAGGGCTAGCAGGGTAAGGGGATTGGGAGTAGTGCCGTGTGAGGCAAAGCTTCCG | Positive | Positive |
| Codeine | 17038331-codeine-19 | GGGACAGGGCTAGCAGCGGTAAGGGTGGGGAGAATGGTGCTGTGAGGCAAAGCTTCCG | Positive | Positive |
| Codeine | 17038331-codeine-14 | GGGACAGGGCTAGCTTAGTGCTATGTGAGAAAAGGGTGTGGGGGAGGCAAAGCTTCCG | Positive | Positive |
| Codeine | 17038331-codeine-18 | GGGACAGGGCTAGCATCGGTGTAGGGAAGGGATATGATGTGGTGAGGCAAAGCTTCCG | Positive | Positive |
| Codeine | 17038331-codeine-20 | GGGACAGGGCTAGCAAAGGTTAAGGGTAGGGGATACTGCTGGTGAGGCAAAGCTTCCG | Positive | Positive |
| Codeine | 17038331-codeine-8 | GGGACAGGGCTAGCACCAAAAATAGGGGTAAGGGCATGGGGGTGAGGCAAAGCTTCCG | Positive | Positive |
| Codeine | 17038331-codeine-5 | GGGACAGGGCTAGCACATGGAGGCTTATAGGGATTCGTGCTGGGAGGCAAAGCTTCCG | Positive | Positive |
| Codeine | 17038331-codeine-1 | GGGACAGGGCTAGCAAAAGGGTGGTTGAAGGGACAGCTGGTGTGAGGCAAAGCTTCCG | Positive | Positive |
| Codeine | 17038331-codeine-10 | GGGACAGGGCTAGCGGACAAGAAGTGGGTAAGGGAATCCGTGGGAGGCAAAGCTTCCG | Positive | Positive |
| Codeine | 17038331-codeine-4 | GGGACAGGGCTAGCAAGAATAGGATGTGGGTAAAGGTGCTGGTGAGGCAAAGCTTCCG | Positive | Positive |
| Codeine | 17038331-codeine-7 | GGGACAGGGCTAGCACATTGAGGGAAAGGGAATTGAGTGTGGTGAGGCAAAGCTTCCG | Positive | Positive |
| Codeine | 17038331-codeine-3 | GGGACAGGGCTAGCCACAAGTGTGAAGGGATGGGAGTAGTGGTGAGGCAAAGCTTCCG | Positive | Positive |
| Codeine | 17038331-codeine-15 | GGGACAGGGCTAGCGTAATAAGTAGGGAAAGGGTTCCCGCTGGGAGGCAAAGCTTCCG | Positive | Positive |
| Codeine | 17038331-codeine-2 | GGGACAGGGCTAGCACAAGAATTAGGGTCGGGAAATGGTGTGTGAGGCAAAGCTTCCG | Positive | Positive |
| Codeine | 17038331-codeine-13 | GGGACAGGGCTAGCTACAGAATAAGCGAATTAAGGGTTGGGGTGAGGCAAAGCTTCCG | Positive | Positive |
| Codeine | 17038331-codeine-12 | GGGACAGGGCTAGCTACTAATGTACGCACTAAGGGATTGGGGTGAGGCAAAGCTTCCG | Positive | Positive |
| Codeine | 17038331-codeine-21 | GGGACAGGGCTAGCAGCAAATTGGGAAAGGGATACGCTGTGTGGAGGCAAAGCTTCCG | Positive | Positive |
| Codeine | 17038331-codeine-17 | GGGACAGGGCTAGCGCAGAACAGAGGGTAGGGAATTTGCGTGTGAGGCAAAGCTTCCG | Positive | Positive |
| Codeine | 17038331-codeine-11 | GGGACAGGGCTAGCCAATAAATAAGGCGAAGTAAGGGATGGGGTGAGGCAAAGCTTCCG | Positive | Positive |
| Cholic acid | 10978501-cholic acid-Ma-1 | GTACCAGCTTATTGCTGCGGGCTGAAGCCCGGTAC | Positive | Positive |
| Cholic acid | 10978501-cholic acid-3 | GCTTATTCAATTCGCGGAAGACGAATTCCAAGCGCGCGCGGGTCACGCGACTTGGGAATGAGC | Positive | Positive |
| Cyclic adenosine monophosphate | 10913311-cAMP-3 | UAAAUCGACUUGUCGCGUUGCAGGCUGCACCCCGUCCGUGGACCCCC | Positive | Positive |
| Cyclic adenosine monophosphate | 10913311-cAMP-2 | UAAAACGACUUGUCGCGUGCUGCCCGCCUGUUCGCUUUCUGCACCCC | Positive | Positive |
| S-Adenosyl-L-homocysteine | 10852725-SAH-1 | GGGCGAAUUCAGGACUCCUGACAUGUAGUUGAUUGAAGGACGUUUGAUUCUUAUGGAUUGUGGAAAGCUUCGGUCAUCAGUGCGCAGGAUCCAUC | Positive | Positive |
| S-Adenosyl-L-homocysteine | 10852725-SAH-2 | GGGCGAAUUCAGGACUCCUGACAUAUGAUGUUUUAUGGUUAUGCUCUGCGAAAUCACUGAUGAGACGCUUGGCGUGUGCUGUGGAGAGUCAUCAGUGCGCAGGAUCCAUC | Positive | Positive |
| Adenosine triphosphate | 11101810-ATP-1 | GAAGCAAGCAGGCAACGAACACAGAAGACCGGGGGAACUACCGCGCGUGCC | Positive | Positive |
| Ochratoxin A | 18983163-ochratoxin A-21 | GATCGGGTGTGGGTGGCGTAAAGG | Positive | Positive |
| Ochratoxin A | 18983163-ochratoxin A-20 | GATCGGGTGTGGGTGGCGTAAAGGGAG | Positive | Positive |
| Ochratoxin A | 18983163-ochratoxin A-24 | TCGGGTGTGGGTGGCGTAAAGGGAGCATCGG | Positive | Positive |
| Ochratoxin A | 18983163-ochratoxin A-25 | ATCGGGTGTGGGTGGCGTAAAG | Positive | Positive |
| Ochratoxin A | 18983163-ochratoxin A--Ma-16 | GATCGGGTGTGGGTGGCGTAAAGGGAGCATCGG | Positive | Positive |
| Ochratoxin A | 18983163-ochratoxin A-22 | ATCGGGTGTGGGTGGCGTAAAGGGAGCATCGG | Positive | Positive |
| Ochratoxin A | 18983163-ochratoxin A-19 | GATCGGGTGTGGGTGGCGTAAAGGGAGCAT | Positive | Positive |
| Ochratoxin A | 18983163-ochratoxin A-Ma-17 | GATCGGGTGTGGGTGGCGTAAAGGGAGCATCG | Positive | Positive |
| Ochratoxin A | 18983163-ochratoxin A-Ma-18 | GATCGGGTGTGGGTGGCGTAAAGGGAGCATC | Positive | Positive |
| Ochratoxin A | 18983163-ochratoxin A-9 | TGGTGGCTGTAGGTCACAGGTGGCAGATCGGGTGTGGGTGGCCTGGGAGCATCGGACAACG | Positive | Positive |
| Ochratoxin A | 18983163-ochratoxin A-14 | TGGTGGCTGTAGGTCAGCAGTACGATCGGGGGTGGGTGGATGTAGGGAGCATCGGACAACG | Positive | Positive |
| Ochratoxin A | 18983163-ochratoxin A-Ma-15 | GATCGGGTGTGGGTGGCGTAAAGGGAGCATCGGACA | Positive | Positive |
| Ochratoxin A | 18983163-ochratoxin A-5 | TGGTGGCTGTAGGTCAACTGTCCGTCGGGTTTAGGGTGGCGTTCGGGAGCATCGGACAACG | Positive | Positive |
| Ochratoxin A | 18983163-ochratoxin A-11 | TGGTGGCTGTAGGTCACCTGACGATCGGGTGTGGGTTGGCTTGAGGGAGCATCGGACAACG | Positive | Positive |
| Ochratoxin A | 18983163-ochratoxin A-12 | TGGTGGCTGTAGGTCACCTTGTAGATCGGGTGTGGTTTGGCGTAGGGAGCATCGGACAACG | Positive | Positive |
| Ochratoxin A | 18983163-ochratoxin A-Ma-13 | GATTCGGGTGTGGGTGGCGTAAAGGGAGCATCGGACAACG | Positive | Positive |
| Ochratoxin A | 18983163-ochratoxin A-1 | TGGTGGCTGTAGGTCAGCATCTGATCGGGTGTGGGTGGCGTAAAGGGAGCATCGGACAACG | Positive | Positive |
| Ochratoxin A | 18983163-ochratoxin A-3 | TGGTGGCTGTAGGTCAGCAGTCCTAGATCGGGTGTGGCTGGCTTGGGAGCATCGGACAACG | Positive | Positive |
| Ochratoxin A | 18983163-ochratoxin A-2 | TGGTGGCTGTAGGTCAGGGGTGAAACGGGTCCCGGAGCATCGGACAACG | Positive | Positive |
| Ochratoxin A | 18983163-ochratoxin A-4 | TGGTGGCTGTAGGTCAGCACGATGGGGAAAGGGTCCCCCTGGGTTGGAGCATCGGACAACG | Positive | Positive |
| Ochratoxin A | 18983163-ochratoxin A-6 | TGGTGGCTGTAGGTCATCAGTCCCGATCAGGTGTGGGTGGCATTGGGAGCATCGGACAACG | Positive | Positive |
| Ochratoxin A | 18983163-ochratoxin A-8 | TGGTGGCTGTAGGTCACGTACGGTGGGAACGGTTCCTCTTAGGGTGAGCATCGGACAACG | Positive | Positive |
| Ochratoxin A | 18983163-ochratoxin A-23 | GATCGGGTGTCGGTGGCGTAAAGGGAGCATCGGACAACG | Positive | Positive |
| Ochratoxin A | 18983163-ochratoxin A-7 | TGGTGGCTGTAGGTCACCAAATGCGACGGGGCCTGTTTTAATGGGGGAGCATCGGACAACG | Positive | Positive |
| Ochratoxin A | 18983163-ochratoxin A-10 | TGGTGGCTGTAGGTCAACATGCGACTGAGGCTCGGTTTATTGAGGGGAGCATCGGACAACG | Positive | Positive |
| Sulforhodamine B | 9831529-sulforhodamine B-Ma-5 | CCGGCCAAGGGGTGGGGAGGGGAGGGGGGCCGG | Positive | Positive |
| Sulforhodamine B | 9831529-sulforhodamine B-Ma-16 | CCGGCCGGGTGGGAGGGAGGGGGCCGG | Positive | Positive |
| Sulforhodamine B | 9831529-sulforhodamine B-Ma-15 | CCGGCCAGGGTGGGAGGGAGGGGGCCGG | Positive | Positive |
| Sulforhodamine B | 9831529-sulforhodamine B-Ma-10 | CCGGCCTAGGGTGGGAGGGAGGGGGCCGG | Positive | Positive |
| Sulforhodamine B | 9831529-sulforhodamine B-Ma-11 | CCGGCCATGGGTGGGAGGGAGGGGGCCGG | Positive | Positive |
| Sulforhodamine B | 9831529-sulforhodamine B-Ma-14 | CCGGCCAAGGGTGGGAGGGTGGGGGCCGG | Positive | Positive |
| Sulforhodamine B | 9831529-sulforhodamine B-Ma-13 | CCGGCCAAGGGTGGGTGGGAGGGGGCCGG | Positive | Positive |
| Sulforhodamine B | 9831529-sulforhodamine B-Ma-4 | CCGGCCAAGGGTGGGAGGGAGGGGGCCGG | Positive | Positive |
| Sulforhodamine B | 9831529-sulforhodamine B-Ma-12 | CCGGCCAAGGGAGGGAGGGAGGGGGCCGG | Positive | Positive |
| Sulforhodamine B | 9831529-sulforhodamine B-Ma-6 | CCGGCCAAAGGTGGGAGGGAGGGGGCCGG | Positive | Positive |
| Sulforhodamine B | 9831529-sulforhodamine B-Ma-8 | CCGGCCAAGGGTGGGAGGGAAGGGGCCGG | Positive | Positive |
| Sulforhodamine B | 9831529-sulforhodamine B-Ma-20 | CCGGCCAAGGGTGGGAAGGAGGGGGCCGG | Positive | Positive |
| Sulforhodamine B | 9831529-sulforhodamine B-Ma-19 | CCGGCCAAGGGTGGAAGGGAGGGGGCCGG | Positive | Positive |
| Sulforhodamine B | 9831529-sulforhodamine B-Ma-17 | CCGGCCAAGGGTGGGAGGGAGGGAGGCCGG | Positive | Positive |
| Sulforhodamine B | 9831529-sulforhodamine B-Ma-3 | ATGACCAAGGGTGGGAGGGAGGGGGTCAT | Positive | Positive |
| Sulforhodamine B | 9831529-sulforhodamine B-Ma-9 | CCGGCAAAGGGTGGGAGGGAGGGCGCCGG | Positive | Positive |
| Sulforhodamine B | 9831529-sulforhodamine B-Ma-7 | CCGGCCAAGGGTGGGAGGGAGAGGGCCGG | Positive | Positive |
| Sulforhodamine B | 9831529-sulforhodamine B-Ma-21 | CCGGCCAAGGGTGGGAGGGAGGAGGCCGG | Positive | Positive |
| Sulforhodamine B | 9831529-sulforhodamine B-Ma-18 | CCGGCCAAGGTGGAGGAGGGGCCGG | Positive | Positive |
| Sulforhodamine B | 9889155-sulforhodamine B-Ma-1 | AUGGAGGGGCGCAAG | Positive | Positive |
| Sulforhodamine B | 9831529-sulforhodamine B-Ma-2 | CGGGATCCTAATGACCAAGGGTGGGAGGGAGGGGGTCATTAAATCCAG | Positive | Positive |
| Sulforhodamine B | 9831529-sulforhodamine B-1 | CGGGATCCTAATGACCAAGGGTGGGAGGGAGGGGGTCATTAAATCCAGTATCAACACGCCACGATGGGATCACCGCCATGGGCCGTCCCACTGGTGCCAGTCGGATAGTGTTCCTATAGTGAGTCGTATTAGAA | Positive | Positive |
| Hematoporphyrin IX | 11128644-Hematoporphyrin-4 | GGTGCGGGTAGGGATGAGGGCGGGTCGGGTTGAGTGTGAGCTTAGGGGTGGCGGATCTAC | Positive | Positive |
| Hematoporphyrin IX | 11128644-Hematoporphyrin-8 | CCAACTTGGGCGGAGGGCTAACGGTGGGGGGATATTATGAGGGGTGGAGGTATTAACCATT | Positive | Positive |
| Hematoporphyrin IX | 11128644-Hematoporphyrin-Ma-2 | ATGGTGGACGGAGATGGGACGTAG | Positive | Positive |
| Hematoporphyrin IX | 11128644-Hematoporphyrin-9 | CCGGGGATTAGAATAGTGGAGGGCCGGTGGCAAATGGGTAAGTAGGTTGAAGGGCTAAATT | Positive | Positive |
| Hematoporphyrin IX | 11128644-Hematoporphyrin-3 | CAGCGTAAGGTCGTGGGTGGTTGGGTGCCTAAGCGTATACTTACAGCGATTGATTTGGTC | Positive | Positive |
| Hematoporphyrin IX | 11128644-Hematoporphyrin-1 | TATGAACCAAGGGAGGGGCAGGGCGGGTAGGGTTAATAACGGATTCCGAGCGCCCAGCTC | Positive | Positive |
| Hematoporphyrin IX | 11128644-Hematoporphyrin-2 | ACGAAGAAACAGGGCGCTTCGAACGAACATGGGCAGGGTGGGAAGTTTTAAAGTGGTAC | Positive | Positive |
| Isoleucine | 14980623-sialyllactose-1 | AGCGGGGCCAGCCACTTCTGTCAGTGAATTCCTGCTCGTATATCTACTCGCCCGCCTGCG | Positive | Negative |
| Dopamine | 18983163-ochratoxin A-3 | TGGTGGCTGTAGGTCAGCAGTCCTAGATCGGGTGTGGCTGGCTTGGGAGCATCGGACAACG | Positive | Negative |
| Dopamine | 10743940-chitin-2 | TAGGGAATTCGTCGACGGATCCTGCGCATGTGAAAGGTTGCCTAACTGGACAGGGTTTAGGAGCGACTAGACACAGCTCCAGGTCGACGC-ATGCGCCG | Positive | Negative |
| Dopamine | 23042406-Neu5Ac-5 | GCGGAAGCGUGCUGGGCCUUGAAACGCUUUAUCGUUUAACGUUGCCUUCUUUGCUGUCAUAACCCAGAGGUCGAU | Positive | Negative |
| Dopamine | 10913311-cAMP-3 | UAAAUCGACUUGUCGCGUUGCAGGCUGCACCCCGUCCGUGGACCCCC | Positive | Negative |
| Chitin | 14561881B-isoleucine-3 | GCCANUACAUCUCGCUNUCGUACUNCG | Positive | Negative |
| Chitin | 10786843-L Tyrosine-3 | AAGGGCAGUCCCCCCUUCGCUGGGGGGGUGGUUGUAGGGCUAAAACAAACCACGGGUGAUACGGGGGCCAUACCCUAGGAAGGCCCUGCUCCC | Positive | Negative |
| Chitin | 9245404-Dopamine-4 | GGGAAUUCCGCGUGUGCCAGCCACGUAGAAUCGUCUCUGCGCCGGGGAGAGUGUCAUGGUUAACGGGUAACAGCUCGUGGAGACGACCUCGGUUUCGGUCCGUUCGGGAUCCUC | Positive | Negative |
| Chitin | 9245404-dopamine-Ma-1 | GGGAAUUCCGCGUGUGCGCCGCGGAAGACGUUGGAAGGAUAGAUACCUACAACGGGGAAUAUAGAGGCCAGCACAUAGUGAGGCCCUCCUCCC | Positive | Negative |
| Tyrosine | 23042406-Neu5Ac-4 | GCGGAAGCGUGCUGGGCCGACAUGAAUGGCACUAGAUCGACGUUAGCGAUGUGAUGAUCAUAACCCAGAGGUCGAU | Positive | Negative |
| Tyrosine | 10913311-cAMP-2 | UAAAACGACUUGUCGCGUGCUGCCCGCCUGUUCGCUUUCUGCACCCC | Positive | Negative |
| Tyrosine | 20000526-arsenic-Ma-1 | TTACAGAACAACCAACGTCGCTCCGGGTACTTCTTCATCG | Positive | Negative |
| N-Acetylneuraminic acid | 21076782-L-Tryptophan-1 | CAATGGCAGGCACGTTGGTTAGGTCAGGTTT-GGGTTTCGTGCTT | Positive | Negative |
| N-Acetylneuraminic acid | 9245404-Dopamine-5 | GGGAAUUCCGCGUGUGCACAGCCAGGGUAAGAGUCUAGAGUAGUUCAUUCCUAGUAUCUGGAGGCUGGUACUAUAAGGGUAGUACUGCCCGCGUACUGUCCGUUCGGGAUCCUC | Positive | Negative |
| N-Acetylneuraminic acid | 20000526-arsenic-1 | ACCATCGCGGAAGTCCAGTCTGCCATCAAAATCCGAAGTG | Positive | Negative |
| N-Glycolylneuraminic acid | 14561881B-isoleucine-4 | UACGCGUUGUCUUCCAGAGCCUGGCC | Positive | Negative |
| Bisphenol A | 14561881B-isoleucine-4 | UACGCGUUGUCUUCCAGAGCCUGGCC | Positive | Negative |
| Tryptophan | 18983163-ochratoxin A-5 | TGGTGGCTGTAGGTCAACTGTCCGTCGGGTTTAGGGTGGCGTTCGGGAGCATCGGACAACG | Positive | Negative |
| Acetamiprid | 10978501-cholic acid-5 | GCAGGGTCAATGGAATTAATGATCAATTGACAGACGCAAGTCTCCTGC | Positive | Negative |
| Phorate | 22261866-pesticides-1 | AAGCTTGCTTTATAGCCTGCAGCGATTCTTGATCGGAAAAGGCTGAGAGCTACGC | Positive | Negative |
| Cholic acid | 9576904-cellobiose-3 | GTCAAGGTGGGTGGGTGGGGTTGGTTGTTGTTTTGA | Positive | Negative |
| Cholic acid | 22261866-pesticides-1 | AAGCTTGCTTTATAGCCTGCAGCGATTCTTGATCGGAAAAGGCTGAGAGCTACGC | Positive | Negative |
| Cyclic adenosine monophosphate | 9245404-Dopamine-8 | GGGAAUUCCGCGUGUGCUAAGUACUUGGCGGGAUAACGUCCAAGAGACGAGAGUCACGCACCUUAAACAGCUGUACUACGUGCCCCCGUAGGUGUCCGUUCGGGAUCCUC | Positive | Negative |
| Cyclic adenosine monophosphate | 20000526-arsenic-3 | TTACAGAACAACCAACGTCGCTCCGGGTACTTCTTCATCG | Positive | Negative |
| Ochratoxin A | 21076782-L-Tryptophan-Ma-1 | AGCACGTTGGTAGGTCGGTTTGGGTTTCGTGC | Positive | Negative |
| Ochratoxin A | 11128644-Hematoporphyrin-2 | ACGAAGAAACAGGGCGCTTCGAACGAACATGGGCAGGGTGGGAAGTTTTAAAGTGGTAC | Positive | Negative |
| Ochratoxin A | 10743940-chitin-2 | TAGGGAATTCGTCGACGGATCCTGCGCATGTGAAAGGTTGCCTAACTGGACAGGGTTTAGGAGCGACTAGACACAGCTCCAGGTCGACGC-ATGCGCCG | Positive | Negative |
| Ochratoxin A | 22261866-pesticides-2 | AAGCTTTTTTGACTGACTGCAGCGATTCTTGATCGCCACGGTCTGGAAAAAGAG | Positive | Negative |
| Malachite green | 9245404-Dopamine-9 | GGGAAUUCCGCGUGUGCUUAACCUAAUGGAAUACAAUUCUAACAGGAAUAGGAGGGUAGCUCCAAGUACUAACGACCGUUCCGGUAGCCGGCUGGCUGUCCGUUCGGGAUCCUC | Positive | Negative |
| Malachite green | 9245404-dopamine-2 | GGGAAUUCCGCGUGUGCUGGCGGGGAGAACUUACAUGGAUUAGAGAGUGGUGCUUCAUAAGAAGGACAACUGCGUUCCAGCCGCACCCCCCCCCCAGUCCGUUCGGGAUCCUC | Positive | Negative |
| Thyroxine | 18983163-ochratoxin A-9 | TGGTGGCTGTAGGTCACAGGTGGCAGATCGGGTGTGGGTGGCCTGGGAGCATCGGACAACG | Positive | Negative |
| Sulforhodamine B | 10978501-cholic acid-6 | GTACCAGCTTATTCAATTACACGGACAGAGGGTAGCGGCTCTGCGCATTGAGTTGCTGCGGGCTGAAGCCCGGTAC | Positive | Negative |
| Sulforhodamine B | 23042406-Neu5Ac-Ma-1 | GACGCUGGGCCUAGUCCGUGACUAUAUCGAUUGCCUUUUCAGACUUGCGUC | Positive | Negative |
| Hematoporphyrin IX | 9831529-sulforhodamine B-Ma-17 | CCGGCCAAGGGTGGGAGGGAGGGAGGCCGG | Positive | Negative |
| Hematoporphyrin IX | 23042406-Neu5Ac-1 | GCGGAAGCGUGCUGGGCCUAGUCCGUGACUAUAUCGAUUGCCUUUCAGACUUGCAUAACCCAGAGGUCGAUCCUAGGGGGAGA | Positive | Negative |
| Arsenate | 20000526-arsenic-4 | TTCCGCTAGGGGAACATGATCAACATGGACCAAGTAAAC | Negative | Positive |
| Arsenate | 20000526-arsenic-1 | ACCATCGCGGAAGTCCAGTCTGCCATCAAAATCCGAAGTG | Negative | Positive |
| Arsenate | 20000526-arsenic-2 | CACGCGTTCAACCCCCGGAATTTAGCAATAGCAGATTACG | Negative | Positive |
| Arsenate | 20000526-arsenic-6 | CAATCTAAGCGAACCGCGTGCAGACCAATTACTCGCGCTA | Negative | Positive |
| Glutamic acid | 18187867-Glu-2 | CTGAGCCTGCGTGTATAGGACATTGACGGCGGTCGAAGCGGAGGCACGTAGCCAGGCAGAGGGACAGGTGGAGATAGCAGTATCGTCATGATCCATCG | Negative | Positive |
| Isoleucine | 14561881B-isoleucine-3 | GCCANUACAUCUCGCUNUCGUACUNCG | Negative | Positive |
| Isoleucine | 14561881B-isoleucine-4 | UACGCGUUGUCUUCCAGAGCCUGGCC | Negative | Positive |
| Chitin | 10743940-chitin-4 | TAGGGAATTCGTCGACGGATCCACCTGCGATAGGTAGTTTGTTTCTGTTGCCCTAGCGTCCGTCGTAACAAGTCACTCCAGGTCGACGCATGCGCCG | Negative | Positive |
| L-Arginine | 8604334-LArginine-1 | AUGAUAAACCGAUGCUGGGCGAUUCUCCUGAAGUAGGGGAAGAGUUGUCAUGUAUGGG | Negative | Positive |
| Cellobiose | 9576904-cellobiose-1 | GCGGGGTTGGGCGGGTGGGTTCGCTGGGCAGGGGGCGAGTG | Negative | Positive |
| Tyrosine | 10786843-L Tyrosine-2 | GAGCGACUGAGGUUCGCCGCGGAUUAUGUUUUGCGGUUAGAUCAGGCAACGGGUAAUACCGGGUCAGUCAGAUAGGGAGGAUCUACUGCC | Negative | Positive |
| Tyrosine | 10786843-L Tyrosine-1 | GGCAGUCAACUCGUGCGAUCGUGAAAACGGGGCAAGAUGGCCUUACAGCGGUCAAUACGGGGGUCAUCAGAUAGGGAGGCCCUCCUGGU | Negative | Positive |
| Bisphenol A | 21413891-Bisphenol A-1 | CCGGTGGGTGGTCAGGTGGGATAGCGTTCCGCGTATGGCCCAGCGCATCACGGGTTCGCACCA | Negative | Positive |
| Acetamiprid | 21306108-Acetamiprid-1 | TGTAATTTGTCTGCAGCGGTTCTTGATCGCTGACACCATATTATGAAGA | Negative | Positive |
| Phorate | 22261866-pesticides-2 | AAGCTTTTTTGACTGACTGCAGCGATTCTTGATCGCCACGGTCTGGAAAAAGAG | Negative | Positive |
| Phorate | 22261866-pesticides-1 | AAGCTTGCTTTATAGCCTGCAGCGATTCTTGATCGGAAAAGGCTGAGAGCTACGC | Negative | Positive |
| Cholic acid | 10978501-cholic acid-1 | GATCGAGGGCAGCGATAGCTGGGCTAATAAGGTTAGCCCCATCGGTC | Negative | Positive |
| Cholic acid | 10978501-cholic acid-6 | GTACCAGCTTATTCAATTACACGGACAGAGGGTAGCGGCTCTGCGCATTGAGTTGCTGCGGGCTGAAGCCCGGTAC | Negative | Positive |
| Cholic acid | 10978501-cholic acid-5 | GCAGGGTCAATGGAATTAATGATCAATTGACAGACGCAAGTCTCCTGC | Negative | Positive |
| Cholic acid | 10978501-cholic acid-4 | GAACATACGGCAGTTTATGGCCGCTATCGAGATAGACTATCATCTCAACGTCTTCTAGATAGTATGTTC | Negative | Positive |
| Cholic acid | 10978501-cholic acid-2 | AGCGCCGATTGACCCAAATCGTTTTGTATGCAAAAGCGCT | Negative | Positive |
| Cyclic adenosine monophosphate | 10913311-cAMP-4 | UAAAUCGACUUGUCGUAAACCCAUUCUUAUCGGCGUGCUCUACAGCCG | Negative | Positive |
| Cyclic adenosine monophosphate | 10913311-cAMP-1 | CCGACGCGUAAACGAAACGCACAUUCCCCCACAGGAUAUGUGCUCGG | Negative | Positive |
| Sialyllactose | 14980623-sialyllactose-1 | AGCGGGGCCAGCCACTTCTGTCAGTGAATTCCTGCTCGTATATCTACTCGCCCGCCTGCG | Negative | Positive |
| Adenosine triphosphate | 12873145-ATP-1 | GGCAGACGUAAGGCUGAGAUUGCCGUCCGUAAUUGAGUGCGGACGCGAGACGUCUGCC | Negative | Positive |
| Malachite green | 10339553-MalachiteGreen-1 | GGAACACUAUCCGACUGGCACCCCCCUGCCAGGUAACGAAUGAAGUGCUUUUCUCGAUCUCGUGACCCGCGCACUAGUCGCGAAGGUGUAUGUCCUUGGUCAUUAGGAUCCCG | Negative | Positive |
| Fumonisin B1 | 21614178-fumonisin B1-1 | ATACCAGCTTATTCAATTAATCGCATTACCTTATACCAGCTTATTCAATTACGTCTGCACATACCAGCTTATTCAATTAGATAGTAAGTGCAATCT | Negative | Positive |
| Thyroxine | 17163839-Thyroxine-Ma-1 | GGUGGAGGGGGACGUGCUGCAUCCGCAGUGCGUCUUGGGUUGUG | Negative | Positive |
| FADH | 11851395-FAD-1 | GGGCAUAAGGUAUUUAAUUCCAUACAAGUUUACAAGAAAGAUGC | Negative | Positive |
| Sulforhodamine B | 9889155-sulforhodamine B-1 | GAAGUUUUACCGUGUUGGUGGUCUAUGAGCCUACUGCCUGCAGUACAUGAGGUAUCGG | Negative | Positive |
| Sulforhodamine B | 9889155-sulforhodamine B-2 | GGACGGCACCACGGUCGGAUCCGUGAGUUGUGACAAUUUAGCGGGUGGUAUUAGAGCCUACUGCCACAGCAAUAGGAUCGAUACAGAUCU | Negative | Positive |
| Hematoporphyrin IX | 11128644-Hematoporphyrin-Ma-1 | ATGGGGTCGGGCGGGCCGGGTGTC | Negative | Positive |
| Hematoporphyrin IX | 11128644-Hematoporphyrin-6 | CAATGGGGTCGGGCGGGCCGGGTGTCATGGTGGACGGAGATGGGACGTAGAGGGCGGT | Negative | Positive |
| Hematoporphyrin IX | 11128644-Hematoporphyrin-5 | CTAGAGTAACCTGTTGGGAGGGCGGGTAGGGCCCATTGAGAGGAGGACGTATTCGTCCGC | Negative | Positive |
| Hematoporphyrin IX | 11128644-Hematoporphyrin-7 | GCGGCAAATCAGCATACGAGTGTACAGGGACGGGACGGGTGGGTAAAAGGTGTCGCCTCAG | Negative | Positive |
| Arsenate | 9831529-sulforhodamine B-Ma-5 | CCGGCCAAGGGGTGGGGAGGGGAGGGGGGCCGG | Negative | Negative |
| Arsenate | 9831529-sulforhodamine B-Ma-9 | CCGGCAAAGGGTGGGAGGGAGGGCGCCGG | Negative | Negative |
| Arsenate | 9889155-sulforhodamine B-Ma-1 | AUGGAGGGGCGCAAG | Negative | Negative |
| Arsenate | 11128644-Hematoporphyrin-9 | CCGGGGATTAGAATAGTGGAGGGCCGGTGGCAAATGGGTAAGTAGGTTGAAGGGCTAAATT | Negative | Negative |
| Arsenate | 18983163-ochratoxin A-5 | TGGTGGCTGTAGGTCAACTGTCCGTCGGGTTTAGGGTGGCGTTCGGGAGCATCGGACAACG | Negative | Negative |
| Arsenate | 18983163-ochratoxin A-8 | TGGTGGCTGTAGGTCACGTACGGTGGGAACGGTTCCTCTTAGGGTGAGCATCGGACAACG | Negative | Negative |
| Arsenate | 17038331-codeine-7 | GGGACAGGGCTAGCACATTGAGGGAAAGGGAATTGAGTGTGGTGAGGCAAAGCTTCCG | Negative | Negative |
| Arsenate | 10852725-SAH-2 | GGGCGAAUUCAGGACUCCUGACAUAUGAUGUUUUAUGGUUAUGCUCUGCGAAAUCACUGAUGAGACGCUUGGCGUGUGCUGUGGAGAGUCAUCAGUGCGCAGGAUCCAUC | Negative | Negative |
| Arsenate | 9245404-dopamine-1 | GGGAAUUCCGCGUGUGCGCCGCGGAAGACGUUGGAAGGAUAGAUACCUACAACGGGGAAUAUAGAGGCCAGCACAUAGUGAGGCCCUCCUCCCAAGGUCCGUUCGGGAUCCUC | Negative | Negative |
| Arsenate | 23042406-Neu5Ac-Ma-1 | GACGCUGGGCCUAGUCCGUGACUAUAUCGAUUGCCUUUUCAGACUUGCGUC | Negative | Negative |
| Arsenate | 23042406-Neu5Ac-3 | GCGGAAGCGUGCUGGGCCCGGGAAAUGGACCUAUAUCGUACCUUACAACUUGGUUAGAACAUAACCCAGAGGUCGAU | Negative | Negative |
| Arsenate | 11851395-FAD-1 | GGGCAUAAGGUAUUUAAUUCCAUACAAGUUUACAAGAAAGAUGC | Negative | Negative |
| Arsenate | 10913311-cAMP-4 | UAAAUCGACUUGUCGUAAACCCAUUCUUAUCGGCGUGCUCUACAGCCG | Negative | Negative |
| Glutamic acid | 9831529-sulforhodamine B-Ma-15 | CCGGCCAGGGTGGGAGGGAGGGGGCCGG | Negative | Negative |
| Glutamic acid | 9831529-sulforhodamine B-Ma-6 | CCGGCCAAAGGTGGGAGGGAGGGGGCCGG | Negative | Negative |
| Glutamic acid | 9831529-sulforhodamine B-Ma-7 | CCGGCCAAGGGTGGGAGGGAGAGGGCCGG | Negative | Negative |
| Glutamic acid | 17038331-codeine-6 | GGGACAGGGCTAGCAGTAGGATTGGGTGAGGGGATGTGCTGTGAGGCAAAGCTTCCG | Negative | Negative |
| Glutamic acid | 18983163-ochratoxin A-4 | TGGTGGCTGTAGGTCAGCACGATGGGGAAAGGGTCCCCCTGGGTTGGAGCATCGGACAACG | Negative | Negative |
| Glutamic acid | 11128644-Hematoporphyrin-3 | CAGCGTAAGGTCGTGGGTGGTTGGGTGCCTAAGCGTATACTTACAGCGATTGATTTGGTC | Negative | Negative |
| Glutamic acid | 11128644-Hematoporphyrin-7 | GCGGCAAATCAGCATACGAGTGTACAGGGACGGGACGGGTGGGTAAAAGGTGTCGCCTCAG | Negative | Negative |
| Glutamic acid | 23042406-Neu5Ac-3 | GCGGAAGCGUGCUGGGCCCGGGAAAUGGACCUAUAUCGUACCUUACAACUUGGUUAGAACAUAACCCAGAGGUCGAU | Negative | Negative |
| Glutamic acid | 20000526-arsenic-8 | TGGGGATTGTACGTACACCACCAATTCACACGCGAGTATG | Negative | Negative |
| Glutamic acid | 20000526-arsenic-Ma-1 | TTACAGAACAACCAACGTCGCTCCGGGTACTTCTTCATCG | Negative | Negative |
| Isoleucine | 18983163-ochratoxin A-19 | GATCGGGTGTGGGTGGCGTAAAGGGAGCAT | Negative | Negative |
| Isoleucine | 18983163-ochratoxin A-7 | TGGTGGCTGTAGGTCACCAAATGCGACGGGGCCTGTTTTAATGGGGGAGCATCGGACAACG | Negative | Negative |
| Isoleucine | 10786843-L Tyrosine-3 | AAGGGCAGUCCCCCCUUCGCUGGGGGGGUGGUUGUAGGGCUAAAACAAACCACGGGUGAUACGGGGGCCAUACCCUAGGAAGGCCCUGCUCCC | Negative | Negative |
| Isoleucine | 10786843-L Tyrosine-1 | GGCAGUCAACUCGUGCGAUCGUGAAAACGGGGCAAGAUGGCCUUACAGCGGUCAAUACGGGGGUCAUCAGAUAGGGAGGCCCUCCUGGU | Negative | Negative |
| Isoleucine | 10978501-cholic acid-3 | GCTTATTCAATTCGCGGAAGACGAATTCCAAGCGCGCGCGGGTCACGCGACTTGGGAATGAGC | Negative | Negative |
| Isoleucine | 20000526-arsenic-7 | ATGCAAACCCTTAAGAAAGTGGTCGTCCAAAAAACCATTG | Negative | Negative |
| Adenine | 9576904-cellobiose-1 | GCGGGGTTGGGCGGGTGGGTTCGCTGGGCAGGGGGCGAGTG | Negative | Negative |
| Adenine | 11128644-Hematoporphyrin-6 | CAATGGGGTCGGGCGGGCCGGGTGTCATGGTGGACGGAGATGGGACGTAGAGGGCGGT | Negative | Negative |
| Adenine | 18983163-ochratoxin A-19 | GATCGGGTGTGGGTGGCGTAAAGGGAGCAT | Negative | Negative |
| Adenine | 21076782-L-Tryptophan-Ma-1 | AGCACGTTGGTAGGTCGGTTTGGGTTTCGTGC | Negative | Negative |
| Adenine | 18983163-ochratoxin A-1 | TGGTGGCTGTAGGTCAGCATCTGATCGGGTGTGGGTGGCGTAAAGGGAGCATCGGACAACG | Negative | Negative |
| Adenine | 9889155-sulforhodamine B-2 | GGACGGCACCACGGUCGGAUCCGUGAGUUGUGACAAUUUAGCGGGUGGUAUUAGAGCCUACUGCCACAGCAAUAGGAUCGAUACAGAUCU | Negative | Negative |
| Adenine | 10743940-chitin-7 | TAGGGAATTCGTCGACGGATCCCGTACAGTGGGCTATATTGCTGACGTAAGAGCTGCTTTCAATGCATTGGCATAGATGTCACTCCAGGTCGACGCATGCGCCG | Negative | Negative |
| Adenine | 12873145-ATP-1 | GGCAGACGUAAGGCUGAGAUUGCCGUCCGUAAUUGAGUGCGGACGCGAGACGUCUGCC | Negative | Negative |
| Adenine | 20000526-arsenic-8 | TGGGGATTGTACGTACACCACCAATTCACACGCGAGTATG | Negative | Negative |
| Adenine | 10978501-cholic acid-4 | GAACATACGGCAGTTTATGGCCGCTATCGAGATAGACTATCATCTCAACGTCTTCTAGATAGTATGTTC | Negative | Negative |
| Adenine | 20000526-arsenic-6 | CAATCTAAGCGAACCGCGTGCAGACCAATTACTCGCGCTA | Negative | Negative |
| Dopamine | 9831529-sulforhodamine B-Ma-12 | CCGGCCAAGGGAGGGAGGGAGGGGGCCGG | Negative | Negative |
| Dopamine | 9831529-sulforhodamine B-Ma-17 | CCGGCCAAGGGTGGGAGGGAGGGAGGCCGG | Negative | Negative |
| Dopamine | 17038331-codeine-17 | GGGACAGGGCTAGCGCAGAACAGAGGGTAGGGAATTTGCGTGTGAGGCAAAGCTTCCG | Negative | Negative |
| Dopamine | 11128644-Hematoporphyrin-2 | ACGAAGAAACAGGGCGCTTCGAACGAACATGGGCAGGGTGGGAAGTTTTAAAGTGGTAC | Negative | Negative |
| Dopamine | 12873145-ATP-1 | GGCAGACGUAAGGCUGAGAUUGCCGUCCGUAAUUGAGUGCGGACGCGAGACGUCUGCC | Negative | Negative |
| Dopamine | 20000526-arsenic-4 | TTCCGCTAGGGGAACATGATCAACATGGACCAAGTAAAC | Negative | Negative |
| Dopamine | 10978501-cholic acid-2 | AGCGCCGATTGACCCAAATCGTTTTGTATGCAAAAGCGCT | Negative | Negative |
| Chitin | 9831529-sulforhodamine B-Ma-16 | CCGGCCGGGTGGGAGGGAGGGGGCCGG | Negative | Negative |
| Chitin | 18983163-ochratoxin A--Ma-16 | GATCGGGTGTGGGTGGCGTAAAGGGAGCATCGG | Negative | Negative |
| Chitin | 18983163-ochratoxin A-14 | TGGTGGCTGTAGGTCAGCAGTACGATCGGGGGTGGGTGGATGTAGGGAGCATCGGACAACG | Negative | Negative |
| Chitin | 18983163-ochratoxin A-2 | TGGTGGCTGTAGGTCAGGGGTGAAACGGGTCCCGGAGCATCGGACAACG | Negative | Negative |
| Chitin | 17038331-codeine-5 | GGGACAGGGCTAGCACATGGAGGCTTATAGGGATTCGTGCTGGGAGGCAAAGCTTCCG | Negative | Negative |
| Chitin | 17038331-codeine-1 | GGGACAGGGCTAGCAAAAGGGTGGTTGAAGGGACAGCTGGTGTGAGGCAAAGCTTCCG | Negative | Negative |
| Chitin | 17038331-codeine-12 | GGGACAGGGCTAGCTACTAATGTACGCACTAAGGGATTGGGGTGAGGCAAAGCTTCCG | Negative | Negative |
| Chitin | 8604334-LArginine-1 | AUGAUAAACCGAUGCUGGGCGAUUCUCCUGAAGUAGGGGAAGAGUUGUCAUGUAUGGG | Negative | Negative |
| Chitin | 10852725-SAH-1 | GGGCGAAUUCAGGACUCCUGACAUGUAGUUGAUUGAAGGACGUUUGAUUCUUAUGGAUUGUGGAAAGCUUCGGUCAUCAGUGCGCAGGAUCCAUC | Negative | Negative |
| Chitin | 22261866-pesticides-2 | AAGCTTTTTTGACTGACTGCAGCGATTCTTGATCGCCACGGTCTGGAAAAAGAG | Negative | Negative |
| Chitin | 20000526-arsenic-4 | TTCCGCTAGGGGAACATGATCAACATGGACCAAGTAAAC | Negative | Negative |
| Chitin | 20000526-arsenic-2 | CACGCGTTCAACCCCCGGAATTTAGCAATAGCAGATTACG | Negative | Negative |
| L-Arginine | 9831529-sulforhodamine B-Ma-5 | CCGGCCAAGGGGTGGGGAGGGGAGGGGGGCCGG | Negative | Negative |
| L-Arginine | 9831529-sulforhodamine B-Ma-16 | CCGGCCGGGTGGGAGGGAGGGGGCCGG | Negative | Negative |
| L-Arginine | 9831529-sulforhodamine B-Ma-10 | CCGGCCTAGGGTGGGAGGGAGGGGGCCGG | Negative | Negative |
| L-Arginine | 9831529-sulforhodamine B-Ma-11 | CCGGCCATGGGTGGGAGGGAGGGGGCCGG | Negative | Negative |
| L-Arginine | 11128644-Hematoporphyrin-Ma-2 | ATGGTGGACGGAGATGGGACGTAG | Negative | Negative |
| L-Arginine | 17038331-codeine-16 | GGGACAGGGCTAGCCTATAGTGAGGGTATTAAGGGTTGTGGGGGAGGCAAAGCTTCCG | Negative | Negative |
| L-Arginine | 18983163-ochratoxin A-2 | TGGTGGCTGTAGGTCAGGGGTGAAACGGGTCCCGGAGCATCGGACAACG | Negative | Negative |
| L-Arginine | 23042406-Neu5Ac-1 | GCGGAAGCGUGCUGGGCCUAGUCCGUGACUAUAUCGAUUGCCUUUCAGACUUGCAUAACCCAGAGGUCGAUCCUAGGGGGAGA | Negative | Negative |
| L-Arginine | 23042406-Neu5Ac-7 | GCGGAAGCGUGCUGGGCCGUUAUAACCGGGUUACUAUCUGCCAAGUAGUGGAAUUCCCAUAACCCAGAGGUCGAU | Negative | Negative |
| L-Arginine | 10743940-chitin-7 | TAGGGAATTCGTCGACGGATCCCGTACAGTGGGCTATATTGCTGACGTAAGAGCTGCTTTCAATGCATTGGCATAGATGTCACTCCAGGTCGACGCATGCGCCG | Negative | Negative |
| L-Arginine | 20000526-arsenic-5 | CAGTCAGAATCCGGGCTTACACCCATTTGTTTATTGTGCA | Negative | Negative |
| L-Arginine | 22261866-pesticides-2 | AAGCTTTTTTGACTGACTGCAGCGATTCTTGATCGCCACGGTCTGGAAAAAGAG | Negative | Negative |
| L-Arginine | 10978501-cholic acid-4 | GAACATACGGCAGTTTATGGCCGCTATCGAGATAGACTATCATCTCAACGTCTTCTAGATAGTATGTTC | Negative | Negative |
| L-Arginine | 10913311-cAMP-4 | UAAAUCGACUUGUCGUAAACCCAUUCUUAUCGGCGUGCUCUACAGCCG | Negative | Negative |
| L-Arginine | 10913311-cAMP-2 | UAAAACGACUUGUCGCGUGCUGCCCGCCUGUUCGCUUUCUGCACCCC | Negative | Negative |
| Cellobiose | 9831529-sulforhodamine B-Ma-4 | CCGGCCAAGGGTGGGAGGGAGGGGGCCGG | Negative | Negative |
| Cellobiose | 9831529-sulforhodamine B-Ma-3 | ATGACCAAGGGTGGGAGGGAGGGGGTCAT | Negative | Negative |
| Cellobiose | 18983163-ochratoxin A-20 | GATCGGGTGTGGGTGGCGTAAAGGGAG | Negative | Negative |
| Cellobiose | 11128644-Hematoporphyrin-6 | CAATGGGGTCGGGCGGGCCGGGTGTCATGGTGGACGGAGATGGGACGTAGAGGGCGGT | Negative | Negative |
| Cellobiose | 11128644-Hematoporphyrin-8 | CCAACTTGGGCGGAGGGCTAACGGTGGGGGGATATTATGAGGGGTGGAGGTATTAACCATT | Negative | Negative |
| Cellobiose | 17038331-codeine-9 | GGGACAGGGCTAGCAGGGTAAGGGGATTGGGAGTAGTGCCGTGTGAGGCAAAGCTTCCG | Negative | Negative |
| Cellobiose | 17038331-codeine-5 | GGGACAGGGCTAGCACATGGAGGCTTATAGGGATTCGTGCTGGGAGGCAAAGCTTCCG | Negative | Negative |
| Cellobiose | 21413891-Bisphenol A-1 | CCGGTGGGTGGTCAGGTGGGATAGCGTTCCGCGTATGGCCCAGCGCATCACGGGTTCGCACCA | Negative | Negative |
| Cellobiose | 11128644-Hematoporphyrin-7 | GCGGCAAATCAGCATACGAGTGTACAGGGACGGGACGGGTGGGTAAAAGGTGTCGCCTCAG | Negative | Negative |
| Cellobiose | 20000526-arsenic-5 | CAGTCAGAATCCGGGCTTACACCCATTTGTTTATTGTGCA | Negative | Negative |
| Cellobiose | 10339553-MalachiteGreen-1 | GGAACACUAUCCGACUGGCACCCCCCUGCCAGGUAACGAAUGAAGUGCUUUUCUCGAUCUCGUGACCCGCGCACUAGUCGCGAAGGUGUAUGUCCUUGGUCAUUAGGAUCCCG | Negative | Negative |
| Cellobiose | 11705996-adenine-Ma-1 | GGGACGAUUAUCGAAAAUCACCAGAUUGGACCCUGGUUAACGAUCCC | Negative | Negative |
| Cellobiose | 11705996-adenine-1 | ATAGGACGATTATCGAAAATCACCAGATTGGACCCTGGTTAACGAT | Negative | Negative |
| Cellobiose | 10913311-cAMP-1 | CCGACGCGUAAACGAAACGCACAUUCCCCCACAGGAUAUGUGCUCGG | Negative | Negative |
| Tyrosine | 9831529-sulforhodamine B-Ma-20 | CCGGCCAAGGGTGGGAAGGAGGGGGCCGG | Negative | Negative |
| Tyrosine | 9831529-sulforhodamine B-Ma-19 | CCGGCCAAGGGTGGAAGGGAGGGGGCCGG | Negative | Negative |
| Tyrosine | 17038331-codeine-19 | GGGACAGGGCTAGCAGCGGTAAGGGTGGGGAGAATGGTGCTGTGAGGCAAAGCTTCCG | Negative | Negative |
| Tyrosine | 17038331-codeine-18 | GGGACAGGGCTAGCATCGGTGTAGGGAAGGGATATGATGTGGTGAGGCAAAGCTTCCG | Negative | Negative |
| Tyrosine | 18983163-ochratoxin A-3 | TGGTGGCTGTAGGTCAGCAGTCCTAGATCGGGTGTGGCTGGCTTGGGAGCATCGGACAACG | Negative | Negative |
| Tyrosine | 17038331-codeine-20 | GGGACAGGGCTAGCAAAGGTTAAGGGTAGGGGATACTGCTGGTGAGGCAAAGCTTCCG | Negative | Negative |
| Tyrosine | 18983163-ochratoxin A-10 | TGGTGGCTGTAGGTCAACATGCGACTGAGGCTCGGTTTATTGAGGGGAGCATCGGACAACG | Negative | Negative |
| Tyrosine | 17038331-codeine-17 | GGGACAGGGCTAGCGCAGAACAGAGGGTAGGGAATTTGCGTGTGAGGCAAAGCTTCCG | Negative | Negative |
| Tyrosine | 17038331-codeine-11 | GGGACAGGGCTAGCCAATAAATAAGGCGAAGTAAGGGATGGGGTGAGGCAAAGCTTCCG | Negative | Negative |
| Tyrosine | 10743940-chitin-5 | TAGGGAATTCGTCGACGGATCCCCGTAACCCTGCGGGGGGGGGAGAAGGCAATGGGGGACAACTCGCCGGTAGCCATCCATATCTCCAGGTCGACGC-ATGCGCCG | Negative | Negative |
| Tyrosine | 10743940-chitin-6 | TAGGGAATTCGTCGACGGATCCCCTAAGGGGGGACTCAGCATTTTGTGCGGGCGGCGCTAACACAATCAGATAGAGCGGGGTTCTCCAGGTCGACGC-ATGCGCCG | Negative | Negative |
| Tyrosine | 9245404-Dopamine-6 | GGGAAUUCCGCGUGUGCCUUUCCAUGUAUUAGAGAGGAGAGCGCAAACACGAAGGCUCGUCGUUACAGCCGAAGAGCUCCAGUACCAUGGUUCAUGAGUCCGUUCGGGAUCCUC | Negative | Negative |
| Tyrosine | 11851395-FAD-1 | GGGCAUAAGGUAUUUAAUUCCAUACAAGUUUACAAGAAAGAUGC | Negative | Negative |
| Tyrosine | 20000526-arsenic-6 | CAATCTAAGCGAACCGCGTGCAGACCAATTACTCGCGCTA | Negative | Negative |
| Omethoate | 11128644-Hematoporphyrin-Ma-1 | ATGGGGTCGGGCGGGCCGGGTGTC | Negative | Negative |
| Omethoate | 9831529-sulforhodamine B-Ma-13 | CCGGCCAAGGGTGGGTGGGAGGGGGCCGG | Negative | Negative |
| Omethoate | 18983163-ochratoxin A-21 | GATCGGGTGTGGGTGGCGTAAAGG | Negative | Negative |
| Omethoate | 18983163-ochratoxin A-24 | TCGGGTGTGGGTGGCGTAAAGGGAGCATCGG | Negative | Negative |
| Omethoate | 18983163-ochratoxin A-25 | ATCGGGTGTGGGTGGCGTAAAG | Negative | Negative |
| Omethoate | 17038331-codeine-14 | GGGACAGGGCTAGCTTAGTGCTATGTGAGAAAAGGGTGTGGGGGAGGCAAAGCTTCCG | Negative | Negative |
| Omethoate | 18983163-ochratoxin A-4 | TGGTGGCTGTAGGTCAGCACGATGGGGAAAGGGTCCCCCTGGGTTGGAGCATCGGACAACG | Negative | Negative |
| Omethoate | 18983163-ochratoxin A-23 | GATCGGGTGTCGGTGGCGTAAAGGGAGCATCGGACAACG | Negative | Negative |
| Omethoate | 17038331-codeine-11 | GGGACAGGGCTAGCCAATAAATAAGGCGAAGTAAGGGATGGGGTGAGGCAAAGCTTCCG | Negative | Negative |
| Omethoate | 10743940-chitin-6 | TAGGGAATTCGTCGACGGATCCCCTAAGGGGGGACTCAGCATTTTGTGCGGGCGGCGCTAACACAATCAGATAGAGCGGGGTTCTCCAGGTCGACGC-ATGCGCCG | Negative | Negative |
| Omethoate | 9245404-Dopamine-5 | GGGAAUUCCGCGUGUGCACAGCCAGGGUAAGAGUCUAGAGUAGUUCAUUCCUAGUAUCUGGAGGCUGGUACUAUAAGGGUAGUACUGCCCGCGUACUGUCCGUUCGGGAUCCUC | Negative | Negative |
| Omethoate | 23042406-Neu5Ac-1 | GCGGAAGCGUGCUGGGCCUAGUCCGUGACUAUAUCGAUUGCCUUUCAGACUUGCAUAACCCAGAGGUCGAUCCUAGGGGGAGA | Negative | Negative |
| Omethoate | 11101810-ATP-1 | GAAGCAAGCAGGCAACGAACACAGAAGACCGGGGGAACUACCGCGCGUGCC | Negative | Negative |
| N-Acetylneuraminic acid | 9831529-sulforhodamine B-Ma-12 | CCGGCCAAGGGAGGGAGGGAGGGGGCCGG | Negative | Negative |
| N-Acetylneuraminic acid | 17038331-codeine-10 | GGGACAGGGCTAGCGGACAAGAAGTGGGTAAGGGAATCCGTGGGAGGCAAAGCTTCCG | Negative | Negative |
| N-Glycolylneuraminic acid | 9831529-sulforhodamine B-Ma-13 | CCGGCCAAGGGTGGGTGGGAGGGGGCCGG | Negative | Negative |
| N-Glycolylneuraminic acid | 9831529-sulforhodamine B-Ma-9 | CCGGCAAAGGGTGGGAGGGAGGGCGCCGG | Negative | Negative |
| N-Glycolylneuraminic acid | 18983163-ochratoxin A-22 | ATCGGGTGTGGGTGGCGTAAAGGGAGCATCGG | Negative | Negative |
| N-Glycolylneuraminic acid | 9831529-sulforhodamine B-Ma-18 | CCGGCCAAGGTGGAGGAGGGGCCGG | Negative | Negative |
| N-Glycolylneuraminic acid | 17038331-codeine-9 | GGGACAGGGCTAGCAGGGTAAGGGGATTGGGAGTAGTGCCGTGTGAGGCAAAGCTTCCG | Negative | Negative |
| N-Glycolylneuraminic acid | 17038331-codeine-19 | GGGACAGGGCTAGCAGCGGTAAGGGTGGGGAGAATGGTGCTGTGAGGCAAAGCTTCCG | Negative | Negative |
| N-Glycolylneuraminic acid | 9831529-sulforhodamine B-Ma-2 | CGGGATCCTAATGACCAAGGGTGGGAGGGAGGGGGTCATTAAATCCAG | Negative | Negative |
| N-Glycolylneuraminic acid | 17038331-codeine-2 | GGGACAGGGCTAGCACAAGAATTAGGGTCGGGAAATGGTGTGTGAGGCAAAGCTTCCG | Negative | Negative |
| N-Glycolylneuraminic acid | 11128644-Hematoporphyrin-1 | TATGAACCAAGGGAGGGGCAGGGCGGGTAGGGTTAATAACGGATTCCGAGCGCCCAGCTC | Negative | Negative |
| N-Glycolylneuraminic acid | 10978501-cholic acid-1 | GATCGAGGGCAGCGATAGCTGGGCTAATAAGGTTAGCCCCATCGGTC | Negative | Negative |
| N-Glycolylneuraminic acid | 18187867-Glu-2 | CTGAGCCTGCGTGTATAGGACATTGACGGCGGTCGAAGCGGAGGCACGTAGCCAGGCAGAGGGACAGGTGGAGATAGCAGTATCGTCATGATCCATCG | Negative | Negative |
| Bisphenol A | 9889155-sulforhodamine B-Ma-1 | AUGGAGGGGCGCAAG | Negative | Negative |
| Bisphenol A | 17038331-codeine-16 | GGGACAGGGCTAGCCTATAGTGAGGGTATTAAGGGTTGTGGGGGAGGCAAAGCTTCCG | Negative | Negative |
| Bisphenol A | 10786843-L Tyrosine-2 | GAGCGACUGAGGUUCGCCGCGGAUUAUGUUUUGCGGUUAGAUCAGGCAACGGGUAAUACCGGGUCAGUCAGAUAGGGAGGAUCUACUGCC | Negative | Negative |
| Bisphenol A | 10852725-SAH-1 | GGGCGAAUUCAGGACUCCUGACAUGUAGUUGAUUGAAGGACGUUUGAUUCUUAUGGAUUGUGGAAAGCUUCGGUCAUCAGUGCGCAGGAUCCAUC | Negative | Negative |
| Bisphenol A | 9831529-sulforhodamine B-1 | CGGGATCCTAATGACCAAGGGTGGGAGGGAGGGGGTCATTAAATCCAGTATCAACACGCCACGATGGGATCACCGCCATGGGCCGTCCCACTGGTGCCAGTCGGATAGTGTTCCTATAGTGAGTCGTATTAGAA | Negative | Negative |
| Bisphenol A | 18187867-Glu-2 | CTGAGCCTGCGTGTATAGGACATTGACGGCGGTCGAAGCGGAGGCACGTAGCCAGGCAGAGGGACAGGTGGAGATAGCAGTATCGTCATGATCCATCG | Negative | Negative |
| Tryptophan | 9831529-sulforhodamine B-Ma-11 | CCGGCCATGGGTGGGAGGGAGGGGGCCGG | Negative | Negative |
| Tryptophan | 9831529-sulforhodamine B-Ma-14 | CCGGCCAAGGGTGGGAGGGTGGGGGCCGG | Negative | Negative |
| Tryptophan | 9831529-sulforhodamine B-Ma-20 | CCGGCCAAGGGTGGGAAGGAGGGGGCCGG | Negative | Negative |
| Tryptophan | 23042406-Neu5Ac-1 | GCGGAAGCGUGCUGGGCCUAGUCCGUGACUAUAUCGAUUGCCUUUCAGACUUGCAUAACCCAGAGGUCGAUCCUAGGGGGAGA | Negative | Negative |
| Tryptophan | 10978501-cholic acid-Ma-1 | GTACCAGCTTATTGCTGCGGGCTGAAGCCCGGTAC | Negative | Negative |
| Tryptophan | 14980623-sialyllactose-1 | AGCGGGGCCAGCCACTTCTGTCAGTGAATTCCTGCTCGTATATCTACTCGCCCGCCTGCG | Negative | Negative |
| Tryptophan | 11101810-ATP-1 | GAAGCAAGCAGGCAACGAACACAGAAGACCGGGGGAACUACCGCGCGUGCC | Negative | Negative |
| Codeine | 14561881A-isoleucine-3 | RCGACCGUAUGMGAAAGUUGGG | Negative | Negative |
| Codeine | 18983163-ochratoxin A-14 | TGGTGGCTGTAGGTCAGCAGTACGATCGGGGGTGGGTGGATGTAGGGAGCATCGGACAACG | Negative | Negative |
| Codeine | 18983163-ochratoxin A-11 | TGGTGGCTGTAGGTCACCTGACGATCGGGTGTGGGTTGGCTTGAGGGAGCATCGGACAACG | Negative | Negative |
| Codeine | 18983163-ochratoxin A-12 | TGGTGGCTGTAGGTCACCTTGTAGATCGGGTGTGGTTTGGCGTAGGGAGCATCGGACAACG | Negative | Negative |
| Codeine | 18983163-ochratoxin A-1 | TGGTGGCTGTAGGTCAGCATCTGATCGGGTGTGGGTGGCGTAAAGGGAGCATCGGACAACG | Negative | Negative |
| Codeine | 18983163-ochratoxin A-10 | TGGTGGCTGTAGGTCAACATGCGACTGAGGCTCGGTTTATTGAGGGGAGCATCGGACAACG | Negative | Negative |
| Codeine | 9831529-sulforhodamine B-1 | CGGGATCCTAATGACCAAGGGTGGGAGGGAGGGGGTCATTAAATCCAGTATCAACACGCCACGATGGGATCACCGCCATGGGCCGTCCCACTGGTGCCAGTCGGATAGTGTTCCTATAGTGAGTCGTATTAGAA | Negative | Negative |
| Codeine | 9245404-Dopamine-9 | GGGAAUUCCGCGUGUGCUUAACCUAAUGGAAUACAAUUCUAACAGGAAUAGGAGGGUAGCUCCAAGUACUAACGACCGUUCCGGUAGCCGGCUGGCUGUCCGUUCGGGAUCCUC | Negative | Negative |
| Codeine | 10978501-cholic acid-5 | GCAGGGTCAATGGAATTAATGATCAATTGACAGACGCAAGTCTCCTGC | Negative | Negative |
| Codeine | 21614178-fumonisin B1-1 | ATACCAGCTTATTCAATTAATCGCATTACCTTATACCAGCTTATTCAATTACGTCTGCACATACCAGCTTATTCAATTAGATAGTAAGTGCAATCT | Negative | Negative |
| Acetamiprid | 18983163-ochratoxin A-11 | TGGTGGCTGTAGGTCACCTGACGATCGGGTGTGGGTTGGCTTGAGGGAGCATCGGACAACG | Negative | Negative |
| Acetamiprid | 18983163-ochratoxin A-12 | TGGTGGCTGTAGGTCACCTTGTAGATCGGGTGTGGTTTGGCGTAGGGAGCATCGGACAACG | Negative | Negative |
| Acetamiprid | 18983163-ochratoxin A-6 | TGGTGGCTGTAGGTCATCAGTCCCGATCAGGTGTGGGTGGCATTGGGAGCATCGGACAACG | Negative | Negative |
| Acetamiprid | 17038331-codeine-8 | GGGACAGGGCTAGCACCAAAAATAGGGGTAAGGGCATGGGGGTGAGGCAAAGCTTCCG | Negative | Negative |
| Acetamiprid | 17038331-codeine-10 | GGGACAGGGCTAGCGGACAAGAAGTGGGTAAGGGAATCCGTGGGAGGCAAAGCTTCCG | Negative | Negative |
| Acetamiprid | 17038331-codeine-15 | GGGACAGGGCTAGCGTAATAAGTAGGGAAAGGGTTCCCGCTGGGAGGCAAAGCTTCCG | Negative | Negative |
| Acetamiprid | 10743940-chitin-1 | TAGGGAATTCGTCGACGGATCCCCCAGCAGCACTGGTAGTGAGGCAGTTCACCGGTGGGGCGGTGAGTTTGGCTGCTATTTATCTCCAGGTCGACGC-ATGCGCCG | Negative | Negative |
| Acetamiprid | 9245404-Dopamine-4 | GGGAAUUCCGCGUGUGCCAGCCACGUAGAAUCGUCUCUGCGCCGGGGAGAGUGUCAUGGUUAACGGGUAACAGCUCGUGGAGACGACCUCGGUUUCGGUCCGUUCGGGAUCCUC | Negative | Negative |
| Acetamiprid | 9245404-dopamine-Ma-1 | GGGAAUUCCGCGUGUGCGCCGCGGAAGACGUUGGAAGGAUAGAUACCUACAACGGGGAAUAUAGAGGCCAGCACAUAGUGAGGCCCUCCUCCC | Negative | Negative |
| Acetamiprid | 10978501-cholic acid-3 | GCTTATTCAATTCGCGGAAGACGAATTCCAAGCGCGCGCGGGTCACGCGACTTGGGAATGAGC | Negative | Negative |
| Acetamiprid | 20000526-arsenic-2 | CACGCGTTCAACCCCCGGAATTTAGCAATAGCAGATTACG | Negative | Negative |
| Phorate | 9576904-cellobiose-1 | GCGGGGTTGGGCGGGTGGGTTCGCTGGGCAGGGGGCGAGTG | Negative | Negative |
| Phorate | 11128644-Hematoporphyrin-Ma-1 | ATGGGGTCGGGCGGGCCGGGTGTC | Negative | Negative |
| Phorate | 11128644-Hematoporphyrin-4 | GGTGCGGGTAGGGATGAGGGCGGGTCGGGTTGAGTGTGAGCTTAGGGGTGGCGGATCTAC | Negative | Negative |
| Phorate | 9831529-sulforhodamine B-Ma-8 | CCGGCCAAGGGTGGGAGGGAAGGGGCCGG | Negative | Negative |
| Phorate | 18983163-ochratoxin A-20 | GATCGGGTGTGGGTGGCGTAAAGGGAG | Negative | Negative |
| Phorate | 21076782-L-Tryptophan-1 | CAATGGCAGGCACGTTGGTTAGGTCAGGTTT-GGGTTTCGTGCTT | Negative | Negative |
| Phorate | 18983163-ochratoxin A-8 | TGGTGGCTGTAGGTCACGTACGGTGGGAACGGTTCCTCTTAGGGTGAGCATCGGACAACG | Negative | Negative |
| Phorate | 17038331-codeine-7 | GGGACAGGGCTAGCACATTGAGGGAAAGGGAATTGAGTGTGGTGAGGCAAAGCTTCCG | Negative | Negative |
| Phorate | 10786843-L Tyrosine-2 | GAGCGACUGAGGUUCGCCGCGGAUUAUGUUUUGCGGUUAGAUCAGGCAACGGGUAAUACCGGGUCAGUCAGAUAGGGAGGAUCUACUGCC | Negative | Negative |
| Phorate | 9889155-sulforhodamine B-1 | GAAGUUUUACCGUGUUGGUGGUCUAUGAGCCUACUGCCUGCAGUACAUGAGGUAUCGG | Negative | Negative |
| Phorate | 9245404-Dopamine-7 | GGGAAUUCCGCGUGUGCAUGGUUAAUGGAAUCCUGGGCUCCGGAGAUCACCCAUCGGAGGUAAUUUCUUUAGAAUGCUAGACGGACACUACCAGCGUGUCCGUUCGGGAUCCUC | Negative | Negative |
| Phorate | 10743940-chitin-3 | TAGGGAATTCGTCGACGGATCCGGCAAGATGTGCCCAGAACAGTTGCTTGTATGGTGGGGAGCGTCCATATTGGCTTAAACCTCCAGGTCGACGCATGCGCCG | Negative | Negative |
| Phorate | 21306108-Acetamiprid-1 | TGTAATTTGTCTGCAGCGGTTCTTGATCGCTGACACCATATTATGAAGA | Negative | Negative |
| Phorate | 9245404-Dopamine-8 | GGGAAUUCCGCGUGUGCUAAGUACUUGGCGGGAUAACGUCCAAGAGACGAGAGUCACGCACCUUAAACAGCUGUACUACGUGCCCCCGUAGGUGUCCGUUCGGGAUCCUC | Negative | Negative |
| Phorate | 20000526-arsenic-3 | TTACAGAACAACCAACGTCGCTCCGGGTACTTCTTCATCG | Negative | Negative |
| Cholic acid | 10743940-chitin-3 | TAGGGAATTCGTCGACGGATCCGGCAAGATGTGCCCAGAACAGTTGCTTGTATGGTGGGGAGCGTCCATATTGGCTTAAACCTCCAGGTCGACGCATGCGCCG | Negative | Negative |
| Cholic acid | 23042406-Neu5Ac-2 | GCGGAAGCGUGCUGGGCCACUAUAUCGAUGCCUUUCAGACUUGGCAUAACCCAGAGGUCGAU | Negative | Negative |
| Cholic acid | 10339553-MalachiteGreen-1 | GGAACACUAUCCGACUGGCACCCCCCUGCCAGGUAACGAAUGAAGUGCUUUUCUCGAUCUCGUGACCCGCGCACUAGUCGCGAAGGUGUAUGUCCUUGGUCAUUAGGAUCCCG | Negative | Negative |
| Cyclic adenosine monophosphate | 9831529-sulforhodamine B-Ma-21 | CCGGCCAAGGGTGGGAGGGAGGAGGCCGG | Negative | Negative |
| Cyclic adenosine monophosphate | 17038331-codeine-Ma-1 | GGGACAGGGCUAGCAGUAGGAUUGGGUGAGGGGAUGUGCUG | Negative | Negative |
| Cyclic adenosine monophosphate | 11128644-Hematoporphyrin-8 | CCAACTTGGGCGGAGGGCTAACGGTGGGGGGATATTATGAGGGGTGGAGGTATTAACCATT | Negative | Negative |
| Cyclic adenosine monophosphate | 17038331-codeine-6 | GGGACAGGGCTAGCAGTAGGATTGGGTGAGGGGATGTGCTGTGAGGCAAAGCTTCCG | Negative | Negative |
| Cyclic adenosine monophosphate | 18983163-ochratoxin A-7 | TGGTGGCTGTAGGTCACCAAATGCGACGGGGCCTGTTTTAATGGGGGAGCATCGGACAACG | Negative | Negative |
| Cyclic adenosine monophosphate | 23042406-Neu5Ac-4 | GCGGAAGCGUGCUGGGCCGACAUGAAUGGCACUAGAUCGACGUUAGCGAUGUGAUGAUCAUAACCCAGAGGUCGAU | Negative | Negative |
| S-Adenosyl-L-homocysteine | 9576904-cellobiose-3 | GTCAAGGTGGGTGGGTGGGGTTGGTTGTTGTTTTGA | Negative | Negative |
| S-Adenosyl-L-homocysteine | 9831529-sulforhodamine B-Ma-14 | CCGGCCAAGGGTGGGAGGGTGGGGGCCGG | Negative | Negative |
| S-Adenosyl-L-homocysteine | 9831529-sulforhodamine B-Ma-6 | CCGGCCAAAGGTGGGAGGGAGGGGGCCGG | Negative | Negative |
| S-Adenosyl-L-homocysteine | 9831529-sulforhodamine B-Ma-8 | CCGGCCAAGGGTGGGAGGGAAGGGGCCGG | Negative | Negative |
| S-Adenosyl-L-homocysteine | 18983163-ochratoxin A-21 | GATCGGGTGTGGGTGGCGTAAAGG | Negative | Negative |
| S-Adenosyl-L-homocysteine | 18983163-ochratoxin A--Ma-16 | GATCGGGTGTGGGTGGCGTAAAGGGAGCATCGG | Negative | Negative |
| S-Adenosyl-L-homocysteine | 17038331-codeine-Ma-2 | GGGACAGGGCUAGCUUAGUGCUAUGUGAGAAAAGGGUGUGGGGG | Negative | Negative |
| S-Adenosyl-L-homocysteine | 18983163-ochratoxin A-Ma-17 | GATCGGGTGTGGGTGGCGTAAAGGGAGCATCG | Negative | Negative |
| S-Adenosyl-L-homocysteine | 18983163-ochratoxin A-Ma-13 | GATTCGGGTGTGGGTGGCGTAAAGGGAGCATCGGACAACG | Negative | Negative |
| S-Adenosyl-L-homocysteine | 17038331-codeine-14 | GGGACAGGGCTAGCTTAGTGCTATGTGAGAAAAGGGTGTGGGGGAGGCAAAGCTTCCG | Negative | Negative |
| S-Adenosyl-L-homocysteine | 17038331-codeine-3 | GGGACAGGGCTAGCCACAAGTGTGAAGGGATGGGAGTAGTGGTGAGGCAAAGCTTCCG | Negative | Negative |
| S-Adenosyl-L-homocysteine | 17038331-codeine-15 | GGGACAGGGCTAGCGTAATAAGTAGGGAAAGGGTTCCCGCTGGGAGGCAAAGCTTCCG | Negative | Negative |
| S-Adenosyl-L-homocysteine | 17038331-codeine-13 | GGGACAGGGCTAGCTACAGAATAAGCGAATTAAGGGTTGGGGTGAGGCAAAGCTTCCG | Negative | Negative |
| S-Adenosyl-L-homocysteine | 10743940-chitin-1 | TAGGGAATTCGTCGACGGATCCCCCAGCAGCACTGGTAGTGAGGCAGTTCACCGGTGGGGCGGTGAGTTTGGCTGCTATTTATCTCCAGGTCGACGC-ATGCGCCG | Negative | Negative |
| S-Adenosyl-L-homocysteine | 9889155-sulforhodamine B-1 | GAAGUUUUACCGUGUUGGUGGUCUAUGAGCCUACUGCCUGCAGUACAUGAGGUAUCGG | Negative | Negative |
| S-Adenosyl-L-homocysteine | 10743940-chitin-4 | TAGGGAATTCGTCGACGGATCCACCTGCGATAGGTAGTTTGTTTCTGTTGCCCTAGCGTCCGTCGTAACAAGTCACTCCAGGTCGACGCATGCGCCG | Negative | Negative |
| S-Adenosyl-L-homocysteine | 11705996-adenine-Ma-1 | GGGACGAUUAUCGAAAAUCACCAGAUUGGACCCUGGUUAACGAUCCC | Negative | Negative |
| S-Adenosyl-L-homocysteine | 11705996-adenine-1 | ATAGGACGATTATCGAAAATCACCAGATTGGACCCTGGTTAACGAT | Negative | Negative |
| Sialyllactose | 9831529-sulforhodamine B-Ma-15 | CCGGCCAGGGTGGGAGGGAGGGGGCCGG | Negative | Negative |
| Sialyllactose | 9831529-sulforhodamine B-Ma-10 | CCGGCCTAGGGTGGGAGGGAGGGGGCCGG | Negative | Negative |
| Sialyllactose | 17038331-codeine-Ma-2 | GGGACAGGGCUAGCUUAGUGCUAUGUGAGAAAAGGGUGUGGGGG | Negative | Negative |
| Sialyllactose | 17163839-Thyroxine-Ma-1 | GGUGGAGGGGGACGUGCUGCAUCCGCAGUGCGUCUUGGGUUGUG | Negative | Negative |
| Sialyllactose | 11128644-Hematoporphyrin-9 | CCGGGGATTAGAATAGTGGAGGGCCGGTGGCAAATGGGTAAGTAGGTTGAAGGGCTAAATT | Negative | Negative |
| Sialyllactose | 18983163-ochratoxin A-Ma-13 | GATTCGGGTGTGGGTGGCGTAAAGGGAGCATCGGACAACG | Negative | Negative |
| Sialyllactose | 17038331-codeine-20 | GGGACAGGGCTAGCAAAGGTTAAGGGTAGGGGATACTGCTGGTGAGGCAAAGCTTCCG | Negative | Negative |
| Sialyllactose | 17038331-codeine-1 | GGGACAGGGCTAGCAAAAGGGTGGTTGAAGGGACAGCTGGTGTGAGGCAAAGCTTCCG | Negative | Negative |
| Sialyllactose | 11128644-Hematoporphyrin-5 | CTAGAGTAACCTGTTGGGAGGGCGGGTAGGGCCCATTGAGAGGAGGACGTATTCGTCCGC | Negative | Negative |
| Sialyllactose | 11128644-Hematoporphyrin-1 | TATGAACCAAGGGAGGGGCAGGGCGGGTAGGGTTAATAACGGATTCCGAGCGCCCAGCTC | Negative | Negative |
| Sialyllactose | 21413891-Bisphenol A-1 | CCGGTGGGTGGTCAGGTGGGATAGCGTTCCGCGTATGGCCCAGCGCATCACGGGTTCGCACCA | Negative | Negative |
| Sialyllactose | 10786843-L Tyrosine-1 | GGCAGUCAACUCGUGCGAUCGUGAAAACGGGGCAAGAUGGCCUUACAGCGGUCAAUACGGGGGUCAUCAGAUAGGGAGGCCCUCCUGGU | Negative | Negative |
| Sialyllactose | 10852725-SAH-2 | GGGCGAAUUCAGGACUCCUGACAUAUGAUGUUUUAUGGUUAUGCUCUGCGAAAUCACUGAUGAGACGCUUGGCGUGUGCUGUGGAGAGUCAUCAGUGCGCAGGAUCCAUC | Negative | Negative |
| Sialyllactose | 9245404-dopamine-1 | GGGAAUUCCGCGUGUGCGCCGCGGAAGACGUUGGAAGGAUAGAUACCUACAACGGGGAAUAUAGAGGCCAGCACAUAGUGAGGCCCUCCUCCCAAGGUCCGUUCGGGAUCCUC | Negative | Negative |
| Sialyllactose | 15772067-isoleucine-1 | GGAUUGUGUUCCGGUAAUGAAACGAGUAAAAGAGCAAGUCC | Negative | Negative |
| Sialyllactose | 10913311-cAMP-1 | CCGACGCGUAAACGAAACGCACAUUCCCCCACAGGAUAUGUGCUCGG | Negative | Negative |
| Sialyllactose | 20000526-arsenic-1 | ACCATCGCGGAAGTCCAGTCTGCCATCAAAATCCGAAGTG | Negative | Negative |
| Adenosine triphosphate | 9576904-cellobiose-2 | TAGCGGGTGTGGTGGGTGGGGGAGGCATGGTTTTTGGTAA | Negative | Negative |
| Adenosine triphosphate | 9831529-sulforhodamine B-Ma-4 | CCGGCCAAGGGTGGGAGGGAGGGGGCCGG | Negative | Negative |
| Adenosine triphosphate | 17038331-codeine-Ma-1 | GGGACAGGGCUAGCAGUAGGAUUGGGUGAGGGGAUGUGCUG | Negative | Negative |
| Adenosine triphosphate | 18983163-ochratoxin A-Ma-18 | GATCGGGTGTGGGTGGCGTAAAGGGAGCATC | Negative | Negative |
| Adenosine triphosphate | 14561881A-isoleucine-3 | RCGACCGUAUGMGAAAGUUGGG | Negative | Negative |
| Adenosine triphosphate | 17038331-codeine-18 | GGGACAGGGCTAGCATCGGTGTAGGGAAGGGATATGATGTGGTGAGGCAAAGCTTCCG | Negative | Negative |
| Adenosine triphosphate | 18983163-ochratoxin A-23 | GATCGGGTGTCGGTGGCGTAAAGGGAGCATCGGACAACG | Negative | Negative |
| Adenosine triphosphate | 11128644-Hematoporphyrin-5 | CTAGAGTAACCTGTTGGGAGGGCGGGTAGGGCCCATTGAGAGGAGGACGTATTCGTCCGC | Negative | Negative |
| Adenosine triphosphate | 17038331-codeine-3 | GGGACAGGGCTAGCCACAAGTGTGAAGGGATGGGAGTAGTGGTGAGGCAAAGCTTCCG | Negative | Negative |
| Adenosine triphosphate | 10978501-cholic acid-6 | GTACCAGCTTATTCAATTACACGGACAGAGGGTAGCGGCTCTGCGCATTGAGTTGCTGCGGGCTGAAGCCCGGTAC | Negative | Negative |
| Adenosine triphosphate | 23042406-Neu5Ac-7 | GCGGAAGCGUGCUGGGCCGUUAUAACCGGGUUACUAUCUGCCAAGUAGUGGAAUUCCCAUAACCCAGAGGUCGAU | Negative | Negative |
| Adenosine triphosphate | 22261866-pesticides-2 | AAGCTTTTTTGACTGACTGCAGCGATTCTTGATCGCCACGGTCTGGAAAAAGAG | Negative | Negative |
| Ochratoxin A | 17038331-codeine-12 | GGGACAGGGCTAGCTACTAATGTACGCACTAAGGGATTGGGGTGAGGCAAAGCTTCCG | Negative | Negative |
| Ochratoxin A | 10743940-chitin-5 | TAGGGAATTCGTCGACGGATCCCCGTAACCCTGCGGGGGGGGGAGAAGGCAATGGGGGACAACTCGCCGGTAGCCATCCATATCTCCAGGTCGACGC-ATGCGCCG | Negative | Negative |
| Ochratoxin A | 10978501-cholic acid-Ma-1 | GTACCAGCTTATTGCTGCGGGCTGAAGCCCGGTAC | Negative | Negative |
| Ochratoxin A | 21614178-fumonisin B1-1 | ATACCAGCTTATTCAATTAATCGCATTACCTTATACCAGCTTATTCAATTACGTCTGCACATACCAGCTTATTCAATTAGATAGTAAGTGCAATCT | Negative | Negative |
| Malachite green | 11128644-Hematoporphyrin-4 | GGTGCGGGTAGGGATGAGGGCGGGTCGGGTTGAGTGTGAGCTTAGGGGTGGCGGATCTAC | Negative | Negative |
| Malachite green | 9831529-sulforhodamine B-Ma-7 | CCGGCCAAGGGTGGGAGGGAGAGGGCCGG | Negative | Negative |
| Malachite green | 18983163-ochratoxin A-24 | TCGGGTGTGGGTGGCGTAAAGGGAGCATCGG | Negative | Negative |
| Malachite green | 9831529-sulforhodamine B-Ma-18 | CCGGCCAAGGTGGAGGAGGGGCCGG | Negative | Negative |
| Malachite green | 18983163-ochratoxin A-Ma-18 | GATCGGGTGTGGGTGGCGTAAAGGGAGCATC | Negative | Negative |
| Malachite green | 17038331-codeine-4 | GGGACAGGGCTAGCAAGAATAGGATGTGGGTAAAGGTGCTGGTGAGGCAAAGCTTCCG | Negative | Negative |
| Malachite green | 23042406-Neu5Ac-6 | GCGGAAGCGUGCUGGGCCUGCAUCCCUGAGUCGCAAUCUGUUCCAGUUAAUGGAUUUCAUAACCCAGAGGUCGAU | Negative | Negative |
| Malachite green | 22261866-pesticides-1 | AAGCTTGCTTTATAGCCTGCAGCGATTCTTGATCGGAAAAGGCTGAGAGCTACGC | Negative | Negative |
| Malachite green | 15772067-isoleucine-1 | GGAUUGUGUUCCGGUAAUGAAACGAGUAAAAGAGCAAGUCC | Negative | Negative |
| Malachite green | 10978501-cholic acid-2 | AGCGCCGATTGACCCAAATCGTTTTGTATGCAAAAGCGCT | Negative | Negative |
| Fumonisin B1 | 9831529-sulforhodamine B-Ma-21 | CCGGCCAAGGGTGGGAGGGAGGAGGCCGG | Negative | Negative |
| Fumonisin B1 | 18983163-ochratoxin A-25 | ATCGGGTGTGGGTGGCGTAAAG | Negative | Negative |
| Fumonisin B1 | 17163839-Thyroxine-Ma-1 | GGUGGAGGGGGACGUGCUGCAUCCGCAGUGCGUCUUGGGUUGUG | Negative | Negative |
| Fumonisin B1 | 10978501-cholic acid-1 | GATCGAGGGCAGCGATAGCTGGGCTAATAAGGTTAGCCCCATCGGTC | Negative | Negative |
| Fumonisin B1 | 9889155-sulforhodamine B-2 | GGACGGCACCACGGUCGGAUCCGUGAGUUGUGACAAUUUAGCGGGUGGUAUUAGAGCCUACUGCCACAGCAAUAGGAUCGAUACAGAUCU | Negative | Negative |
| Fumonisin B1 | 9245404-dopamine-2 | GGGAAUUCCGCGUGUGCUGGCGGGGAGAACUUACAUGGAUUAGAGAGUGGUGCUUCAUAAGAAGGACAACUGCGUUCCAGCCGCACCCCCCCCCCAGUCCGUUCGGGAUCCUC | Negative | Negative |
| Fumonisin B1 | 23042406-Neu5Ac-2 | GCGGAAGCGUGCUGGGCCACUAUAUCGAUGCCUUUCAGACUUGGCAUAACCCAGAGGUCGAU | Negative | Negative |
| Fumonisin B1 | 20000526-arsenic-7 | ATGCAAACCCTTAAGAAAGTGGTCGTCCAAAAAACCATTG | Negative | Negative |
| Thyroxine | 9576904-cellobiose-2 | TAGCGGGTGTGGTGGGTGGGGGAGGCATGGTTTTTGGTAA | Negative | Negative |
| Thyroxine | 11128644-Hematoporphyrin-Ma-2 | ATGGTGGACGGAGATGGGACGTAG | Negative | Negative |
| Thyroxine | 11128644-Hematoporphyrin-3 | CAGCGTAAGGTCGTGGGTGGTTGGGTGCCTAAGCGTATACTTACAGCGATTGATTTGGTC | Negative | Negative |
| Thyroxine | 14561881B-isoleucine-3 | GCCANUACAUCUCGCUNUCGUACUNCG | Negative | Negative |
| Thyroxine | 17038331-codeine-2 | GGGACAGGGCTAGCACAAGAATTAGGGTCGGGAAATGGTGTGTGAGGCAAAGCTTCCG | Negative | Negative |
| Thyroxine | 17038331-codeine-21 | GGGACAGGGCTAGCAGCAAATTGGGAAAGGGATACGCTGTGTGGAGGCAAAGCTTCCG | Negative | Negative |
| Thyroxine | 8604334-LArginine-1 | AUGAUAAACCGAUGCUGGGCGAUUCUCCUGAAGUAGGGGAAGAGUUGUCAUGUAUGGG | Negative | Negative |
| Thyroxine | 9245404-Dopamine-6 | GGGAAUUCCGCGUGUGCCUUUCCAUGUAUUAGAGAGGAGAGCGCAAACACGAAGGCUCGUCGUUACAGCCGAAGAGCUCCAGUACCAUGGUUCAUGAGUCCGUUCGGGAUCCUC | Negative | Negative |
| Thyroxine | 22261866-pesticides-1 | AAGCTTGCTTTATAGCCTGCAGCGATTCTTGATCGGAAAAGGCTGAGAGCTACGC | Negative | Negative |
| FADH | 9831529-sulforhodamine B-Ma-19 | CCGGCCAAGGGTGGAAGGGAGGGGGCCGG | Negative | Negative |
| FADH | 18983163-ochratoxin A-Ma-15 | GATCGGGTGTGGGTGGCGTAAAGGGAGCATCGGACA | Negative | Negative |
| FADH | 17038331-codeine-21 | GGGACAGGGCTAGCAGCAAATTGGGAAAGGGATACGCTGTGTGGAGGCAAAGCTTCCG | Negative | Negative |
| FADH | 9245404-Dopamine-7 | GGGAAUUCCGCGUGUGCAUGGUUAAUGGAAUCCUGGGCUCCGGAGAUCACCCAUCGGAGGUAAUUUCUUUAGAAUGCUAGACGGACACUACCAGCGUGUCCGUUCGGGAUCCUC | Negative | Negative |
| FADH | 23042406-Neu5Ac-6 | GCGGAAGCGUGCUGGGCCUGCAUCCCUGAGUCGCAAUCUGUUCCAGUUAAUGGAUUUCAUAACCCAGAGGUCGAU | Negative | Negative |
| FADH | 10913311-cAMP-3 | UAAAUCGACUUGUCGCGUUGCAGGCUGCACCCCGUCCGUGGACCCCC | Negative | Negative |
| Sulforhodamine B | 18983163-ochratoxin A-22 | ATCGGGTGTGGGTGGCGTAAAGGGAGCATCGG | Negative | Negative |
| Sulforhodamine B | 18983163-ochratoxin A-9 | TGGTGGCTGTAGGTCACAGGTGGCAGATCGGGTGTGGGTGGCCTGGGAGCATCGGACAACG | Negative | Negative |
| Sulforhodamine B | 18983163-ochratoxin A-6 | TGGTGGCTGTAGGTCATCAGTCCCGATCAGGTGTGGGTGGCATTGGGAGCATCGGACAACG | Negative | Negative |
| Hematoporphyrin IX | 9831529-sulforhodamine B-Ma-3 | ATGACCAAGGGTGGGAGGGAGGGGGTCAT | Negative | Negative |
| Hematoporphyrin IX | 18983163-ochratoxin A-Ma-17 | GATCGGGTGTGGGTGGCGTAAAGGGAGCATCG | Negative | Negative |
| Hematoporphyrin IX | 18983163-ochratoxin A-Ma-15 | GATCGGGTGTGGGTGGCGTAAAGGGAGCATCGGACA | Negative | Negative |
| Hematoporphyrin IX | 9831529-sulforhodamine B-Ma-2 | CGGGATCCTAATGACCAAGGGTGGGAGGGAGGGGGTCATTAAATCCAG | Negative | Negative |
| Hematoporphyrin IX | 17038331-codeine-8 | GGGACAGGGCTAGCACCAAAAATAGGGGTAAGGGCATGGGGGTGAGGCAAAGCTTCCG | Negative | Negative |
| Hematoporphyrin IX | 17038331-codeine-4 | GGGACAGGGCTAGCAAGAATAGGATGTGGGTAAAGGTGCTGGTGAGGCAAAGCTTCCG | Negative | Negative |
| Hematoporphyrin IX | 17038331-codeine-13 | GGGACAGGGCTAGCTACAGAATAAGCGAATTAAGGGTTGGGGTGAGGCAAAGCTTCCG | Negative | Negative |
| Hematoporphyrin IX | 23042406-Neu5Ac-5 | GCGGAAGCGUGCUGGGCCUUGAAACGCUUUAUCGUUUAACGUUGCCUUCUUUGCUGUCAUAACCCAGAGGUCGAU | Negative | Negative |
| Hematoporphyrin IX | 21306108-Acetamiprid-1 | TGTAATTTGTCTGCAGCGGTTCTTGATCGCTGACACCATATTATGAAGA | Negative | Negative |
| Hematoporphyrin IX | 10743940-chitin-4 | TAGGGAATTCGTCGACGGATCCACCTGCGATAGGTAGTTTGTTTCTGTTGCCCTAGCGTCCGTCGTAACAAGTCACTCCAGGTCGACGCATGCGCCG | Negative | Negative |
